# DNA origami single crystals with Wulff shapes

**DOI:** 10.1038/s41467-021-23332-4

**Published:** 2021-05-21

**Authors:** Yong Wang, Lizhi Dai, Zhiyuan Ding, Min Ji, Jiliang Liu, Hang Xing, Xiaoguo Liu, Yonggang Ke, Chunhai Fan, Peng Wang, Ye Tian

**Affiliations:** 1 National Laboratory of Solid State Microstructures, College of Engineering and Applied Sciences, State Key Laboratory of Analytical Chemistry for Life Science, Jiangsu Key Laboratory of Artificial Functional Materials, Nanjing University, Nanjing, China; 2 Chemistry and Biomedicine Innovation Center, School of Chemistry and Chemical Engineering, Collaborative Innovation Center of Advanced Microstructures, Nanjing University, Nanjing, China; 3 National Light Source II, Brookhaven National Laboratory, Upton, NY USA; 4 Institute of Chemical Biology and Nanomedicine, State Key Laboratory of Chemo/Biosensing and Chemometrics, College of Chemistry and Chemical Engineering, Hunan University, Changsha, China; 5 School of Chemistry and Chemical Engineering, Frontiers Science Center for Transformative Molecules, and Shanghai Key Laboratory for Nucleic Acids Chemistry and Nanomedicine, Institute of Molecular Medicine, Renji Hospital, School of Medicine, Shanghai Jiao Tong University, Shanghai, China; 6 Wallace H. Coulter Department of Biomedical Engineering, Georgia Institute of Technology and Emory University, Atlanta, GA USA; 7 Department of Chemistry, Emory University, Atlanta, GA USA

**Keywords:** Self-assembly, DNA nanostructures

## Abstract

DNA origami technology has proven to be an excellent tool for precisely manipulating molecules and colloidal elements in a three-dimensional manner. However, fabrication of single crystals with well-defined facets from highly programmable, complex DNA origami units is a great challenge. Here, we report the successful fabrication of DNA origami single crystals with Wulff shapes and high yield. By regulating the symmetries and binding modes of the DNA origami building blocks, the crystalline shapes can be designed and well-controlled. The single crystals are then used to induce precise growth of an ultrathin layer of silica on the edges, resulting in mechanically reinforced silica-DNA hybrid structures that preserve the details of the single crystals without distortion. The silica-infused microcrystals can be directly observed in the dry state, which allows meticulous analysis of the crystal facets and tomographic 3D reconstruction of the single crystals by high-resolution electron microscopy.

## Abstract (web summary)

DNA origami is a valuable tool for precise manipulation of molecules in a three dimensional manner, but the design and assembly of origami units into single crystals is challenging. Here, the authors report successful fabrication of DNA origami single crystals with Wulff shapes, and regulation of their shapes by changing the symmetry and binding modes of DNA origami building blocks.

## Introduction

Single crystals formed by atoms, macromolecules and colloidal nanoparticles have become an essential part of modern material science, including laser materials, semiconductor materials, magnetic materials, etc.^1–9^. Since the 18th century, the Gibbs-Wulff rule has been widely applied to explain and predict crystal habits formed by atoms, based on the principle of thermodynamic equilibrium which claims the minimal surface energy for crystal growth^10^. However, unlike atoms with precise bond geometries and binding energies, assembly of macromolecules or colloidal building blocks into single crystals with designable crystalline morphologies remains a great challenge. DNA origami frames are sometimes treated as analogues of atoms^11–17^, because the shapes of the structures and the functional sites on them can be well-controlled by design^18–30^. Moreover, colloidal-sized particles and proteins can be precisely encaged by these DNA frames^21,25,29^. Therefore, a general approach for fabricating single crystals of colloidal building blocks with designable morphology could be initiated by first making DNA origami single crystals, as proposed by Seeman 38 years ago^31^. However, due to the size of DNA origami and the more intricate interactions between the origami structures, designing and assembling origami units into single crystals has been very challenging. Several reports have demonstrated the successful fabrication of DNA origami lattice, using polyhedral or triangular DNA origami single monomers as the building blocks^25,29^, as well as by co-assembling two different shapes of DNA polyhedra^32^, but single crystals with distinct Wulff shapes have not yet been observed.

Here, we demonstrate that DNA origami frames (DOFs) with programmable geometries and binding behaviours can be crystallized into well-defined Wulff single crystals. We illustrate our approach with regular and elongated octahedral DOFs by applying a strict slow-cooling process through the respective melting temperatures, obtaining crystals of cubic and cuboid habits accordingly with high fidelity and yield. Furthermore, the structural details of these engineered crystals can be precisely transferred to inorganic materials by controlling the growth of a very thin layer of a silica shell (~1–2 nm) on the frames^33–35^, preserving and reinforcing the crystal structures without distortion, which enables detailed structural analyses of these single crystals under long-term exposure to electron beams without disintegration of the structure. Moreover, unlike naturally occurring crystals, the morphologies of these artificial single crystals can be engineered by designing the binding modes of the DNA building blocks and the parameters of the unit cells via DNA nanotechnology. We foresee that these techniques could lead to the creation of a family of designable inorganic–organic single crystals, which could greatly expand the toolbox and potential applications of mesoporous inorganic materials by ‘duplicating’ the morphology of silica into other functional materials^36,37^.

## Results and discussion

### Crystallization of regular octahedral DNA frames

**Fig. 1:**
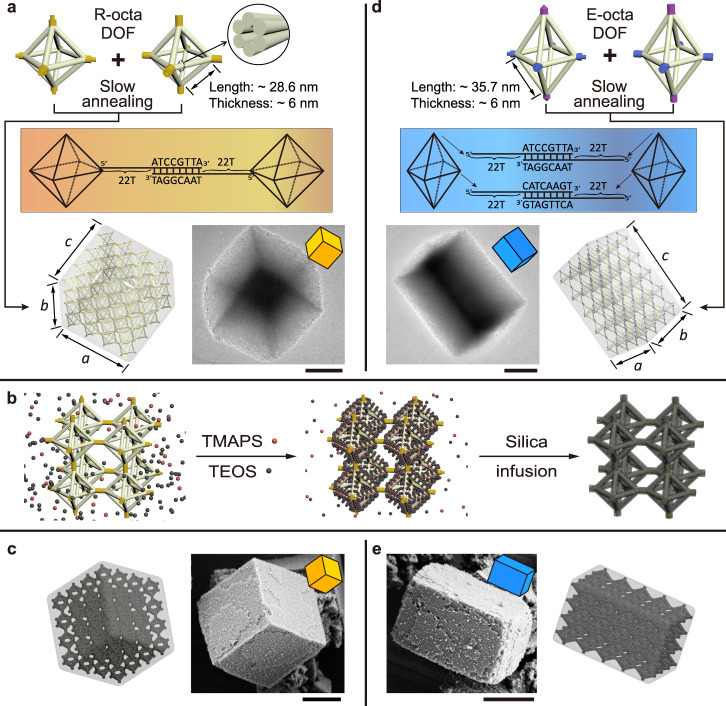
Formation of microcrystals with Wulff shapes. **a** Crystallization of cubic microcrystals (*a* = *b* = *c*) from R-octa DOFs through a slow annealing process. Middle scheme: DNA sequences of sticky ends in the R-octa system. Bottom right panel: representative TEM image of a bare microcrystal. **b** Schematic illustration of the silica-encapsulation strategy for DNA microcrystals. The process is realized through the involvement of TMAPS (red spheres) and TEOS (black spheres), see details in Methods section. **c** Model (left) and representative SEM image (right) of an encapsulated microcrystal with the same orientation. **d** Crystallization of cuboid microcrystals (*a* = *b* < *c*) from E-o*c*ta DOFs through a slow annealing procedure. Middle scheme: DNA sequences of sticky ends in the E-octa system. Bottom left panel: representative TEM image of a bare microcrystal. **e** Representative SEM image (left) and model (right) of an encapsulated microcrystal with a cuboid shape. Scale bars: 2 μm. Note that: the number of monomers drawn in the conceptual drawing in (**a** and **d**) are much less than real condition to highlight the linking mode between monomers.

The growth of Wulff single crystals was firstly investigated by using a symmetric regular octahedral (R-octa) DNA frame as the building block. Each strut of the frame is composed of a six-helix bundle of identical length (~28.6 nm), thickness (~6 nm) and molecular weight (over 4.3 MDa). To manufacture three-dimensional (3D) DNA origami crystals, 8-nt-long complementary A and B sequences were encoded in the termini of the sticky ends, binding A- and B- building blocks in the crystal arrangement (Fig. 1a and Supplementary Figs. 1, 2). We should stress that the annealing protocols are crucial for the formation of single crystals. After careful optimization, a slow annealing procedure, which enables the formation of equilibrium crystal habits with minimal surface energy, was performed for assembly of high-quality DNA crystals (Supplementary Fig. 3). Importantly, a high ionic strength (12.5 mM Mg^2+^) was required throughout the process, not only for synthesizing DOF building blocks, but also for maintaining the stability of the crystals in solution.

Negative-stained transmission electron microscopy (TEM) was then used to observe the details of the aggregates. Grains with discernible cubic shapes and features closely resembling possible facets of cubic microcrystals can be easily found, as shown in Fig. 1a and Supplementary Figs. 4–6. To preserve the overall structural configurations of the DNA large assemblies under TEM, the samples were rinsed by buffer solution (with 12.5 mM MgAc_2_) before negative-staining. However, zoomed-in observation of the edge part does not reveal highly ordered arrangements (Supplementary Fig. 7), which could be attributed to the collapse of the soft DNA crystals during the drying process. In aqueous solution, the cubic crystals are also sensitive to external conditions. For example, high temperatures or low ionic concentrations can easily damage the crystals (Supplementary Fig. 8). As a result, direct observation of the structural details of DOF single crystals can hardly be realized with electron microscopy. The DOF crystals were then used as organic templates to grow silica-DNA hybrid structures^32–34^. We discovered that a uniform thin silica layer (~1–2 nm) can be grown on the surface of DOFs, and the final silica-DNA structures precisely retain the structural details of the single crystals (Fig. 1b, see details in SI). The silica-infused DOF crystals exhibit much enhanced structural stability, enabling direct imaging of crystals with scanning electron microscopy (SEM) and TEM instruments. The stereo shapes of the cubic grains are readily identified in the dry state via SEM (Fig. 1c), exhibiting the overall sizes and geometries similar to those obtained in TEM as shown in Fig. 1a. Moreover, these microcrystals presenting different orientations are all consistent with the cubic formation, indicating that the silica coating has faithfully preserved the DNA cubic crystals in the silica-DNA replica (Supplementary Figs. 9, 10).

### Crystallization of elongated octahedral DNA frames

The crystal habits in principle can be designed by modifying the geometries and binding modes of the DNA origami building blocks. To demonstrate this concept, an elongated octahedral (E-octa) DOF with D_4h_ symmetry was designed and tested (Fig. 1d and Supplementary Fig. 11). The E-octa DOF contains twelve edges of two different lengths. The four edges in the middle-plane have the same length as the R-octa DOF (~28.6 nm), while the other eight edges are elongated to ~35.7 nm (Supplementary Fig. 12). Crystals of E-octa DOFs were also assembled from A- and B- E-octa origami units. To avoid unwanted binding modes between neighbouring E-octa DOFs (Supplementary Fig. 13), the sticky ends in the E-octa DOFs were designed to contain two different sets of sequences (purple sticky ends complementary to purple sticky ends and blue sticky ends complementary to blue sticky ends; Fig. 1d and Supplementary Fig. 14). These two kinds of E-octa DOFs were assembled separately and then mixed together by using the same thermal annealing procedure previously discussed. The observed products are cuboid-shaped DNA crystals, in good agreement with our design (Fig. 1d). More specifically, the shapes of the Wulff crystals (*a* = *b* < *c*) directly correlate with the geometry of the building block (elongated along one axis). Comprehensive views of cuboid grains after silica embedding and a sketchy model simulating the grain orientation (Fig. 1e and Supplementary Fig. 19) confirm this concept. Therefore, promisingly, by manipulating the edge length of octahedral building blocks, relevant cuboid microcrystals with a defined parameter ratio (*a*: *b*: *c*) could be anticipated to be readily obtained.

### Yields and surface defects of Wulff-shaped microcrystals

**Fig. 2:**
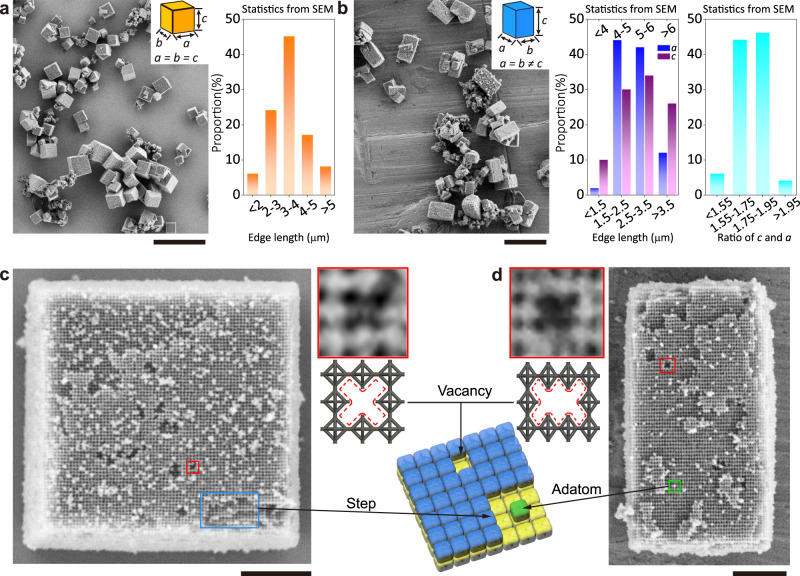
SEM images and analysis of Wulff-shaped microcrystals. **a** Left: large-scaled SEM image of cubic microcrystals. Right: size distribution of 100 microcrystals (*n* = 100), average edge length: 3.51 ± 0.98 μm (mean ± SD). Scale bar: 20 μm. **b** Large-scale SEM image of cuboid microcrystals. Middle: size distribution of 50 microcrystals (*n* = 50) in terms of *a* (blue) and *c* (purple), average edge length of *a*: 2.69 ± 0.59 μm (mean ± SD), average edge length of *c*: 4.69 ± 1.04 μm (mean ± SD). Right: distribution of the ratio between *a* (or *b*) and *c* of microcrystals. Scale bar: 10 μm. **c** SEM image of an individual cubic microcrystal. Top right panel: close-up view of a partial region with a vacancy inside (enclosed by the red square) and corresponding model. Surficial steps are labelled with blue and indicated in the schematic. Scale bar: 1 μm. **d** SEM image of an individual cuboid microcrystal. Top left panel: close-up view of a partial region with a vacancy inside (enclosed by the red square) and corresponding model. A surficial adatom is labelled with green and indicated in the schematic as well. Scale bar: 1 μm. Source data are provided as a Source Data file.

A high yield (>95%) of DNA origami crystals with cubic or cuboid Wulff shapes is evidenced by the large-scale SEM images, which show abundant microcrystals in a single image (Fig. 2a, b and Supplementary Figs. 20, 21). Visualization at low magnification by SEM proves the integral polyhedral shapes of the grains. We observed the edge lengths of the obtained microcrystals were not homogenous, which can be possibly attributed to the slow and inhomogeneous colloidal crystal growth process at this initial monomer concentration^2^. The statistics of the cubic grain size distribution of (Fig. 2a, right panel) reveal that almost half of the grains have edge lengths (*a* = *b* = *c*) centralized in the range of 3~4 μm. In contrast, for the cuboid grains which possess a geometrical morphology with partial symmetry (*a* = *b* ≠ *c*), the length ratio of the two unequal edges (*a* and *c*) shows an evident tendency of lying in the range from 1.6 to 1.9 (Fig. 2b, right panel), based on the fact that the edge lengths of the subfaces (*a* and *b*) are mostly identical (Supplementary Fig. 23).

Close surface inspection of individual cubic (Fig. 2c) and cuboid (Fig. 2d) grains deposited on a substrate was performed using SEM. The low magnification images of the whole grains show well-ordered and periodic bright contrast, which is identified as the octahedral building blocks (Supplementary Figs. 24, 25) with sizes of ~40 nm, close to the theoretical size of the DOFs. The packing mode of the high contrast DOFs is consistent with the {100} facet of the cubic crystals, demonstrating correct assembly as designed. Thereinto, typical regions enclosed by coloured rectangles display several types of common surface defects, that are frequently observed in the generated microcrystals^2^, including vacancies (enclosed by red rectangles), step edges formed among different layers (enclosed by the blue rectangle) and particle adatoms (enclosed by the green rectangle). All the defects are symbolized by a vacant box in the sketchily matched models as shown in Fig. 2c, d. Furthermore, energy-dispersive spectroscopy (EDS) mapping verifies the silica encapsulation of the microcrystals (Supplementary Fig. 26). Tilt series of SEM and TEM images were also acquired of the individual microcrystals and showed that the 3D morphologies were consistent with the cubic crystal habits (Supplementary Figs. 27, 28).

### High-resolution structural analysis of cubic grains

**Fig. 3:**
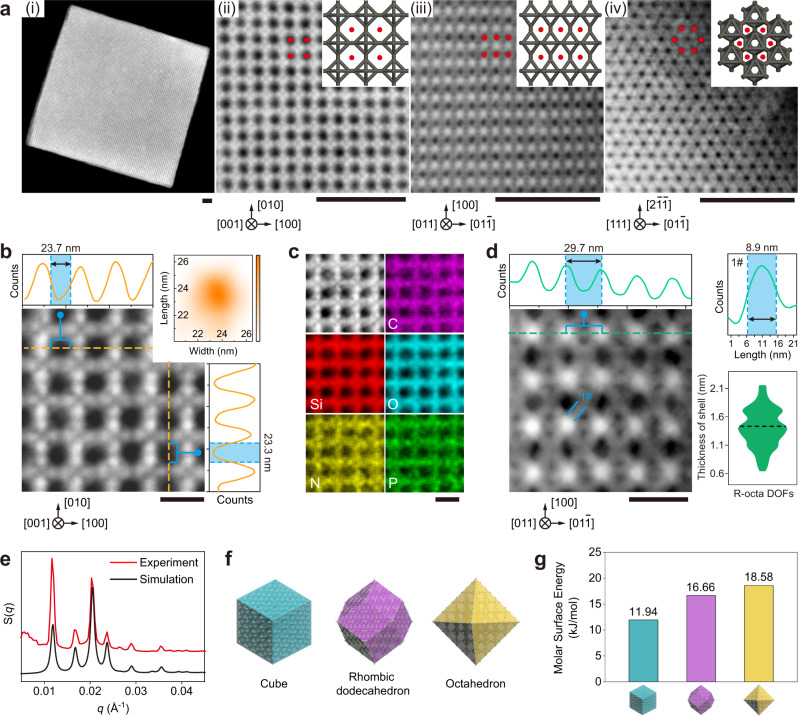
Determination of the structure and internal uniformity of cubic microcrystals. **a** HAADF-STEM image of a free-standing cubic microcrystal (i); close-up views of multiple facets (ii–iv) obtained by observation along different orientations, with models inserted in the top right panels. Scale bars: 250 nm. **b** Enlarged image of an arbitrary partial region of **a** (ii). Top and right panels: size measurements of external pores by line profile analysis. Top right panel: size distribution of external pores. Scale bar: 50 nm. **c** HR-EDS mapping of the porous structure. C, Si, O, N, and P were determined and are labelled in purple, red, cyan, yellow and green, respectively. Scale bar: 50 nm. **d** Enlarged image of an arbitrary partial region of **a** (iii), with length (top) and width (top right) measurements of silicified bundles by line profile analysis, and thickness distribution of the silica shell (bottom right). Scale bar: 50 nm. **e** Representative one-dimensional SAXS result (red curve) and fitting (black curve) of encapsulated cubic crystals. **f** Schematics of different shape Wulff polyhedra exposing different facets composed of R-octa DOFs. **g** Comparison of molar surface energies of exposed surfaces among different Wulff shapes. Source data are provided as a Source Data file.

As proposed in Fig. 1c, the silica encapsulation process should only solidify the DOFs to form 3D inorganic porous frameworks without deviating them from the real binding modes and morphologies of the microcrystals. To verify this experimentally, high-angle annular dark-field scanning transmission electron microscopy (HAADF-STEM) was employed due to the high-resolution imaging ability^38^. Supplementary Video 1 showcases the entire process of tilting experiment for an encapsulated cubic grain. A low magnification HAADF-STEM image of a [001]-oriented cubic microcrystal (Fig. 3a, (i)) reveals a square shape with an edge length of ~4 µm. High-resolution HAADF-STEM images were also acquired along the [001], [011] and [111] zone-axes (Fig. 3a, (ii)–(iv) and Supplementary Fig. 33) and clearly show that the porous motifs well match the models (inset), revealing the arrangements of R-octa DOFs for the corresponding zone-axis.

The enlarged [001] zone-axis HAADF-STEM images (Fig. 3b and Supplementary Fig. 39) show the periodic structure of the porous motifs, for which the width (Fig. 3b, top panel) and length (Fig. 3b, right panel) are measured to be 23.7 nm and 23.3 nm respectively via line profile analysis. The size statistics of the pores enclosed by the cross-organized DOFs reveal homogeneous areas of (23.1 ± 1.2) × (23.4 ± 1.1) nm^2^ (Fig. 3b, top right panel), after analysis of more than fifty positions. In addition, high-resolution energy-dispersive spectroscopy (HR-EDS) mapping confirms that the chemical composition (C/Si/O/N/P) is completely confined in the silica shell framework without diffusion into the porous channels (Fig. 3c and Supplementary Fig. 40). Accordingly, some intrinsic geometrical parameters of the DOFs were measured by line profile analysis in a partial region along the [011] zone-axis (Fig. 3d and Supplementary Figs. 42, 43), revealing that the length and diameter of bundles are increased to 29.7 nm and 8.9 nm respectively after silica infusion (Fig. 3d, top panels). Based on the statistics of the bundle diameters, the average thickness of the silica shell is calculated to be 1.4 ± 0.3 nm (Fig. 3d, bottom right panel).

**Fig. 4:**
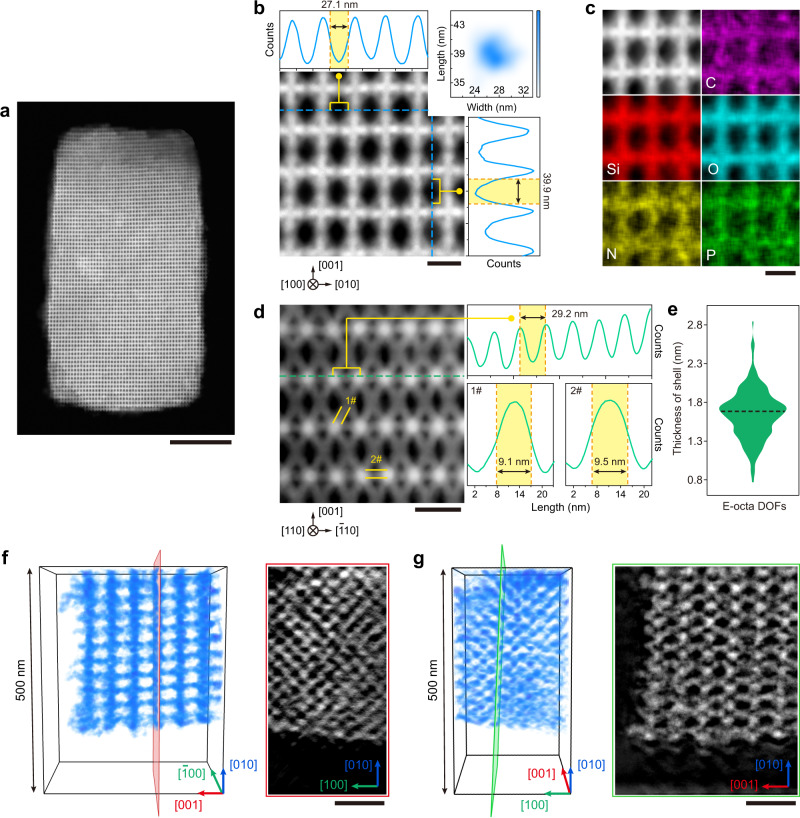
Structural analysis and reconstruction of cuboid microcrystals. **a** Representative HAADF-STEM image of an individual cuboid microcrystal. Scale bar: 1 μm. **b** Zoomed-in view of a nanoscopic region arbitrarily selected from the crystal oriented along the [100] zone-axis. Top and right panels: size measurements of pores by line profile analysis. Top right panel: size distribution of mesopores. Scale bar: 50 nm. **c** HR-EDS mapping of the mesoporous configuration. C, Si, O, N, and P are coloured in purple, red, cyan, yellow and green, respectively. Scale bar: 50 nm. **d** Left: zoomed-in image of nanoscopic partial region observed along the [110] zone-axis. Right: length (top panel) and width (bottom panels) measurements of silicified bundles by line profile analysis. Scale bar: 50 nm. **e** Thickness distribution of the silica shell encapsulated on cuboid crystals. **f**, **g** 3D reconstruction of a piece of a microcrystal. The 3D rendered results are exhibited along two perpendicular axes and the cross-sectional images were extracted from the red (**f**) and green (**g**) planes along different directions. Obtained tomographic images are enclosed by corresponding coloured rectangles. Scale bars: 250 nm.

To investigate the internal arrangements of the R-octa DOFs inside the cubic single crystals, small-angle X-ray scattering (SAXS) measurements were applied after the silica encapsulation process. The function of S(*q*) (extracted structure factor) vs *q* (scattering vector) reveals several distinguishable resolution-limited Bragg peaks, as shown in Fig. 3e, demonstrating successful crystallization of DOFs in a 3D manner. It also proves that the silica encapsulation process does not affect the packing mode and long-range order of the single crystals. The crystallite sizes can be calculated from the Scherrer formula *D* = *Kλ/B*cos*θ* ≈ 4.1 μm, which is similar to the grain size observed in SEM as shown in Fig. 2a. The one-dimensional curve well matches the fitting result (black curve shown in Fig. 4e) obtained by applying R-octa units as the building block with bundle thickness equal to 9 nm (Supplementary Fig. 45), which explains the in situ integrated stabilization of the microcrystals by the strategy of silica encapsulation, without detectable damage.

Moreover, the formation of cubic microcrystals could be accomplished and rationalized by the parameter γ (surface energy) calculated for the exposed facets. Three traditional Wulff shapes of the microcrystals: cube, rhombic dodecahedron and octahedron exposing the {100}, {110} and {111} facets, respectively (Fig. 3f and Supplementary Fig. 47), could be formed by applying R-octa as the building block, while maintaining the internal binding mode of DOFs the same as that shown in Supplementary Fig. 2. According to the rule of a standard broken-bond model, minimal exposure of unconnected vertices per unit area contributes to thermodynamically favoured state^39^. By assuming equivalent volumes for these three proposed Wulff shapes of microcrystals, the relative surface energy γ exhibits a ratio of γ{100}:γ{110}:γ{111} = 1.00:1.40:1.56 (see details in Supplementary Figs. 48–50), which predicts the equilibrium Wulff polyhedron to be a cube with only {100} facets exposed, well matching the experimental results. With the binding energy of 8-bp complementary sticky ends (Supplementary Fig. 50a) estimated to be 58.52 kJ/mol, the molar surface energies of the three imagined crystal habits are calculated to be 11.94 kJ/mol (cube), 16.66 kJ/mol (rhombic dodecahedron) and 18.58 kJ/mol (octahedron), respectively (Fig. 3g). According to Gibbs-Wulff theory^10^, the cube habit with the lowest surface energy is a thermodynamic equilibrium habit.

### High-resolution structural analysis of cuboid grains

Figure 4a shows a typical HAADF-STEM image acquired from a [100]-oriented free-standing cuboid microcrystal with apparent lattice fringes, suggesting a highly ordered arrangement of E-octa DOFs (see also Supplementary Fig. 51). An enlarged image from an arbitrary partial region (Fig. 4b and Supplementary Fig. 52) shows distinct elongation of the porous structures along the [001] direction, which is further revealed by line profile analysis (Fig. 4b, top and right panels). The mean area of the mesopores increases to (39.0 ± 1.4) × (27.2 ± 1.7) nm^2^ (Fig. 4b, top right panel) due to the elongation of the building blocks along the corresponding axis. Nevertheless, the porous structures still maintain the nanometre-level uniformity of the elemental distribution shown in the HR-EDS mappings (Fig. 4c and Supplementary Fig. 53). Similarly, in addition to mesopores, other configurational features can also be preserved based on the view along [110] zone axis, because of the thin layer of silica coated on the DNA bundles, with a thickness of 1.7 ± 0.3 nm (Figs. 4d, 4e and Supplementary Figs. 55–57). Moreover, Supplementary Video 2 shows the tilting experiment of the edge part of a discrete encapsulated cuboid single-crystal visualized via HAADF-STEM.

Furthermore, electron tomography was used to produce detailed 3D images of the internal part of the cuboid single crystal with a volume of ~0.37 × 0.34 × 0.25 µm^3^ (Supplementary Fig. 58), by recording two tilt series of projections along mutually perpendicular tilt axes (Supplementary Fig. 59 and Supplementary Videos 3, 4). The packing mode of the E-octa DOFs is clearly visualized in the volume rendering from the two different viewing directions (Fig. 4f, g, left panels). Furthermore, cross-sectional views along two mutually perpendicular orientations (Fig. 4f, g, right panels) verify the uniform structure inside this fragment. The included 3D animations (Supplementary Videos 5–9) clearly show each monomer unit in this cuboid grain, indicating not only the strictly ordered arrangement of monomers along the [100] and [001] orientations, but also the regularity of the core section over a long micro-scale range. Notably, the entire structure of the observed grain shows robustness sufficient for constant exposure to focused beam irradiation over 3 h, attributed to the silica matrix encapsulation.

In conclusion, we have experimentally and theoretically demonstrated the successful fabrication of Wulff equilibrium crystals from DNA origami units. The relationship between the geometry of the nanoscale DNA origami building blocks and the shape of the corresponding microcrystals was enlightened through the shown cases of R-octa and E-octa, providing a promising route for creating arbitrary single crystals with the desired Wulff shape. Moreover, since the bond energy of the chosen sites on the DNA origami building blocks with their neighbours can be artificially manipulated by tuning the number of pairing bases, the growth rate of different crystal facets can be thermodynamically controlled during the crystallization process, which provides another way to define the shapes of Wulff crystals. In addition, an accurate infusion process could establish a feasible methodology in the manufacture of diverse hybrid materials. The conformations of DNA single crystals could be duplicated with nanometre-level precision into other functional frameworks with designated components, which would bring insights and developments to the catalysis, optics, and other interdisciplinary fields.

## Methods

### Design and synthesis of R-octa and E-octa DNA origami frames

R-octa and E-octa DOF are predesigned with caDNAno software (http://cadnano.org/)^40^. For R-octa DOFs, each edge of the frame is composed of a six-helix bundle (6HB) with length of 84 base pairs (bps), while for E-octa DOFs, eight edges above and below the middle plane are elongated into 105 base pairs (bps). One single-strand DNA (sticky end) is designed to be extended from both ends of each edge, so four sticky ends stretch out from each vertex. Partially complementary bases of sticky ends will trigger the binding with other homogeneous DOF monomers. Structural parameters of the pre-designed DOFs are shown in details in Supplementary Fig. 12. The DOF monomers are synthesized using M13mp18 DNA as the scaffold followed by mixing with staple oligonucleotides (see Supplementary Tables), buffer, and salts. Synthetic procedure is carried out by carefully treating the mixed solution with thermal annealing process from 95 °C to 20 °C for ~20 h in a PCR device. Representative negatively stained TEM images of obtained samples are shown in Supplementary Figs. 1, 11.

### Fabrication of cubic and cuboid microcrystals

Two kinds of octahedral DOFs carrying partially complementary ‘sticky ends’ are employed in equal molar amount for fabrication of microcrystals, which is accomplished through thermal annealing process. In a typical procedure, annealing process is conducted from 50 °C to 20 °C with the rate of −0.2 °C/h to ensure the formation of thermodynamically equilibrium products.

### PEG precipitation process

For PEG precipitation, PEG buffer (20% m/v) should be prepared at first. Twenty grams of PEG lumps (Mw: 8000 g/mol or 10,000 g/mol) were dissolved in 100 mL buffer solution (2 × TE, 500 mM NaCl), in which 20-min ultrasonic treatment can help accelerate the dissolution process of PEG. Prepared DNA origami solution was mixed with the same account of PEG buffer in 1.5 mL centrifuge tube and was then centrifuged at 20,000 rcf for 3 h. The supernatant was then removed. The condensed DNA origami sediment attached at the bottom was mixed with desired buffer solution (usually 1 × TAE, 12.5 mM MgAc_2_) and redispersed after shaking at a constant temperature overnight (1000 rpm, 12 h, 37.5 °C).

### Silica infusion process

The specific approach of silica shell growth is performed according to the slightly modified Stöber method which contains a series of hydrolysis and condensation process. Encapsulation is initiated by washing samples at room temperature through removing the supernatant and filling fresh 1×TAE buffer which contains 7 mM MgCl_2_. After treating for several rounds, concentration of Mg^2+^ in solution has been decreased prominently. Then the sample is mixed with TMAPS at room temperature and shaken on thermomixer (350 rpm, 20 min). TMAPS is utilized as a co-structure directing agent (CSDA), which can accumulate on the phosphate group of dsDNA backbone under the favourable condition which created by decreased Mg^2+^ concentration, and the co-condensation sites for silica precursor are provided through the adsorption onto DNA backbone. Subsequently, TEOS is added followed by shaking for another 30 min at the same rate. For two silanes we used, the molar ratio of nucleotide: TMAPS: TEOS is ~1:10:20. Silanes are both diluted with methanol in the procedures described above according to the amount of samples in solution. After the above process are completed, the sample mixed with silanes is left statically for over 10 h at room temperature, during which the cloudy precipitate formed at the bottom of the tube, which indicates microcrystals have undergone solidification and silica debris is simultaneously generated due to the excess amount of the silanes in solution. Since the spatially organized nucleation sites for silicification is presented by absorption of TMAPS to uniform-distributed negative charges on the microstructure, the formation of Si–O–Si bonds are fulfilled directly on the fundamental DNA structure and the entire bare grains are encapsulated by the amorphous silica shell.

### 3D reconstruction method

The 86 projections of two orthogonal tilt axes with a field of view of 500 × 500 nm for tomographic reconstruction are collected on a double aberration-corrected FEI Titan cubed G2 60–300 S/TEM using FEI tomography software in HAADF-STEM mode. A series of projections are collected from −64 to +50 degrees and from −42 to +52 degrees about two perpendicular tilting axes. Projections are taken every 4 degrees at the angle lower than ±20 degrees and every 2 degrees at the angle higher than ±20 degrees. The projections are subsequently processed using IMOD^41^. After the alignment, back-projection algorithm is used to generate the 3D reconstructions from the projections of two tilt axes. The logarithm of the projections and a Gaussian filter (cutoff of 0.35 and sigma of 0.035) are used in the algorithm. The results of two tilt axes are further combined into a final 3D rendered image in IMOD. The 3D image for visualization is rendered by Avizo. The voxel size of the 3D reconstruction is 1 nm.

## Supplementary information

Supplementary Information

Description of Additional Supplementary Files

Supplementary Video 1

Supplementary Video 2

Supplementary Video 3

Supplementary Video 4

Supplementary Video 5

Supplementary Video 6

Supplementary Video 7

Supplementary Video 8

Supplementary Video 9

## Data Availability

All data are available within the manuscript and supplementary information. Additional data are available upon reasonable request, sent to the corresponding author: ytian@nju.edu.cn. Source data are provided with this paper.

## Source data

Source Data
