# Supplementary material for: DNA origami single crystals with Wulff shapes
Source: Nat Commun. 2021 May 21;12:3011. doi: 10.1038/s41467-021-23332-4 (PMC8140131; doi:10.1038/s41467-021-23332-4)
Supplement: Supplementary file 1 — Supplementary Information [file 41467_2021_23332_MOESM1_ESM.pdf]

DNA origami single crystals with Wulff shapes

Supplementary Information

Yong Wang,[1,2,#] Lizhi Dai,[1,2,#] Zhiyuan Ding,[1,#] Min Ji,[1,2] Jiliang Liu,[3] Hang Xing,[4] Xiaoguo Liu,[5] Yonggang Ke,[6,7] Chunhai Fan,[5] Peng Wang,[1] and Ye Tian[1,2*]*

[1]National Laboratory of Solid State Microstructures, College of Engineering and Applied Sciences, State Key Laboratory of Analytical Chemistry for Life Science, Jiangsu Key Laboratory of Artificial Functional Materials, Nanjing University, Nanjing, 210093, China

[2]Chemistry and Biomedicine Innovation Center, School of Chemistry and Chemical Engineering, Collaborative Innovation Center of Advanced Microstructures, Nanjing University, Nanjing, 210023, China

[3]National Light Source II, Brookhaven National Laboratory, Upton, NY, 11973, United States

[4]Institute of Chemical Biology and Nanomedicine, State Key Laboratory of Chemo/Biosensing and Chemometrics, College of Chemistry and Chemical Engineering, Hunan University, Changsha, 410082, China

[5]School of Chemistry and Chemical Engineering, Frontiers Science Center for Transformative Molecules, and Shanghai Key Laboratory for Nucleic Acids Chemistry and Nanomedicine, Institute of Molecular Medicine, Renji Hospital, School of Medicine, Shanghai Jiao Tong University, Shanghai 200240, China

[6]Wallace H. Coulter Department of Biomedical Engineering, Georgia Institute of Technology and Emory University, Atlanta, Georgia, 30322, United States

[7]Department of Chemistry, Emory University, Atlanta, Georgia, 30322, United States

[#]These authors contributed equally: Yong Wang, Lizhi Dai, Zhiyuan Ding.

*e-mail address: ytian@nju.edu.cn; wangpeng@nju.edu.cn

Catalogue

Supplementary Methods

a. SAXS Modeling

Supplementary Notes

a. Transmission electron microscopy (TEM) specimen preparation
b. Scanning electron microscopy (SEM)
c. Scanning/transmission electron microscopy (S/TEM)
d. Energy dispersive spectroscopy (EDS)
e. Small angle X-ray scattering (SAXS)

Supplementary discussion

a. Configuration determination of unencapsulated microcrystals
b. Stability of unencapsulated microcrystals
c. Monomer concentration influence on crystal size
d. Orientation of samples and tilting experiments
e. Evaluation of structural fidelity after silica infusion process
f. Internal arrangement of building blocks in microcrystals
g. Surface energy calculation for R-octa DOFs system

Supplementary Tables (1-4)

Supplementary Figures (1-60)

Supplementary Tables (1-4)

Supplementary References

Supplementary Methods

a. SAXS modeling

The structures of 3D lattice of DNA materials voxels are confirmed by SAXS experiments. The experiments are carried at SSRF beamlines BL16B2 and BL19U2. The SAXS data are collected with the following experimental parameters. Distance of sample to detector is 2215.5 mm, wavelength is 0.9184 Å and pixel size of detector is 172×172 microns. The raw data are obtained in the form of 2D patterns and are integrated into 1D profiles as a function of *q*, where $q = 4\frac{\pi}{\lambda} sin\,(\frac{\theta}{2})$ and θ is scattering angle, which can be determined by detector pixel size and distance of sample to detector.

The 1D intensity profile, $I(q)$, is reduced from azimuthal averaging of 2D SAXS pattern. After background corrections, the structure factor, $S(q)$, can be calculated as 1D intensity profile $I(q)$ divided by isotropic form factor $P(q)$. The calculation had been well studied[1-3]. Here the detailed calculation of $S(q)$ is presented below:

$$S(q) = \frac{CZ_0(q)}{P(q)} G(q) + [1 - \beta(q) G(q)],$$

The structure factor is constituted by a structural term, $\frac{CZ_0(q)}{P(q)} G(q)$, and a diffuse term, $1-\beta(q)G(q)$. The $Z_0(q)$ of structural term are determined by convolving the peak shape function $L(q-q_{hkl})$ with reciprocal lattice vector $F(q_{hkl})$. The calculation of $Z_0(q)$ can be written as:

$$Z_0(q) = \frac{1}{q^2}\sum_{hkl}^{m_{hkl}} \left|\sum_{j=1}^{N} \langle F_j(q_{hkl})\rangle \exp\left[2\pi i\left(x_j h + y_j k + z_j l\right)\right]\right|^2 L(q-q_{hkl})$$

The peak profile is Lorentz function. C is scaling factor. The Debye–Waller thermal parts, $G(q)$, are defined as: $G(q) = e^{-\sigma_D^2 q^2 a^2}$, where σ_D is Debye–Waller factor, a is the smallest lattice constant of the unit cell. The Debye–Waller thermal parts are countered for intensity decay due to thermal vibration in the lattice.

The $P(q)$ is the isotropic form factor of $F(q)$. $F(q)$ is the form factor of composite objects, which is an octahedral object built by silica coated DNA bundles at each edge. The DNA bundles are modeled as a cylinder model with uniform density and radius of 4.5 nm. The form factor of cylinder is:

$$F_{\text{cylinder}}(q) = 2sinc(q_z h \frac{\pi}{2}) \frac{J_1(q_r r)}{q_r r}$$

$$q_r = \sqrt{q_x^2 + q_y^2}$$

where J_1 is the first order of the Bessel function of the first kind.

The octahedral object consists of the shifted and rotated cylinders, the form factor can be written as:

$$F(q) = \sum_{j \in \text{cylinders}} F_j(r_j \vec{q} \cdot R_j^T) e^{i\vec{q}\Delta\vec{r}_j}$$

where $\vec{r}_j$ and $\vec{R}_j$ are the correlated shift and rotation transformation of each cylinder model. Then the isotropic form factor, $P(q)$, can be determined as:

$$P(q) = \langle F(q) \rangle_o$$

where $\langle \ \rangle_o$ is averaging of all orientations.

Supplementary Figure 44 shows that the octahedral object composed of cylinders. The variations of length and diameter of cylinder will affect the form factor and structure factor of octahedra. The increase of radius will significantly augment the amplitude for both form factor and structure factor.

The $\beta(q)$ of diffuse term correlates to the polydispersity of nanoparticle. The $\beta(q)$ expresses same exponential decay effect as Debye–Waller function. As discussed in published literature[3], the $\beta(q)$ have been omitted in the calculation of structure factor here. Thus, the structure factor can be rewritten as:

$$S(q) = \frac{CZ_0(q)}{P(q)} G(q),$$

We optimize the Python package – ScatterSim, which is implemented in the published literature[3], to fit the silica coated cubic microcrystals. Supplementary Figure 45 exhibits the fitting of SAXS data of silica coated cubic microcrystals. The fitting results indicate that the silica coated octahedra have the edge length of 30 nm and the radius of cylinders at edges are 4.5 nm.

Supplementary Notes

a. Transmission electron microscopy (TEM)

For the preparation of specimen, the carbon coated copper grids are firstly glow discharged for 30 s and held for another 10 s under vacuum in PELCO easiGlow instrument (Ted Pella, Inc.). Then a drop containing approximate 5 μL sample is dropcasted onto the treated grid and incubated for 5 min. The un-dried solution remaining on the grid after the incubation is handled through blotting the grid with filter paper. Subsequently, A drop of fresh buffer (containing 40 mM Tris-acetate, 1 mM EDTA and 12.5 mM $MgAc_2$) is applied to the surface of grid and then wicked away for two times to remove the salts and any other supernumerary component, the suite of operations is followed by negatively staining the specimen with 5 μL 2% (w/v) uranyl acetate aqueous solution for 10~15 s in order to achieve visualization of DOF monomers during the characterization process.

For fabricated microcrystals, the operation of removing the supernatant and filling fresh buffer are executed for several times to discard DOF monomers which do not participate in the assembly and this relieves the interference brought by the excess reactant under the imaging environment. A drop of condensed microcrystals sitting in the bottom of the centrifuge tube is extracted, dripped onto the glow-discharged grid and incubated for 8~10 min. Then the fresh buffer is dripped onto the grid to infiltrate the sample and the excess solution is blotted with filter paper. Negative staining is also conducted using 2% (w/v) uranyl acetate aqueous solution for 10 s. Since the sample is exposed to the liquid with requisite cation concentration throughout the preparation, the entire structure is left intact.

For encapsulated microcrystals, excess silanes and silica chunks are removed by discarding supernatant and adding deionized water to make the sample resuspended. The process is carried out for several rounds and then the preparation of deposited sample is completed in the similar way as for the unencapsulated microcrystals. Solidified grains are simply washed with deionized water after the incubation on the grid and negative staining is not required.

TEM images are obtained on JEM-2100 (JEOL), JEM-2800 (JEOL) and Tecnai F20 (FEI) transmission electron microscopes, respectively, operated at an accelerating voltage of 200 kV.

b. Scanning electron microscopy (SEM)

SEM imaging is primarily adopted for the observation of solid-state microcrystals. 5 μL of turbid liquid of the sample is dropped onto the silicon wafer directly and then

the droplet is dried under infrared light in order to fulfill the requirements of imaging.

SEM images are obtained on Gemini Ultra Plus (Zeiss), Gemini Ultra 55 (Zeiss), JSM-7800F (JEOL) and Verios G4 UC (FEI), respectively. Specific accelerating voltages and corresponding working distances depend on the degree of charge accumulation and required magnification.

c. Scanning transmission electron microscopy (STEM)

STEM images are obtained on a double aberration-corrected FEI Titan cubed G2 60-300 STEM operating at the accelerating voltage of 300 kV, and a FEI Tecnai F20 at the accelerating voltage of 200 kV.

d. Energy dispersive spectroscopy (EDS)

EDS mappings shown in Supplementary Fig. 26 are performed at 20 kV on Zeiss Ultra Plus FE-SEM instrument. Furthermore, high resolution EDS (HR-EDS) mappings (Figs. 3c, 4c and Supplementary Figs. 40, 53) are conducted on FEI Titan cubed G2 60-300 instrument under STEM mode.

e. Small angle X-ray scattering (SAXS)

All SAXS experiments are conducted at beamlines BL16B1 and BL19U2 at Shanghai Synchrotron Radiation Facility (SSRF). The scattering data are collected with collimated X-rays calibrated against a silver behenate standard sample and exposure time is set as 5 s for the collection of experimental data. Briefly, 50 μL of the sample solution is transferred to a quartz capillary with the inside diameter around 1 mm. Capillaries are sealed with wax before SAXS measurements and are fixed on the sample stage carefully. The scattering data are collected with a Dectris Pilatus 2M pixel-array detector. The silicified microcrystals are investigated by SAXS to identify the internal structural uniformity and confirm the maintaining of original configurations throughout the encapsulation.

Supplementary Discussion

a. Configuration determination of unencapsulated microcrystals

It is conventionally considered that the structural rigidity of soft matter which represented by DNA monomers is unfavorable to maintain their inherent configurations in case detached from solution-phase. Although several images revealed the phenomenon that the entire geometric shape of some bare microcrystals can be kept under penetration of electron dose and vacuum state during observation (Supplementary Figs. 4-7, 16-18), the connection of DOF monomers at the edge of the grain (Supplementary Fig. 7) is difficult to identify. We cannot conclude from the images that the formation of Wulff shaped microcrystals is governed by the packing of DOF monomers. To restore the intrinsic morphology of microcrystals in solution and simultaneously prove the Wulff polyhedral configuration is determined by proposed packing mode of DOFs (Supplementary Figs. 2, 14), encapsulation method is needed.

b. Stability of unencapsulated microcrystals

As mentioned above, stereo structure of bare DNA microcrystals without any reinforcement can only be maintained in solution-phase under normal temperatures. For microcrystals in this work, fidelity of crystalline configuration relies on the Mg^{2+} concentration in addition to the rigidity of DNA motifs. For verifying the Mg^{2+} density dependence of structural regularity, concentration of Mg^{2+} is decreased via the ion exchange process, which is realized by discarding supernatant and filling the buffer containing 5 mM $MgCl_2$. After looping the operation for many cycles, Mg^{2+} concentration of sample solution is decreased to approximate 5 mM. As shown in Supplementary Fig. 8, collapse and deformation has emerged visibly for unencapsulated microcrystals. Shapes of corners and edges are distorted and grains are surrounded by densely distributed dark dots which are confirmed to be negatively stained R-octa DOFs detached from grains. It can be speculated that the size will further shrink and cohesion of DOF monomers will gradually dissociate in case of further decrease of Mg^{2+} density. Structural deformation is irreversible even if Mg^{2+} density is raised to regular condition. Similar irreversible destroy of the DNA single crystals could also be observed when increasing temperatures of the solution above ~40 °C.

c. Monomer concentration influence on crystal size

In our manuscript, the most commonly monomer concentration is 20 nM (using PEG precipitation method) for cubic DNA origami crystals and 10 nM for cuboid DNA origami crystals.

In terms of cubic DNA origami crystals, monomer concentration is probably positively related to the crystal size. As shown in Supplementary Fig. 22, the average sizes of cubic crystal grains made of monomers at different concentrations are 2.10 ± 0.39 μm for 10 nM, 3.57 ± 0.99 μm for 20 nM, 3.88 ± 1.09 μm for 40 nM and 4.56 ± 1.17 μm for 60 nM. Besides, cubic grains over 6 μm tend to be more easily found when the concentration increases, even grains over 10 μm can be captured in 40 nM and 60 nM samples, while grains over 3 μm can hardly be seen in 10 nM samples.

d. Orientation of samples and tilting experiments

After being solidified by silica shell, the weight and mechanical strength of microcrystals will be significantly promoted. Differ from bare grains, the reinforced stiffness makes individual crystals be inclined to keep the face-up state after ultimately depositing on the substrates (carbon coated copper grids or aluminium/silicon wafers). Images (Supplementary Fig. 10) has shown representative free-standing grains which take the orientations on two kinds of substrates. It can be observed that the orientations taken by single grains are with visibly tendency other than branched microcrystals, nearly all discrete grains (labelled with red circles in the bottom row of Supplementary Fig. 20) are face-up on silicon wafers. It will obstruct the comprehensively observation towards other facets of the entire microcrystals, particularly via TEM instruments due to the deficiency of depth of field in imaging and that is why suites of tilting experiments are performed (Supplementary Figs. 27, 28). Corresponding operations are firstly carried out on SEM instrument aiming at two cubic grains on substrates (Supplementary Fig. 27). During observation under TEM instrument, two free-standing grains are driven to rotate along the *x*- and *y*-axis successively (Supplementary Fig. 28). Furthermore, tilting experiments are also conducted under HAADF mode for cubic and cuboid microcrystal and corresponding images are composited into Supplementary Videos 1, 2, respectively.

e. Evaluation of structural fidelity after silica infusion process

Due to the motivation of strengthening the stiffness of bare DNA microcrystals and restoring the authentic status of micro-scaled objects, immobilizing assembled structures into solid-state with silica shell is implemented and characterized. Encapsulation is accomplished according to mentioned protocols and it has been verified that the proposed packing mode is reserved with high fidelity.

f. Internal arrangement of building blocks in microcrystals

EM images adequately reveal the highly ordered arrangement of building blocks

on the surface and at the edge of microcrystals. However, attributing to the large number of layers stacked by DOF monomers, conventional characterization methods (TEM/SEM) seem difficult to directly observe the internal arrangement of the building blocks due to the weak penetration of beam doses. Therefore, SAXS experiments are executed to determine the internal uniformity of micro-sized solid grains. Furthermore, a small grain is observed under STEM and a corner of which is primarily observed (Supplementary Figs. 58, 59). 3D reconstruction (Figs. 4g, 4f in main text and Supplementary Videos 5-7) for the selected region and the result of SAXS experiment have shown the ordered arrangement of encapsulated DOFs inside the microcrystals.

g. Surface energy calculation for R-octa DOFs system

(1) Definition

In terms of DOFs system, surface energy refers to excess energy from unbound sticky ends (Supplementary Fig. 48) at the surface of crystals comprised of specific number of monomers compared to those inner segments sharing the same volume.

(2) Assumption

In order to simplify calculating, we adopted some ideal assumptions as following:

(1) Only targeted crystal habit can be fabricated, any other intermediate products or by-products are not permitted to exist, for all possible shapes of Wulff polyhedra discussed here.

(2) Each crystal is perfect without any crystal flaws. According to the observation as shown in Fig. 2, defects always exist. However, compared to the entire free-standing single crystal, the part of defects can be neglected.

(3) All generated crystals share the same size. In the discussion part, we used the averaged crystal size as shown in Fig. 2 for cubic systems. For octahedral and rhombic-dodecahedral Wulff crystals, we assume they shared the same volume with the cubic single crystals.

(3) Calculation formulae

It has been fully proved by electron microscope images that R-octa DOFs shows cube-shaped habit. The average size of cube-shaped crystals reaches up to 3.51 μm, and the effective distance between two nearest neighbor octahedron building blocks is 53.2 nm according to previous record. Therefore, there are almost 66 octahedron building blocks along one edge of a cube. The number of DOFs which comprise of one cube-shaped crystal can be easily calculated by this formula:

$$C(n) = n^3$$

where $C(n)$ means the total number of DOFs, while n represents the number of DOFs along one edge of the cube.

Besides, we imagined two other crystal habits—rhombic-dodecahedron-shaped habit and octahedron-shaped habit exposing {110} and {111} planes as the surfaces, respectively—as targeted crystal habit to compare their surface energy with cube-shaped habit. For these two imaginary habits, the relationship between the number of DOFs and diameter n obeys the following equations:

$$R(n) = \frac{1}{4}n^3 + \frac{1}{4}n + \frac{1}{2}\sin(\frac{n\pi}{2})$$

$$O(n) = \frac{1}{6}n^3 + \frac{5}{6}n$$

where $R(n)$, $O(n)$ means the total number of DOFs in rhombic-dodecahedron-shaped habit and octahedron-shaped habit respectively, while n represents the number of DOFs along their diameter direction (Supplementary Fig. 49a).

DOFs of different position in crystal which have different numbers of unbound sticky ends make varying degrees of contribution to surface energy. The number of DOFs on the surface layer of three habits above can be deduced by:

$$C_s(n) = C(n) - C(n\text{-}2)$$

$$R_s(n) = R(n) - R(n\text{-}2)$$

$$O_s(n) = O(n) - O(n\text{-}2)$$

where $C_s(n)$, $R_s(n)$, $O_s(n)$ denote the number of DOFs on the surface of cube-shaped, rhombic-dodecahedron-shaped and octahedron-shaped habit, respectively.

(4) Distribution of DOFs with different number of unbound sticky ends

According to statistics, we considered that the cube-shape habit is most stable when n equals to 66. Therefore, a cube-shaped crystal grain of this size consists of 287,496 DOFs. We compared the three kinds of habit when the numbers of DOFs are similar. The detailed number is derived from: $C(66)$=287,496, $R(105)$=289,432, $O(119)$=280,959.

DOFs of different position in crystals make varying degree of contribution to surface energy. The distribution of DOFs at different position is listed in Supplementary Fig. 49b.

(5) Surface Energy Calculation

Surface energy value is directly concerned with the number of unbound sticky ends, if the binding energies of them are all the same. We compared the surface energy of cube-shaped habit, rhombic-dodecahedron-shaped habit and octahedron-shaped habit by counting the number of unbound sticky ends of them on their surface. We

estimated the binding energy of the DNA sequence in sticky ends we designed on IDT Oligo Analyzer Tool (Supplementary Fig. 50a) (https://sg.idtdna.com/calc/analyzer).

Afterwards, we calculated the molar surface energy of the three habits in the solution with the magnesium salt to be 12.5 mM (a stated system containing 1 mole DOFs, listed in Supplementary Fig. 50b). Under similar conditions, cube habit has the lowest surface energy and releases the most energy during crystallization, which illustrated the formation of cube shape for R-octa system.

According to our primitive surface energy calculating model, surface energy will be decreasing until all the monomers assemble into one large grain, which is actually quite difficult to achieve. Under experimental condition, with the growing of crystal grains, their negative charge density keeps increasing as well, making it harder to combine with next monomer, accounting for limited crystal size to some extent.

Other parameters, such as monomer concentration, Mg^{2+} concentration and sticky end sequence, also contribute to the growth of DNA origami crystal. Taking sticky end sequence as an example, the DNA sequences of A- and B- monomer in R-octa system are 5'-ATCCGTTA-3' and 5'-TAACGGAT-3', whose binding energy is calculated to be 58.52 kJ/mol. If all sequences are altered by 5'-ATCCGTTGTA-3' and 5'-TACAACGGAT-3', whose binding energy rises to 72.29 kJ/mol, the released energy of crystal grains of similar size will correspondingly increase from 690.23 kJ/mol to 852.64 kJ/mol, though the ratio of surface energy and overall energy is constant. Meanwhile, the surface energy rises from 11.94 kJ/mol (8-nt sticky ends) to 14.74 kJ/mol (10-nt sticky ends), which perhaps refers to larger growth trend on the crystal surface. However, 10-nt sticky ends will inevitably raise the temperature of annealing procedure, which will exceed the melting temperature of DNA origami itself (i.e. the melting temperature of binding between scaffolds and staples). Therefore, in terms of sticky ends, the selectable range is relatively narrow than thought.

Supplementary Tables

Supplementary Table 1. Staples strands of R-octa frames. All the sequences are present from 5'-3' (left to right).

R-octa-staple-1	TCAAAGCGAACCAGACCGTTTTATATAGTC
R-octa-staple-2	GCTTTGAGGACTAAAGAGCAACGGGGAGTT
R-octa-staple-3	GTAAATCGTCGCTATTGAATAACTCAAGAA
R-octa-staple-4	AAGCCTTAAATCAAGACTTGCGGAGCAAAT
R-octa-staple-5	ATTTTAAGAACTGGCTTGAATTATCAGTGA
R-octa-staple-6	GTTAAAATTCGCATTATAAACGTAAACTAG
R-octa-staple-7	AGCACCATTACCATTACAGCAAATGACGGA
R-octa-staple-8	ATTGCGTAGATTTTCAAAACAGATTGTTTG
R-octa-staple-9	TAACCTGTTTAGCTATTTTCGCATTCATTC
R-octa-staple-10	GTCAGAGGGTAATTGAGAACACCAAAATAG
R-octa-staple-11	CTCCAGCCAGCTTTCCCCTCAGGACGTTGG
R-octa-staple-12	GTCCACTATTAAAGAACCAGTTTTGGTTCC
R-octa-staple-13	TAAAGGTGGCAACATAGTAGAAAATAATAA
R-octa-staple-14	GATAAGTCCTGAACAACTGTTTAAAGAGAA
R-octa-staple-15	GGTAATAGTAAAATGTAAGTTTTACACTAT
R-octa-staple-16	TCAGAACCGCCACCCTCTCAGAGTATTAGC
R-octa-staple-17	AAGGGAACCGAACTGAGCAGACGGTATCAT
R-octa-staple-18	GTAAAGATTCAAAAGGCCTGAGTTGACCCT
R-octa-staple-19	AGGCGTTAAATAAGAAGACCGTGTCGCAAG
R-octa-staple-20	CAGGTCGACTCTAGAGCAAGCTTCAAGGCG
R-octa-staple-21	CAGAGCCACCACCCTCTCAGAACTCGAGAG
R-octa-staple-22	TTCACGTTGAAAATCTTGCGAATGGGATTT
R-octa-staple-23	AAGTTTTAACGGGGTCGGAGTGTAGAATGG
R-octa-staple-24	TTGCGTATTGGGCGCCCGCGGGGTGCGCTC
R-octa-staple-25	GTCACCAGAGCCATGGTGAATTATCACCAATCAGAAAAGCCT
R-octa-staple-26	GGACAGAGTTACTTTGTCGAAATCCGCGTGTATCACCGTACG
R-octa-staple-27	CAACATGATTTACGAGCATGGAATAAGTAAGACGACAATAAA
R-octa-staple-28	AACCAGACGCTACGTTAATAAAACGAACATACCACATTCAGG
R-octa-staple-29	TGACCTACTAGAAAAAGCCCCAGGCAAAGCAATTTCATCTTC
R-octa-staple-30	TGCCGGAAGGGGACTCGTAACCGTGCATTATATTTTAGTTCT
R-octa-staple-31	AGAACCCCAAATCACCATCTGCGGAATCGAATAAAAATTTTT
R-octa-staple-32	GCTCCATTGTGTACCGTAACACTGAGTTAGTTAGCGTAACCT

R-octa-staple-33	AGTACCGAATAGGAACCCAAACGGTGTAACCTCAGGAGGTTT
R-octa-staple-34	CAGTTTGAATGTTTAGTATCATATGCGTAGAATCGCCATAGC
R-octa-staple-35	AAGATTGTTTTTTAACCAAGAAACCATCGACCCAAAAACAGG
R-octa-staple-36	TCAGAGCGCCACCACATAATCAAAATCAGAACGAGTAGTATG
R-octa-staple-37	GATGGTTGGGAAGAAAAATCCACCAGAAATAATTGGGCTTGA
R-octa-staple-38	CTCCTTAACGTAGAAACCAATCAATAATTCATCGAGAACAGA
R-octa-staple-39	AGACACCTTACGCAGAACTGGCATGATTTTCTGTCCAGACAA
R-octa-staple-40	GCCAGCTAGGCGATAGCTTAGATTAAGACCTTTTTAACCTGT
R-octa-staple-41	CCGACTTATTAGGAACGCCATCAAAAATGAGTAACAACCCCA
R-octa-staple-42	GTCCAATAGCGAGAACCAGACGACGATATTCAACGCAAGGGA
R-octa-staple-43	CCAAAATACAATATGATATTCAACCGTTAGGCTATCAGGTAA
R-octa-staple-44	AACAGTACTTGAAAACATATGAGACGGGTCTTTTTTAATGGA
R-octa-staple-45	TTTCACCGCATTAAAGTCGGGAAACCTGATTTGAATTACCCA
R-octa-staple-46	GAGAATAGAGCCTTACCGTCTATCAAATGGAGCGGAATTAGA
R-octa-staple-47	ATAATTAAATTTAAAAAACTTTTTCAAACTTTTAACAACGCC
R-octa-staple-48	GCACCCAGCGTTTTTTATCCGGTATTCTAGGCGAATTATTCA
R-octa-staple-49	GGAAGCGCCCACAAACAGTTAATGCCCCGACTCCTCAAGATA
R-octa-staple-50	GTTTGCCTATTCACAGGCAGGTCAGACGCCACCACACCACCC
R-octa-staple-51	CGCGAGCTTAGTTTTTCCCAATTCTGCGCAAGTGTAAAGCCT
R-octa-staple-52	AGAAGCAACCAAGCCAAAAGAATACACTAATGCCAAAACTCC
R-octa-staple-53	ATTAAGTATAAAGCGGCAAGGCAAAGAAACTAATAGGGTACC
R-octa-staple-54	CAGTGCCTACATGGGAATTTACCGTTCCACAAGTAAGCAGAT
R-octa-staple-55	ATAAGGCGCCAAAAGTTGAGATTTAGGATAACGGACCAGTCA
R-octa-staple-56	TGCTAAACAGATGAAGAAACCACCAGAATTTAAAAAAAGGCT
R-octa-staple-57	CAGCCTTGGTTTTGTATTAAGAGGCTGACTGCCTATATCAGA
R-octa-staple-58	CGGAATAATTCAACCCAGCGCCAAAGACTTATTTTAACGCAA
R-octa-staple-59	CGCCTGAATTACCCTAATCTTGACAAGACAGACCATGAAAGA
R-octa-staple-60	ACGCGAGGCTACAACAGTACCTTTTACAAATCGCGCAGAGAA
R-octa-staple-61	CAGCGAACATTAAAAGAGAGTACCTTTACTGAATATAATGAA
R-octa-staple-62	GGACGTTTAATTTCGACGAGAAACACCACCACTAATGCAGAT
R-octa-staple-63	AAAGCGCCAAAGTTTATCTTACCGAAGCCCAATAATGAGTAA
R-octa-staple-64	GAGCTCGTTGTAAACGCCAGGGTTTTCCAAAGCAATAAAGCC
R-octa-staple-65	AATTATTGTTTTCATGCCTTTAGCGTCAGATAGCACGGAAAC
R-octa-staple-66	AAGTTTCAGACAGCCGGGATCGTCACCCTTCTGTAGCTCAAC
R-octa-staple-67	ACAAAGAAATTTAGGTAGGGCTTAATTGTATACAACGGAATC
R-octa-staple-68	AACAAAAATAACTAGGTCTGAGAGACTACGCTGAGTTTCCCT

R-octa-staple-69	CATAACCTAAATCAACAGTTCAGAAAACGTCATAAGGATAGC
R-octa-staple-70	CACGACGAATTCGTGTGGCATCAATTCTTTAGCAAAATTACG
R-octa-staple-71	CCTACCAACAGTAATTTTATCCTGAATCAAACAGCCATATGA
R-octa-staple-72	GATTATAAAGAAACGCCAGTTACAAAATTTACCAACGTCAGA
R-octa-staple-73	AGTAGATTGAAAAGAATCATGGTCATAGCCGGAAGCATAAGT
R-octa-staple-74	TAGAATCCATAAATCATTTAACAATTTCTCCCGGCTTAGGTT
R-octa-staple-75	AAAGGCCAAATATGTTAGAGCTTAATTGATTGCTCCATGAGG
R-octa-staple-76	CCAAAAGGAAAGGACAACAGTTTCAGCGAATCATCATATTCC
R-octa-staple-77	GAAATCGATAACCGGATACCGATAGTTGTATCAGCTCCAACG
R-octa-staple-78	TGAATATTATCAAAATAATGGAAGGGTTAATATTTATCCCAA
R-octa-staple-79	GAGGAAGCAGGATTCGGGTAAAATACGTAAAACACCCCCCAG
R-octa-staple-80	GGTTGATTTTCCAGCAGACAGCCCTCATTCGTCACGGGATAG
R-octa-staple-81	CAAGCCCCCACCCTTAGCCCGGAATAGGACGATCTAAAGTTT
R-octa-staple-82	TGTAGATATTACGCGGCGATCGGTGCGGGCGCCATCTTCTGG
R-octa-staple-83	CATCCTATTCAGCTAAAAGGTAAAGTAAAAAGCAAGCCGTTT
R-octa-staple-84	CAGCTCATATAAGCGTACCCCGGTTGATGTGTCGGATTCTCC
R-octa-staple-85	CATGTCACAAACGGCATTAAATGTGAGCAATTCGCGTTAAAT
R-octa-staple-86	AGCGTCACGTATAAGAATTGAGTTAAGCCCTTTTTAAGAAAG
R-octa-staple-87	TATAAAGCATCGTAACCAAGTACCGCACCGGCTGTAATATCC
R-octa-staple-88	ATAGCCCGCGAAAATAATTGTATCGGTTCGCCGACAATGAGT
R-octa-staple-89	AGACAGTTCATATAGGAGAAGCCTTTATAACATTGCCTGAGA
R-octa-staple-90	AACAGGTCCCGAAATTGCATCAAAAAGATCTTTGATCATCAG
R-octa-staple-91	ACTGCCCTTGCCCCGTTGCAGCAAGCGGCAACAGCTTTTTCT
R-octa-staple-92	TCAAAGGGAGATAGCCCTTATAAATCAAGACAACAACCATCG
R-octa-staple-93	GTAATACGCAAACATGAGAGATCTACAACTAGCTGAGGCCGG
R-octa-staple-94	GAGATAACATTAGAAGAATAACATAAAAAGGAAGGATTAGGA
R-octa-staple-95	CAGATATTACCTGAATACCAAGTTACAATCGGGAGCTATTTT
R-octa-staple-96	CATATAACTAATGAACACAACATACGAGCTGTTTCTTTGGGG
R-octa-staple-97	ATGTTTTGCTTTTGATCGGAACGAGGGTACTTTTTCTTTTGATAAGAGGTCATT
R-octa-staple-98	GGGGTGCCAGTTGAGACCATTAGATACAATTTTCACTGTGTGAAATTGTTATCC
R-octa-staple-99	CTTCGCTGGGCGCAGACGACAGTATCGGGGCACCGTCGCCATTCAGGCTGCGCA
R-octa-staple-100	TCAGAGCTGGGTAAACGACGGCCAGTGCGATCCCCGTAGTAGCATTAACATCCA

R-octa-staple-101	TTAGCGGTACAGAGCGGGAGAATTAACTGCGCTAATTTCGGAACCTATTATTCT
R-octa-staple-102	GATATTCTAAATTGAGCCGGAACGAGGCCCAACTTGGCGCATAGGCTGGCTGAC
R-octa-staple-103	TGTCGTCATAAGTACAGAACCGCCACCCATTTTCACAGTACAAACTACAACGCC
R-octa-staple-104	CGATTATAAGCGGAGACTTCAAATATCGCGGAAGCCTACGAAGGCACCAACCTA
R-octa-staple-105	AACATGTACGCGAGTGGTTTGAAATACCTAAACACATTCTTACCAGTATAAAGC
R-octa-staple-106	GTCTGGATTTTGCGTTTTAAATGCAATGGTGAGAAATAAATTAATGCCGGAGAG
R-octa-staple-107	GCCTTGAATCTTTTCCGGAACCGCCTCCCAGAGCCCAGAGCCGCCGCCAGCATT
R-octa-staple-108	CGCTGGTGCTTTCCTGAATCGGCCAACGAGGGTGGTGATTGCCCTTCACCGCCT
R-octa-staple-109	TGATTATCAACTTTACAACTAAAGGAATCCAAAAAGTTTGAGTAACATTATCAT
R-octa-staple-110	ACATAACTTGCCCTAACTTTAATCATTGCATTATAACAACATTATTACAGGTAG
R-octa-staple-111	GTAGCGCCATTAAATTGGGAATTAGAGCGCAAGGCGCACCGTAATCAGTAGCGA
R-octa-staple-112	TTATTTTTACCGACAATGCAGAACGCGCGAAAAATCTTTCCTTATCATTCCAAG
R-octa-staple-113	TTTCAATAGAAGGCAGCGAACCTCCCGATTAGTTGAAACAATAACGGATTCGCC
R-octa-staple-114	GGGCGACCCCAAAAGTATGTTAGCAAACTAAAAGAGTCACAATCAATAGAAAAT
R-octa-staple-115	AGCCGAAAGTCTCTCTTTTGATGATACAAGTGCCTTAAGAGCAAGAAACAATGA
R-octa-staple-116	GTGGGAAATCATATAAATATTTAAATTGAATTTTTGTCTGGCCTTCCTGTAGCC
R-octa-staple-117	CCCACGCGCAAAATGGTTGAGTGTTGTTCGTGGACTTGCTTTCGAGGTGAATTT
R-octa-staple-118	ATGACCACTCGTTTGGCTTTTGCAAAAGTTAGACTATATTCATTGAATCCCCCT

R-octa-staple-119	TCCAAATCTTCTGAATTATTTGCACGTAGGTTTAACGCTAACGAGC GTCTTTCC
R-octa-staple-120	GGGTTATTTAATTACAATATATGTGAGTAATTAATAAGAGTCAATA GTGAATTT

Supplementary Table 2. Sticky ends (SE) of R-octa frames. All the sequences are present from 5'-3' (left to right).

R-octa-SE-A-1	CAAATGCTTTAAAAAATCAGGTCTTTAAGAGCAGCCAGAGGGTTTTT TTTTTTTTTTTTTTTTTATCCGTTA
R-octa-SE-A-2	AAAGATTCATCAGGAATTACGAGGCATGCTCATCCTTATGCGTTTTT TTTTTTTTTTTTTTTTTATCCGTTA
R-octa-SE-A-3	CTTCATCAAGAGAAATCAACGTAACAGAGATTTGTCAATCATTTTTT TTTTTTTTTTTTTTTTTATCCGTTA
R-octa-SE-A-4	AAACGAAAGAGGGCGAAACAAAGTACTGACTATATTCGAGCTTTTT TTTTTTTTTTTTTTTTTTATCCGTTA
R-octa-SE-A-5	GGTAGCTATTTTAGAGAATCGATGAAAACATTAAATGTGTAGTTTTT TTTTTTTTTTTTTTTTTATCCGTTA
R-octa-SE-A-6	ATAAATCATACATAAATCGGTTGTACTGTGCTGGCATGCCTGTTTTT TTTTTTTTTTTTTTTTTATCCGTTA
R-octa-SE-A-7	ACTGTTGGGAAGCAGCTGGCGAAAGGATAGGTCAAGATCGCATTTT TTTTTTTTTTTTTTTTTTATCCGTTA
R-octa-SE-A-8	AGCTTTCATCAACGGATTGACCGTAAAATCGTATAATATTTTTTTTT TTTTTTTTTTTTTTTTATCCGTTA
R-octa-SE-A-9	GACAGGAGGTTGAAACAAATAAATCCGCCCCCTCCGCCACCCTTTTT TTTTTTTTTTTTTTTTTATCCGTTA
R-octa-SE-A-10	CAGAATCAAGTTTCGGCATTTTCGGTTAAATATATCACCAGTTTTTTT TTTTTTTTTTTTTTTTATCCGTTA
R-octa-SE-A-11	TCATATGGTTTACGATTGAGGGAGGGAAACGCAATACATACATTTTT TTTTTTTTTTTTTTTTTATCCGTTA
R-octa-SE-A-12	AATAGCAATAGCACCAGAAGGAAACCTAAAGCCACTGGTAATTTTT TTTTTTTTTTTTTTTTTTATCCGTTA
R-octa-SE-A-13	TGTAGCATTCCAACGTTAGTAAATGAAGTGCCGCGCCACCCTTTTTT TTTTTTTTTTTTTTTTTATCCGTTA
R-octa-SE-A-14	GAAACATGAAAGCTCAGTACCAGGCGAAAAATGCTGAACAAATTTT TTTTTTTTTTTTTTTTTTATCCGTTA
R-octa-SE-A-15	AGAGCCTAATTTGATTTTTTGTTTAAATCCTGAAATAAAGAATTTTTT TTTTTTTTTTTTTTTTATCCGTTA
R-octa-SE-A-16	TTTGCGGAACAATGGCAATTCATCAATCTGTATAATAATTTTTTTTT TTTTTTTTTTTTTTTTATCCGTTA
R-octa-SE-A-17	TTTGCGGATGGCCAACTAAAGTACGGGCTTGCAGCTACAGAGTTTTT TTTTTTTTTTTTTTTTTATCCGTTA

R-octa-SE-A-18	CTTAAACAGCTTATATATTCGGTCGCTTGATGGGGAACAAGATTTTT TTTTTTTTTTTTTTTTTATCCGTTA
R-octa-SE-A-19	GGCCCTGAGAGAAGCAGGCGAAAATCATTGCGTAGAGGCGGTTTTT TTTTTTTTTTTTTTTTTTATCCGTTA
R-octa-SE-A-20	GCTCACAATTCCGTGAGCTAACTCACTGGAAGTAATGGTCAATTTTT TTTTTTTTTTTTTTTTTATCCGTTA
R-octa-SE-A-21	CAACGCTCAACAGCAGAGGCATTTTCAATCCAATGATAAATATTTTT TTTTTTTTTTTTTTTTTATCCGTTA
R-octa-SE-A-22	ATCAAAATCATATATGTAAATGCTGAACAAACACTTGCTTCTTTTTT TTTTTTTTTTTTTTTTTATCCGTTA
R-octa-SE-A-23	TGATTGCTTTGAGCAAAAGAAGATGAAATAGCAGAGGTTTTGTTTTT TTTTTTTTTTTTTTTTTATCCGTTA
R-octa-SE-A-24	AACGGGTATTAAGGAATCATTACCGCCAGTAATTCAACAATATTTTT TTTTTTTTTTTTTTTTTATCCGTTA
R-octa-SE-B-1	CAAATGCTTTAAAAAATCAGGTCTTTAAGAGCAGCCAGAGGGTTTTT TTTTTTTTTTTTTTTTTTAACGGAT
R-octa-SE-B-2	AAAGATTCATCAGGAATTACGAGGCATGCTCATCCTTATGCGTTTTT TTTTTTTTTTTTTTTTTTAACGGAT
R-octa-SE-B-3	CTTCATCAAGAGAAATCAACGTAACAGAGATTTGTCAATCATTTTTT TTTTTTTTTTTTTTTTTTAACGGAT
R-octa-SE-B-4	AAACGAAAGAGGGCGAAACAAAGTACTGACTATATTCGAGCTTTTT TTTTTTTTTTTTTTTTTTTAACGGAT
R-octa-SE-B-5	GGTAGCTATTTTAGAGAATCGATGAAAACATTAAATGTGTAGTTTTT TTTTTTTTTTTTTTTTTTAACGGAT
R-octa-SE-B-6	ATAAATCATACATAAATCGGTTGTACTGTGCTGGCATGCCTGTTTTT TTTTTTTTTTTTTTTTTTAACGGAT
R-octa-SE-B-7	ACTGTTGGGAAGCAGCTGGCGAAAGGATAGGTCAAGATCGCATTTT TTTTTTTTTTTTTTTTTTTAACGGAT
R-octa-SE-B-8	AGCTTTCATCAACGGATTGACCGTAAAATCGTATAATATTTTTTTTT TTTTTTTTTTTTTTTTTAACGGAT
R-octa-SE-B-9	GACAGGAGGTTGAAACAAATAAATCCGCCCCCTCCGCCACCCTTTTT TTTTTTTTTTTTTTTTTTAACGGAT
R-octa-SE-B-10	CAGAATCAAGTTTCGGCATTTTCGGTTAAATATATCACCAGTTTTTTT TTTTTTTTTTTTTTTTTAACGGAT
R-octa-SE-B-11	TCATATGGTTTACGATTGAGGGAGGGAAACGCAATACATACATTTTT TTTTTTTTTTTTTTTTTTAACGGAT

R-octa-SE-B-12	AATAGCAATAGCACCAGAAGGAAACCTAAAGCCACTGGTAATTTTT<br>TTTTTTTTTTTTTTTTTTAACGGAT
R-octa-SE-B-13	TGTAGCATTCCAACGTTAGTAAATGAAGTGCCGCGCCACCCTTTTTT<br>TTTTTTTTTTTTTTTTTAACGGAT
R-octa-SE-B-14	GAAACATGAAAGCTCAGTACCAGGCGAAAAATGCTGAACAAATTTT<br>TTTTTTTTTTTTTTTTTTAACGGAT
R-octa-SE-B-15	AGAGCCTAATTTGATTTTTTGTTTAAATCCTGAAATAAAGAATTTTTT<br>TTTTTTTTTTTTTTTTAACGGAT
R-octa-SE-B-16	TTTGCGGAACAATGGCAATTCATCAATCTGTATAATAATTTTTTTTT<br>TTTTTTTTTTTTTTTTAACGGAT
R-octa-SE-B-17	TTTGCGGATGGCCAACTAAAGTACGGGCTTGCAGCTACAGAGTTTTT<br>TTTTTTTTTTTTTTTTTAACGGAT
R-octa-SE-B-18	CTTAAACAGCTTATATATTCGGTCGCTTGATGGGGAACAAGATTTTT<br>TTTTTTTTTTTTTTTTTAACGGAT
R-octa-SE-B-19	GGCCCTGAGAGAAGCAGGCGAAAATCATTGCGTAGAGGCGGTTTTT<br>TTTTTTTTTTTTTTTTTTAACGGAT
R-octa-SE-B-20	GCTCACAATTCCGTGAGCTAACTCACTGGAAGTAATGGTCAATTTTT<br>TTTTTTTTTTTTTTTTTAACGGAT
R-octa-SE-B-21	CAACGCTCAACAGCAGAGGCATTTTCAATCCAATGATAAATATTTTT<br>TTTTTTTTTTTTTTTTTAACGGAT
R-octa-SE-B-22	ATCAAAATCATATATGTAAATGCTGAACAAACACTTGCTTCTTTTTT<br>TTTTTTTTTTTTTTTTTAACGGAT
R-octa-SE-B-23	TGATTGCTTTGAGCAAAAGAAGATGAAATAGCAGAGGTTTTGTTTTT<br>TTTTTTTTTTTTTTTTTAACGGAT
R-octa-SE-B-24	AACGGGTATTAAGGAATCATTACCGCCAGTAATTCAACAATATTTTT<br>TTTTTTTTTTTTTTTTTAACGGAT

Supplementary Table 3. Staples strands of E-octa frames. All the sequences are present from 5'-3' (left to right).

E-octa-staple-1	CTCGTTTACCAGACGACAACACTAAAGATT
E-octa-staple-2	AAAAGGGACATTCTGGTCACACGTTGCAAC
E-octa-staple-3	GCCACTACGAAGGCACGGGTAAAGCGAAAG
E-octa-staple-4	TTGGGGCGCGAGCTGATTAGCTATTCCATA
E-octa-staple-5	TTCAAATATATTTTAGAACGCGACCTCCGG
E-octa-staple-6	CAATATAATCCTGATTGATGATGATTTTAA
E-octa-staple-7	CAGACTGTAGCGCGTTAGTTTGCCCAGTAG
E-octa-staple-8	GTCCACTATTAAAGAACCAGTTTTGGTTCC
E-octa-staple-9	GAATAATAATTTTTTCCAACTAATAACGAT
E-octa-staple-10	GGCCGATTAAAGGGATCGGGAGCCCGCCGC
E-octa-staple-11	GCCTCTTCGCTATTACAGGGCGAGCACCGC
E-octa-staple-12	AAGCCAGAATGGAAAGAAATAAACAGAGCC
E-octa-staple-13	CCAGACGACGACAATAGGTAAAGCTCAACA
E-octa-staple-14	TACCCAAATCAACGTAAGAACCGACGGTCA
E-octa-staple-15	TTGCGCTCACTGCCCGACTCACACATGGTC
E-octa-staple-16	AATTACATTTAACAATTCAAGAAATTGCTT
E-octa-staple-17	GCCATCAAAAATAATTTTTAACCTAATCAG
E-octa-staple-18	AGTCAAATCACCATCAGAGAAAGTTTCAAC
E-octa-staple-19	AACAAAGTCAGAGGGTTTAACTGTTATCCC
E-octa-staple-20	TTATTTTGTCACAATCACACCACACGCAGT
E-octa-staple-21	ATCTGGTCAGTTGGCACAAACCCAGTATTA
E-octa-staple-22	TAAGTATAGCCCGGAAGTCGAGAAAACATG
E-octa-staple-23	AGCGAACCAGACCGGATTAATTCGTCAGAA
E-octa-staple-24	GACTTGCGGGAGGTTTTTTAGCTTACCGC
E-octa-staple-25	ACAGCATGCTCCATAGATTTGTATCATCCCCAGCGAAACGAA
E-octa-staple-26	GAAATCGGCCCCCTACGGGGTCAGTGCCCTTTTGATCCAACG
E-octa-staple-27	AACGGGTCCTGAACAAGAAAAATAATATCTTATCATTCCAAG
E-octa-staple-28	AAAAGCCCTCAGGACGTTGGTGTAGATGGGGAACAGGCCTTC
E-octa-staple-29	ATTAAATCAGCTTTCATCAACATTAAATTTGTTAAAATTCGC
E-octa-staple-30	TACATTTAATAGTACATCCAATAAATCAAAGCTAACCAAAAA
E-octa-staple-31	GCCCAATTTTGCCATAACGAGCGTCTTTGCACCCATTAAATC
E-octa-staple-32	ATAGCGAAATTACGTAGGAATACCACATCAGTACAGTACCGT
E-octa-staple-33	GTTGGGATGAAAGAGGACAGATGAACGGAGTAGATCATTAGA
E-octa-staple-34	CTTTTTCAAAGAATACTCATCTTTGACCGCCTGATGAAATCC

E-octa-staple-35	AAGCCTGCGTGCCAGCTGCATTAATGAAAAGCATAAAGTGTA
E-octa-staple-36	TCAGTGATCATCAAGAACTGACCAACTTAGAAAAATCTACGT
E-octa-staple-37	TAACAGTACCCTGTAGCCTCAGAGCATATACAGGCGCATCAA
E-octa-staple-38	CTAATGCGAATATAAGAATCGCCATATTTACCGCACTCATCG
E-octa-staple-39	ATAGCTGTTGCCCCCGGGCAACAGCTGAATTGGGCGTCGGGA
E-octa-staple-40	GCCGCCATGTAGCGGGAAGGGAAGAAAGAGAGCTTTCTGAAT
E-octa-staple-41	GGAATTAAATGGAACTACCATATCAAAACGTCAGAGTAACAG
E-octa-staple-42	TCTGAATTCATCATTTATCATTTTGCGGTAATACATGAATGG
E-octa-staple-43	AGAGGCAATGAGGAAGGGTAGCAACGGCAGGTGTCAAATTCC
E-octa-staple-44	CGTTCTATAGGTAATTTTAGAACCCTCAAGGATGAACGGTAA
E-octa-staple-45	TTCTACTCGCAAATCAATTCTGCGAACGTGTTGTAATCGGTA
E-octa-staple-46	AGGAAAACCAGCAGACTGATAGCCCTAAACAATATAGATAGA
E-octa-staple-47	GAGCCGGTCGTAAGAAAGCGGCCAACGCTGATCGTGCTCAAG
E-octa-staple-48	AAACAGGAGATAACCCACAAGAATTGAGAGAGAATAACATAA
E-octa-staple-49	GTGCATCACAACCCGTCGGATTCTCCGTGGCGCATCGTAACC
E-octa-staple-50	CTAAAGTAGGCCGCACAATGACAACAACTGAATTTAAATCTC
E-octa-staple-51	AATCCAACAAAAGAAAGTAAGCAGATAGAATAGCACGCTAAT
E-octa-staple-52	TAACGTGAGAATCCGTGAGTGAATAACCACATAGCGATAGCT
E-octa-staple-53	GGATTATTGACCTGAATACGTGGCACAGAACATCGTACCGAA
E-octa-staple-54	GTACGCCCTTTCCTTACAGGGCGCGTACAGAGTCAATAGTGA
E-octa-staple-55	ATCATTTCGAAAGGAGCGGGAATAGCCCGCGAAAAAGCGTCA
E-octa-staple-56	TTAATTGATATAATGCTGTGGAAGCCCGATTAGAGAAGGCGA
E-octa-staple-57	ATCATAAACGAACTATGCGATTTTAAGAATGGTTTTGCTCAT
E-octa-staple-58	AAGCATCGAGGAAGATATCTTTAGGAGCGAAGTATAAACAAT
E-octa-staple-59	AAAGTATTCAAAAAGTCATAAATATTCAAAATGTTATCACCG
E-octa-staple-60	GCAAGGAACTAGCAGAGAGTCTGGAGCATTTTTGAATTCAAC
E-octa-staple-61	ATCAGAGGAAGCGCACGATTTTTTGTTTACGCAATAATAACG
E-octa-staple-62	CACCATTACCACCCGCCTCCCTCAGAGCTAATCAAGCATTTT
E-octa-staple-63	TTTGCTAAAAGCGTTTATTTTGTATCGGATACCATATGAAAT
E-octa-staple-64	TAATGTGGCTGATAAATTATGCTATTTTCCGCAATGCCTGAG
E-octa-staple-65	TAGATTAAATATATTGAGAAGTGTTTTTTGGACGAGCACGTA
E-octa-staple-66	CGAGGAAAACGTCAAAAATGAAAATAGCTACAGAGCTAAAGA
E-octa-staple-67	ATGTTAGTTATACACCGGAATCATAATTGACCGTGAATTCAT
E-octa-staple-68	CAAAAGGGAGGCTTGCCACCCTCAGAACAACCCATAACTACA
E-octa-staple-69	AAGATTAGTATTCTAAATCAGATATAGATATATTTTAAATAG
E-octa-staple-70	AACCGATTTTATCAGCTTGCTTTCGAGGCATCGCCCACGCAT

E-octa-staple-71	CAAAAAACGGAGTGTCTTTCCAGACGTTCTGAGGCTTGCAGG
E-octa-staple-72	TCGGTCGAGTAAATGAATTTTCTGTATGGTCACCACGATAGC
E-octa-staple-73	CAGACCAAAATTAAGTAGCCACCAGAACGGTTGACTTAGTAC
E-octa-staple-74	CAGAGGCAAAGAACGGGTTTAGATAAGTATACCAGAAACCTA
E-octa-staple-75	GTTTCAGAAGGCTCCAAAAGGAGCCTTTAACAACTTTCAACA
E-octa-staple-76	AACCTGTGGGTGCCTGTGAAATTGTTATCAGCAAGCGGTCCA
E-octa-staple-77	CAGAAGGAATAAGAGCAAGAAACAATGACCGAACAAAGTTAC
E-octa-staple-78	TTTACAGTTAAAACACACTAAGCCCAATAAGAGGAGCTTTAC
E-octa-staple-79	GTAGGGCGCAAGCCATCGGCTGTCTTTCCCCATCCTGTTCAG
E-octa-staple-80	AAGTTTGTACATCGATTTTCAGGTTTAATTATTTGTTATACT
E-octa-staple-81	ACTTGCCATAATCAACAGTACATAAATCAGATTTCTATTCAC
E-octa-staple-82	TAATATTGTCTAAAGTTATGAGCGAGTATGATGAAAGCAACC
E-octa-staple-83	ATGGTTTACATATAAGAAAATACATACAAACTGTTTAGTATC
E-octa-staple-84	CGGTCATTAATCAGGCAAGGCCGGAAACGGAACCGCTCAGAT
E-octa-staple-85	ACCCTTCTTACATTTGGAAATACCTACAATAAAAACCATTAC
E-octa-staple-86	CAGTTCAGAGAAGGATTAGTTTCGTCACTCAACTAATAACGC
E-octa-staple-87	GGTCAGGAAAGACTATCAAAAAGATTAACACCTGCAGGTCGA
E-octa-staple-88	TGAGCAAAATGGAAGTGAGGCCACCGAGTTAGTAACTATCGG
E-octa-staple-89	AGAGTTGCCGCTCACAATTCCACACAACTTTTGACCTGAAAT
E-octa-staple-90	AGCACCGAGCCCCCTTGCCATCTTTTCACGCCACCCCACCCT
E-octa-staple-91	CTTTTTTAAGAAGACAAAATCGCGCAGAACTCAAATAACATC
E-octa-staple-92	AGGAATTACCTTGCAGTGCCACGCTGAGACTTTACTAGACGT
E-octa-staple-93	CCAGTCAGAGTAGTAAATTGGGCTTGAGACTGGCTCATTATA
E-octa-staple-94	GCAAAGCGGATCCCACGACGGCCAGTGCGGGTAACTCCAACA
E-octa-staple-95	AACACTGCAGAACCTTGCAAAAGAAGTTTAGATACATGCAAA
E-octa-staple-96	CCACCCTAGGATTAGCGGGGTTTTGCTCGAGGTTTAGGGGGT
E-octa-staple-97	GCTGAGATATGGTTGCTTTAGTAGAAGAGGCGAATAATTACC
E-octa-staple-98	AGTTTGAAAGCAAATATTTAAATTGTAAAGCCAGCAAATCTA
E-octa-staple-99	GCTTAATAAAATCATAGAATCCTTGAAATTGCTTCAGGAACG
E-octa-staple-100	TCAAAGGGAGATAGCCCTTATAAATCAAAGGCCCGTATAAAC
E-octa-staple-101	TTCTGGTATGCAACAGCTTAATTGCTGACTCCTTTGGCGAAA
E-octa-staple-102	AATAAGAAGAACGCGCCTGTTTATCAACATTTTCGAGCCAGT
E-octa-staple-103	CTTAGGTGAGCCATGACGGAAATTATTCGCGACATCATCTTC
E-octa-staple-104	AAATACCACTAGAAAAAGCTGCTGATGCAATTTAACCAAAGA
E-octa-staple-105	TGAATACGGTAATACAATACTTCTTTGATAAAAGAGAATTAC
E-octa-staple-106	TGACCTAAAATCCATATAACTATATGTATATTATCACCGTCA

E-octa-staple-107	CTGTAGCTTTTGTTCAGGAAGATTGTATGGGGACGACGACAG
E-octa-staple-108	GGGGGATCAGGCTGACCAGGCAAAGCGCGAAGCTCAACATGT
E-octa-staple-109	ACACCGCCTCGTATCATTTGAGGATTTAACTAACAAGTTGAA
E-octa-staple-110	CTGGCCCGCGGGGAGAGGCGGTTTGCGTTTGCCCTTCACCGC
E-octa-staple-111	TACTCAGAGTACCACTGAGACTCCTCAAGAAAACGAGAATGA
E-octa-staple-112	AATAGTATTGAATCCCCCTCAAATGCTTTTGCCAGAGTACCG
E-octa-staple-113	CAAAAGGATTAAAGGTGAAAAGGTGGCAACCAGCGTGGTTTG
E-octa-staple-114	TTACCGTTTGGCCTCAGGAGGTTGAGGCAAGCGCTAGGGCGC
E-octa-staple-115	TACATGGTTGAGTAACAGTGTCAGACGATCCAGTAACCGTCT
E-octa-staple-116	TTAAGTTCAAGCTTGCATGTTCGCCATTGTGCTGCAGTACCT
E-octa-staple-117	TTATCCGGTATGCCGGAGAGGGTAGCTAAACAAGAGAATCGC
E-octa-staple-118	CTGACCTATAAGGCTTGCCCTGACGAGAGGCGCATAGGCTGG
E-octa-staple-119	AACCAAGTAACAACGCCAACATGTAATTAACAAAGAAGGAGC
E-octa-staple-120	CATCAGTTAGCATTGCAAGCCCAATAGGCGCCACCAACCAAA
E-octa-staple-121	AGTTAATGCAAAATGGTTGAGTGTTGTTCGTGGACTGATACAGGAG TGTACTGG
E-octa-staple-122	GAATACCATAAGAAATTAGACGGGAGAAAATTGAGATAGCTATCTT ACCGAAGC
E-octa-staple-123	GCGACCTCGGAACGAGTTTCCATTAAACCAACCTAATTATACCAAG CGCGAAAC
E-octa-staple-124	TATCGGCCCAAAAAAAATCAGCTCATTTCGCGTCTAACGGCGGATT GACCGTAA
E-octa-staple-125	TCGTAAATAAAAATAGATTCAAAAGGGTATATGATGAGATCTACAA AGGCTATC
E-octa-staple-126	CATTATGTGATTCCGGTCAATAACCTGTAAAGGTGAAGGCAAAGAA TTAGCAAA
E-octa-staple-127	AGAACAATTAATTGAAGTACCGACAAAAAACAACATAATTTACGAG CATGTAGA
E-octa-staple-128	ATATGCGCAAACGTAAAGAAACGCAAAGAATAGAATGATAAATAA GGCGTTAAA
E-octa-staple-129	CCGACTTTGGGTTAATCGCAAGACAAAGTTAATTTTCAACCGATTG AGGGAGGG
E-octa-staple-130	ACGCCTGTGAGATTAGGCATAGTAAGAGCGATAAACTCAGAGCCAC CACCCTCA
E-octa-staple-131	CGCTGGTTTTCCTGTAATGAGTGAGCTACTTTCCAGCCAGGGTGGTT TTTCTTT

E-octa-staple-132	CGAACCAACGCTCAGGCAGATTCACCAGCCAACAGTTTTGAATGGCTATTAGTC
E-octa-staple-133	TAATAAAGGGAACCGAGTAATCTTGACAACAAAGCAATTTCAACTTTAATCATT
E-octa-staple-134	CCTTGCTCAAGTTATGATGAAACAAACATTCATTTGTCTGTCCATCACGCAAAT
E-octa-staple-135	GAGTTAATTTGTCGAGAATAGAAAGGAAACGTTGACTTAAACAGCTTGATACCG
E-octa-staple-136	TGGCAAGGCATTGATGATATTCACAAACCGCAGTCGACGGGGAAAGCCGGCGAA
E-octa-staple-137	TCGACAACTGCAACTGAACCTCAAATATAATCAACACTAATAGATTAGAGCCGT
E-octa-staple-138	AGCCTAAAGCAAGCAAGAACGCGAGGCGTGAAGCCGCTACAATTTTATCCTGAA
E-octa-staple-139	CAGAGCCACCATTATAGCGACAGAATCATTCATCGAATCACCGGAACCAGAGCC
E-octa-staple-140	TACCTTTAGTAACAATTCCTGATTATCAGTTTGGACACGTAAAACAGAAATAAA
E-octa-staple-141	CTCTAGAGGATTGCTCAAATATCGCGTTAGCAAACGCCAGGGTTTTCCCAGTCA
E-octa-staple-142	TTTAAATGCCGGAACGCAACTGTTGGGAGCCAGCTTGATAAGAGGTCATTTTTG
E-octa-staple-143	ATTTATCGCGCCGCCGTTAGAATCAGAGTTTAGACTGTAAATCGTCGCTATTAA
E-octa-staple-144	CCATAAATAAGAGGGGCGGATAAGTGCCTAGGTGTTAGACTGGATAGCGTCCAA

Supplementary Table 4. Sticky ends (SE) of E-octa frames. All the sequences are present from 5’-3’ (left to right).

E-octa-SE-A-1	AAAGTACAACGGGTTACTTAGCCGGACTCAGCAATACGTAATTTTTTT TTTTTTTTTTTTTTTTATCCGTTA
E-octa-SE-A-2	ATAGTTGCGCCGTTTTGCGGGATCGTGTTAGCGAGGAATTGCTTTTTTT TTTTTTTTTTTTTTTATCCGTTA
E-octa-SE-A-3	GTGAATTACCTTAACGGAACAACATTGGCGCAGGATATTCATTTTTTT TTTTTTTTTTTTTTTTATCCGTTA
E-octa-SE-A-4	TTTTCAGGGATACCACAGACAGCCCTCAGGTAGATCATAACCTTTTTT TTTTTTTTTTTTTTTTATCCGTTA
E-octa-SE-A-5	AGGTCATTGCCTTGTCAATCATATGTGCCTTTAGCCGGAGACTTTTTTT TTTTTTTTTTTTTTTCATCAAGT
E-octa-SE-A-6	TGGGATAGGTCAAGATCGCACTCCAGCGGTTGAAATAGGAACTTTTTT TTTTTTTTTTTTTTTTCATCAAGT
E-octa-SE-A-7	ATTAAGCAATAAAATACTTTTGCGGGAGTTTCATATTTTCATTTTTTTT TTTTTTTTTTTTTTTCATCAAGT
E-octa-SE-A-8	CGGATGGCTTAGTAAAGTACGGTGTCCTTTCCGTCGGTGCGGTTTTTTT TTTTTTTTTTTTTTTCATCAAGT
E-octa-SE-A-9	TCACCAGTGAGAAGCAGGCGAAAATCTCGTAATTTAATTGCGTTTTTT TTTTTTTTTTTTTTTTCATCAAGT
E-octa-SE-A-10	TAATAAGTTTTAGCCTATTTCGGAACTTGATGGGGAACAAGATTTTTT TTTTTTTTTTTTTTTTCATCAAGT
E-octa-SE-A-11	CGACGTTGTAAACGGGTACCGAGCTCTATTATAGAGCTTCAATTTTTT TTTTTTTTTTTTTTTTCATCAAGT
E-octa-SE-A-12	TACTGCGGAATCTCAGGTCTTTACCCTATTCTGGGGTTGATATTTTTTT TTTTTTTTTTTTTTTCATCAAGT
E-octa-SE-A-13	TTAATTTTCCCTTAGGTCTGAGAGACACCACACTAAACAGGATTTTTT TTTTTTTTTTTTTTTTCATCAAGT
E-octa-SE-A-14	ACCACCGGAACCTCAGAGCCGCCACCAAAATCACTTTAGCGTTTTTTT TTTTTTTTTTTTTTTTCATCAAGT
E-octa-SE-A-15	AAGGTAAATATTTTGGGAATTAGAGCTTTTTAAGAAAACTTTTTTTTTT TTTTTTTTTTTTTTTCATCAAGT
E-octa-SE-A-16	CGTGGCGAGAAAGTCACGCTGCGCGTCCACCACTCCTCATTATTTTTT TTTTTTTTTTTTTTTTCATCAAGT
E-octa-SE-A-17	CCTTTTTAAGAAACTGGCATGATTAAATATTATAACACCCTGTTTTTTT TTTTTTTTTTTTTTTCATCAAGT

E-octa-SE-A-18	TCTTACCAACGCGTTACAAAATAAACGGAATCAGAACCTCCCTTTTTT<br>TTTTTTTTTTTTTTTTCATCAAGT
E-octa-SE-A-19	AACCAATCAATAGTTTTTATTTTCATGCCAACGTAATTCTGTTTTTTT<br>TTTTTTTTTTTTTTTCATCAAGT
E-octa-SE-A-20	TAAGAATAAACAAATTCTTACCAGTACCTTATTGGAATAAGTTTTTT<br>TTTTTTTTTTTTTTTTCATCAAGT
E-octa-SE-A-21	CAATAGATAATATAAATCCTTTGCCCGGCGGTCTCAATCAATTTTTTT<br>TTTTTTTTTTTTTTTATCCGTTA
E-octa-SE-A-22	TTTAATGCGCGAAAGATAAAACAGAGCCAGCCAACCAGTAATTTTTT<br>TTTTTTTTTTTTTTTTATCCGTTA
E-octa-SE-A-23	TAACCGTTGTAGTCCAGAACAATATTTCGCCTGAACAAAATTTTTTT<br>TTTTTTTTTTTTTTTATCCGTTA
E-octa-SE-A-24	GAAATTGCGTAGGGAGAAACAATAACGTTATTAGCAATTCATTTTTT<br>TTTTTTTTTTTTTTTTATCCGTTA
E-octa-SE-B-1	AAAGTACAACGGGTTACTTAGCCGGACTCAGCAATACGTAATTTTTT<br>TTTTTTTTTTTTTTTTAACGGAT
E-octa-SE-B-2	ATAGTTGCGCCGTTTTGCGGGATCGTGTTAGCGAGGAATTGCTTTTTTT<br>TTTTTTTTTTTTTTTAACGGAT
E-octa-SE-B-3	GTGAATTACCTTAACGGAACAACATTGGCGCAGGATATTCATTTTTT<br>TTTTTTTTTTTTTTTTAACGGAT
E-octa-SE-B-4	TTTTCAGGGATACCACAGACAGCCCTCAGGTAGATCATAACCTTTTTT<br>TTTTTTTTTTTTTTTTAACGGAT
E-octa-SE-B-5	AGGTCATTGCCTTGTCAATCATATGTGCCTTTAGCCGGAGACTTTTTTT<br>TTTTTTTTTTTTTTTACTTGATG
E-octa-SE-B-6	TGGGATAGGTCAAGATCGCACTCCAGCGGTTGAAATAGGAACTTTTTT<br>TTTTTTTTTTTTTTTTACTTGATG
E-octa-SE-B-7	ATTAAGCAATAAAATACTTTTGCGGGAGTTTCATATTTTCATTTTTTT<br>TTTTTTTTTTTTTTTACTTGATG
E-octa-SE-B-8	CGGATGGCTTAGTAAAGTACGGTGTCCTTTCCGTCGGTGCGGTTTTTTT<br>TTTTTTTTTTTTTTTACTTGATG
E-octa-SE-B-9	TCACCAGTGAGAAGCAGGCGAAAATCTCGTAATTTAATTGCGTTTTTT<br>TTTTTTTTTTTTTTTTACTTGATG
E-octa-SE-B-10	TAATAAGTTTTAGCCTATTTCGGAACTTGATGGGGAACAAGATTTTTT<br>TTTTTTTTTTTTTTTTACTTGATG
E-octa-SE-B-11	CGACGTTGTAAACGGGTACCGAGCTCTATTATAGAGCTTCAATTTTTT<br>TTTTTTTTTTTTTTTTACTTGATG

E-octa-SE-B-12	TACTGCGGAATCTCAGGTCTTTACCCTATTCTGGGGTTGATATTTTTTT TTTTTTTTTTTTTTTACTTGATG
E-octa-SE-B-13	TTAATTTTCCCTTAGGTCTGAGAGACACCACACTAAACAGGATTTTTT TTTTTTTTTTTTTTTTACTTGATG
E-octa-SE-B-14	ACCACCGGAACCTCAGAGCCGCCACCAAAATCACTTTAGCGTTTTTTT TTTTTTTTTTTTTTTTACTTGATG
E-octa-SE-B-15	AAGGTAAATATTTTGGGAATTAGAGCTTTTTAAGAAAACTTTTTTTTTT TTTTTTTTTTTTTTTACTTGATG
E-octa-SE-B-16	CGTGGCGAGAAAGTCACGCTGCGCGTCCACCACTCCTCATTATTTTTT TTTTTTTTTTTTTTTTACTTGATG
E-octa-SE-B-17	CCTTTTTAAGAAACTGGCATGATTAAATATTATAACACCCTGTTTTTTT TTTTTTTTTTTTTTTACTTGATG
E-octa-SE-B-18	TCTTACCAACGCGTTACAAAATAAACGGAATCAGAACCTCCCTTTTTT TTTTTTTTTTTTTTTTACTTGATG
E-octa-SE-B-19	AACCAATCAATAGTTTTTATTTTCATGCCAACGTAATTCTGTTTTTTTT TTTTTTTTTTTTTTTACTTGATG
E-octa-SE-B-20	TAAGAATAAACAAATTCTTACCAGTACCTTATTGGAATAAGTTTTTTT TTTTTTTTTTTTTTTTACTTGATG
E-octa-SE-B-21	CAATAGATAATATAAATCCTTTGCCCGGCGGTCTCAATCAATTTTTTTT TTTTTTTTTTTTTTTTAACGGAT
E-octa-SE-B-22	TTTAATGCGCGAAAGATAAAACAGAGCCAGCCAACCAGTAATTTTTTT TTTTTTTTTTTTTTTTTAACGGAT
E-octa-SE-B-23	TAACCGTTGTAGTCCAGAACAATATTTCGCCTGAACAAAATTTTTTTTT TTTTTTTTTTTTTTTTAACGGAT
E-octa-SE-B-24	GAAATTGCGTAGGGAGAAACAATAACGTTATTAGCAATTCATTTTTTT TTTTTTTTTTTTTTTTTAACGGAT

Supplementary Figures

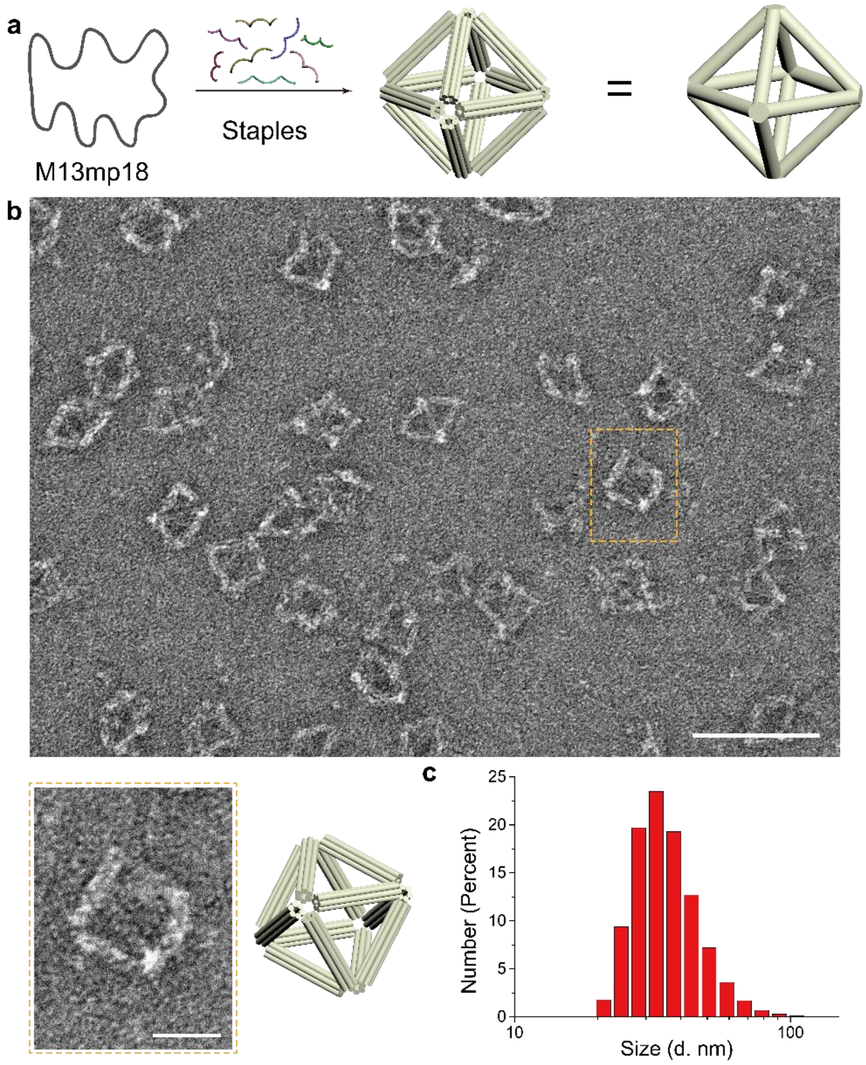

Supplementary Figure 1. Schematic illustration of the assembly process for R-octa DOFs and corresponding characterizations. a, Assembly process of R-octa DOFs, each bundle of a DOF is composed of a six-helix-bundle (6HB). For easier illustration, we represent the 6HB by a thick bundle. **b,** TEM image of negative stained R-octa DOFs. A representative individual R-octa DOF is enclosed by yellow dashed rectangle and enlarged in top right panel. Corresponding model in bottom right panel well matches with the DOF monomer in the image shown above. **c,** Size distribution of R-octa DOFs obtained from dynamic light scattering (DLS) measurement. Source data are provided as a Source Data file.

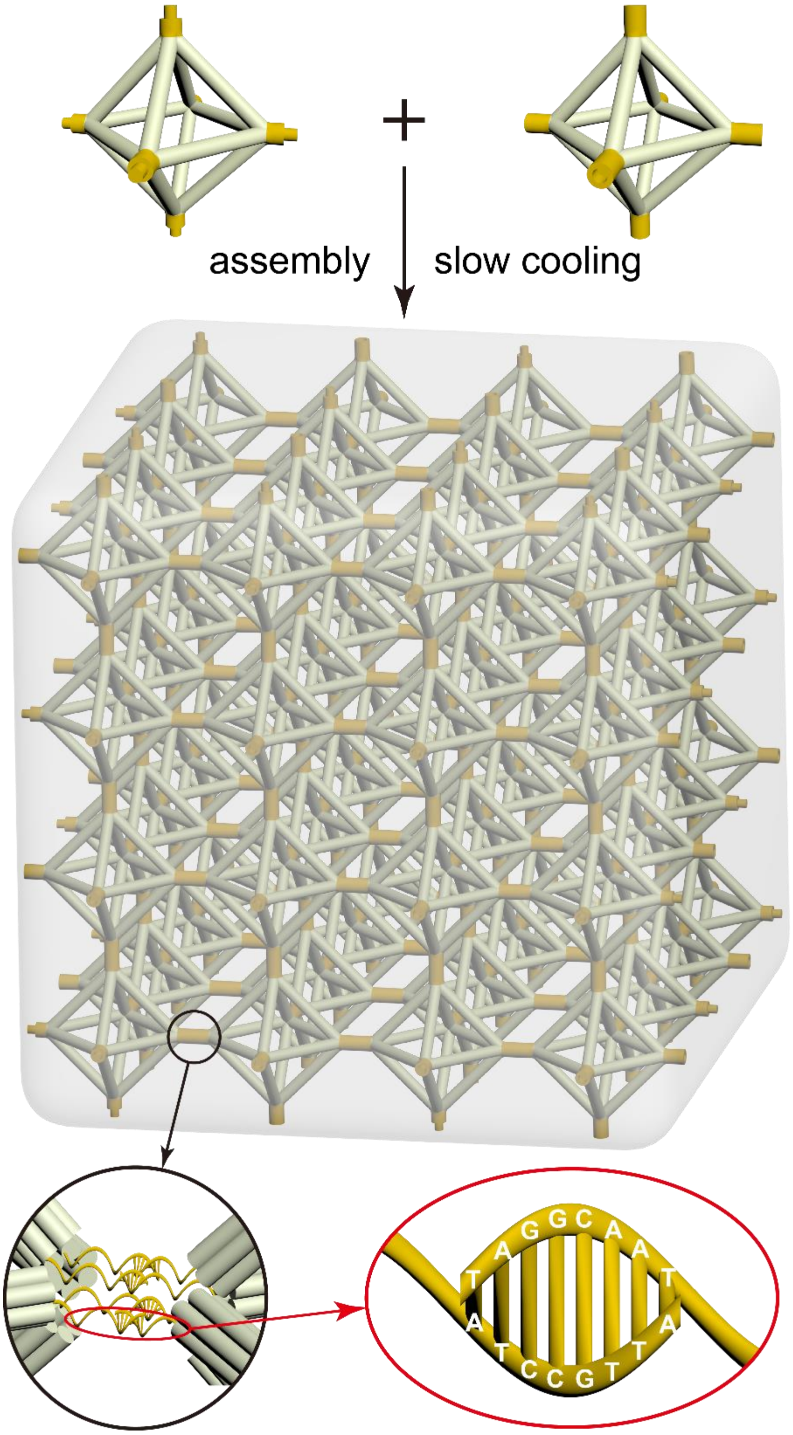

Supplementary Figure 2. Assembly process of cubic microcrystals. Fabrication principal of cubic microcrystals is shown with schematic illustration and a representative partially complementary domain among R-octa DOF monomers is emphatically shown. Corresponding sequences of complementary DNA duplexes are provided as well.

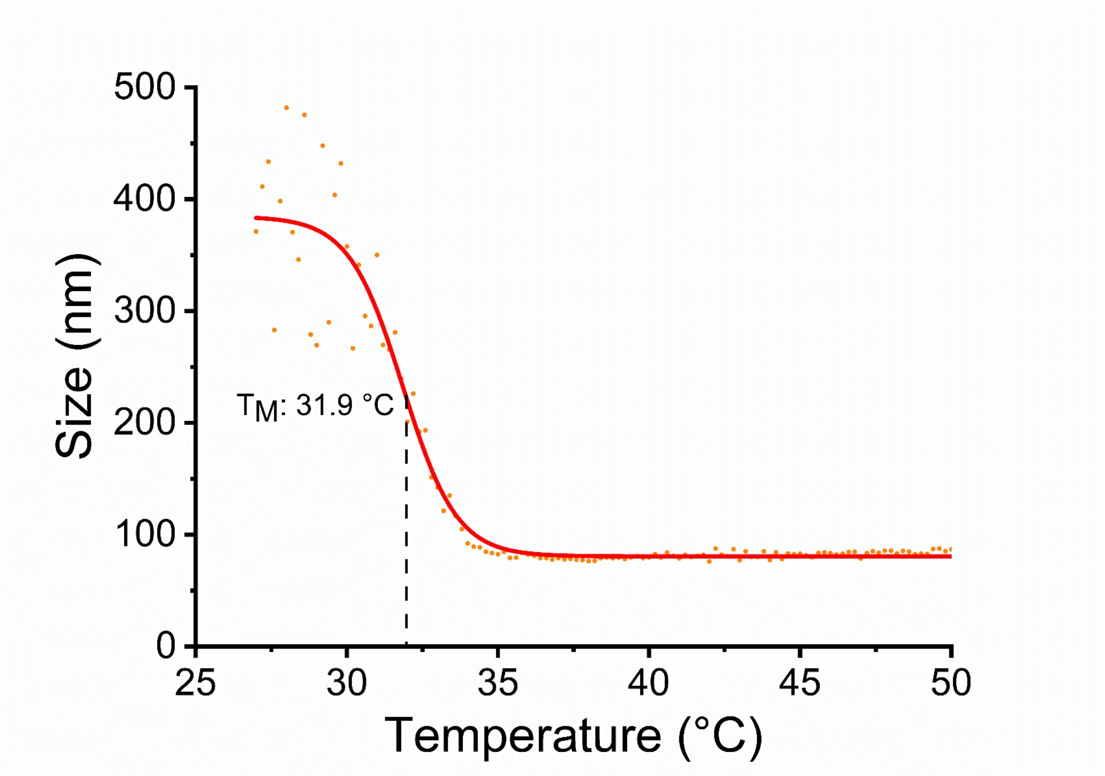

Supplementary Figure 3. Melting temperature (T_M) measurement when fabricating cubic microcrystals. The change of particle sizes is monitored during thermal annealing procedure with the cooling rate of -0.2 °C/2 min. Measurement is conducted with DLS instrument and the result is processed by Boltzmann fitting method, which reveals the approximate melting temperature ~31.9 °C. Source data are provided as a Source Data file.

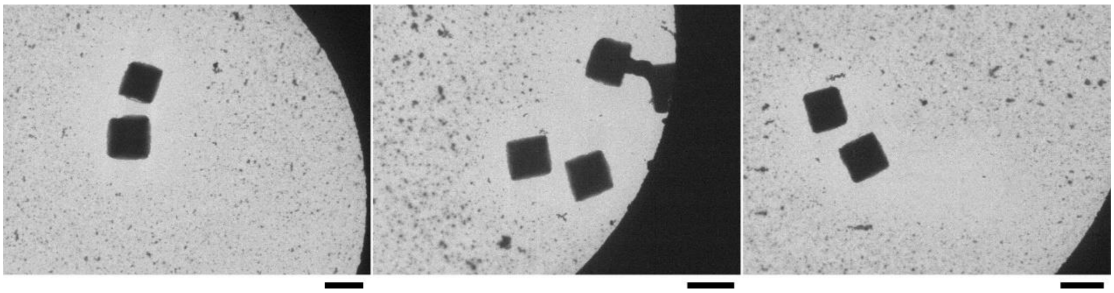

Supplementary Figure 4. Low-magnification TEM images of bare cubic microcrystals. TEM images of monodispersed microcrystals observed in a very low magnification. Negative staining is carried out and grains visibly present the profile of cubic polyhedra. Scale bars: 5 μm.

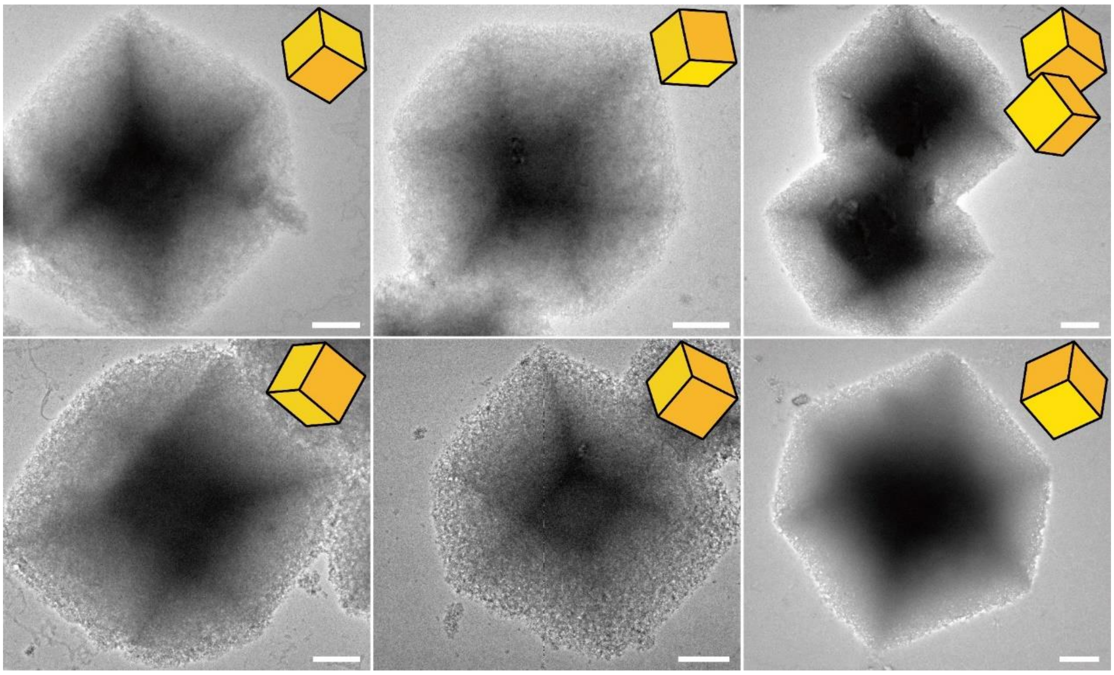

Supplementary Figure 5. TEM images of bare cubic microcrystals. Close-up TEM images of multiple unencapsulated cubic microcrystals. Negative staining is carried out and orientations of crystals are well matched with respective models (top right in orange color). Scale bars: 1 μm.

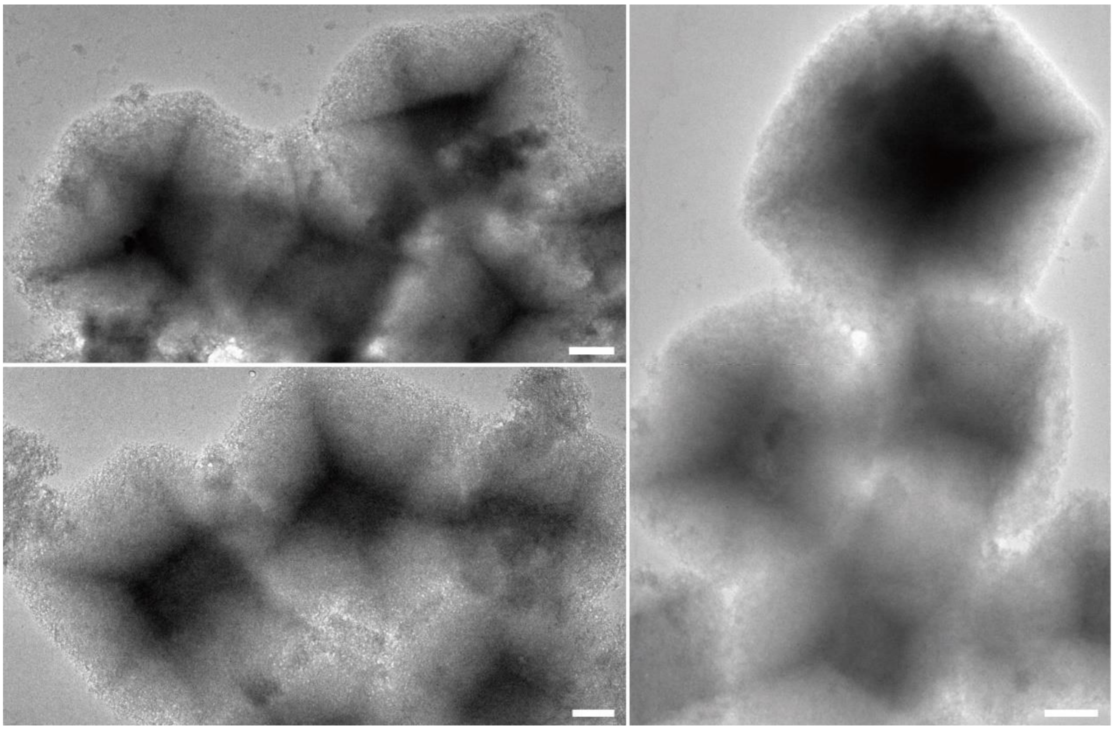

Supplementary Figure 6. TEM images of stacked cubic microcrystals. Extensively generated cubic crystals are observed as well during the visualization process of unencapsulated microcrystals. Scale bars: 1 μm.

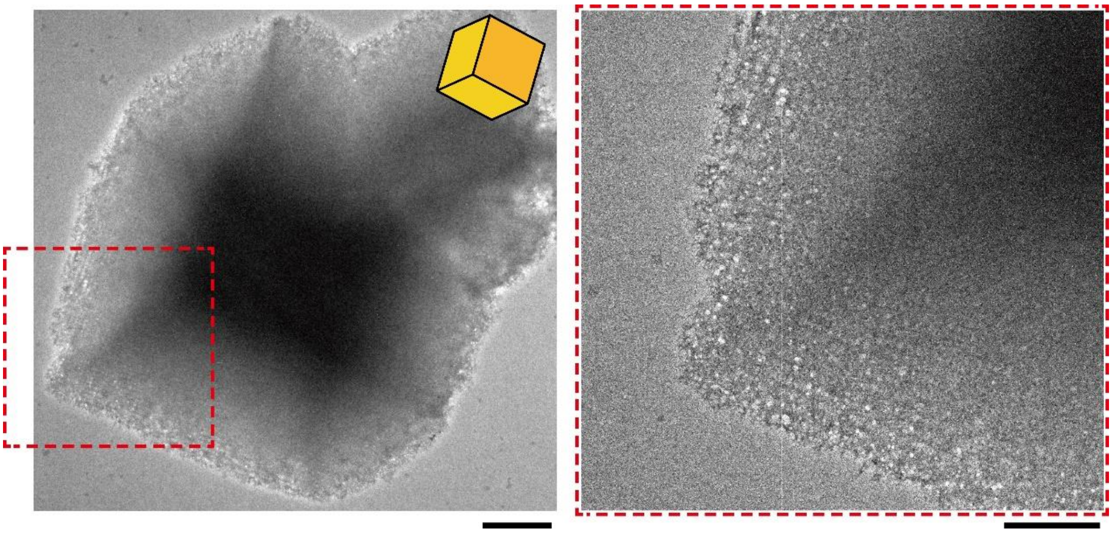

Supplementary Figure 7. Structural determination of unencapsulated cubic microcrystal. A beam permeable region is selected from the edge of entire microcrystal (left panel) and enlarged (right panel) for further observation. Geometric configuration and arrangement of unencapsulated R-octa DOFs cannot be identified even if the observation process is conducted under such high magnification. Scale bars, left panel: 1 μm, right panel: 0.5 μm.

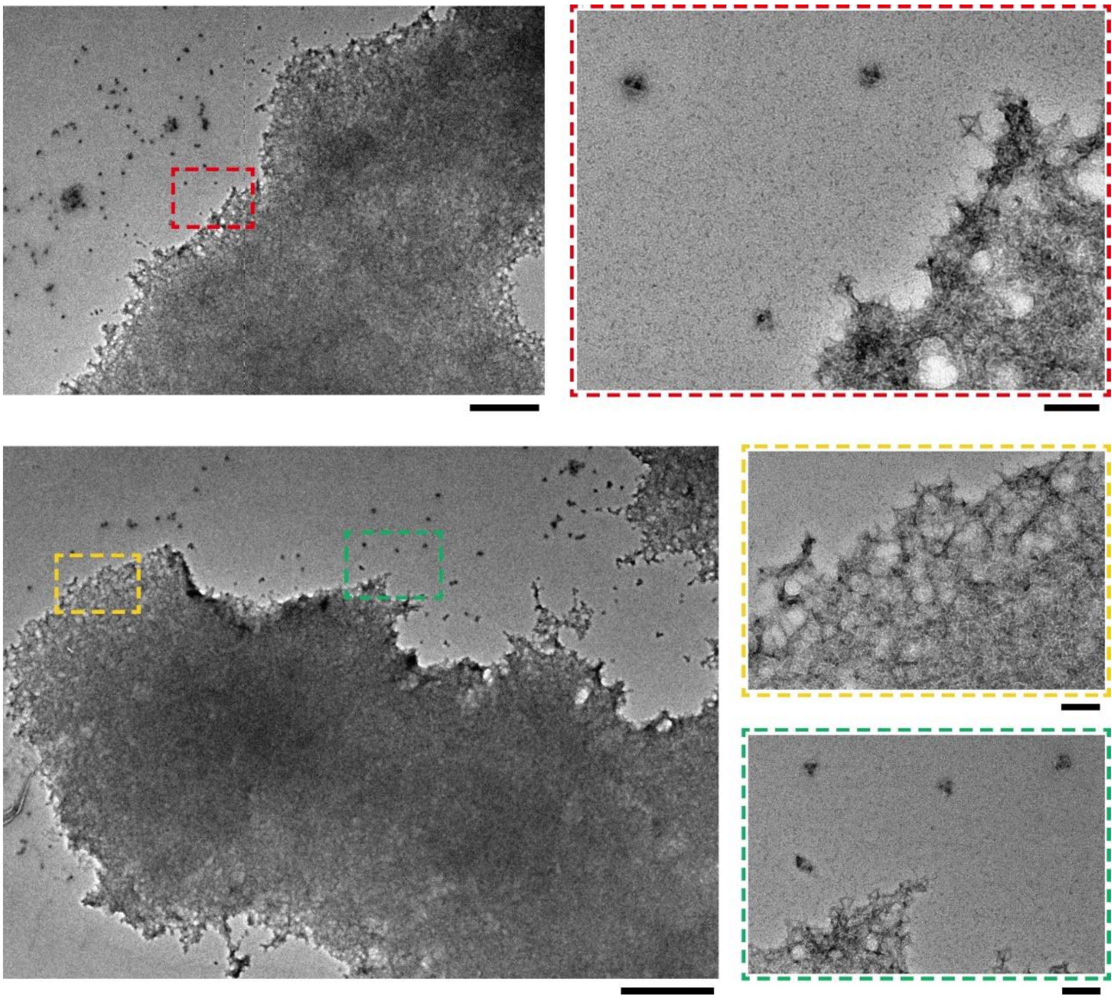

Supplementary Figure 8. Verification of stability under the low cation concentration for cubic microcrystals. TEM images reveal the collapse and deformation of un-encapsulated microcrystals after decreasing the concentration of $MgCl_2$ in the solution to 5 mM. Partial regions are arbitrarily selected from the edge of distorted crystals and enclosed by red, yellow and green rectangles, respectively. Corresponding regions are zoomed in and dark dots which densely surround microcrystals are confirmed to be DOFs monomers dissociated from the collapsed crystals. Scale bars, zoom out: 1 μm, zoom in: 100 nm.

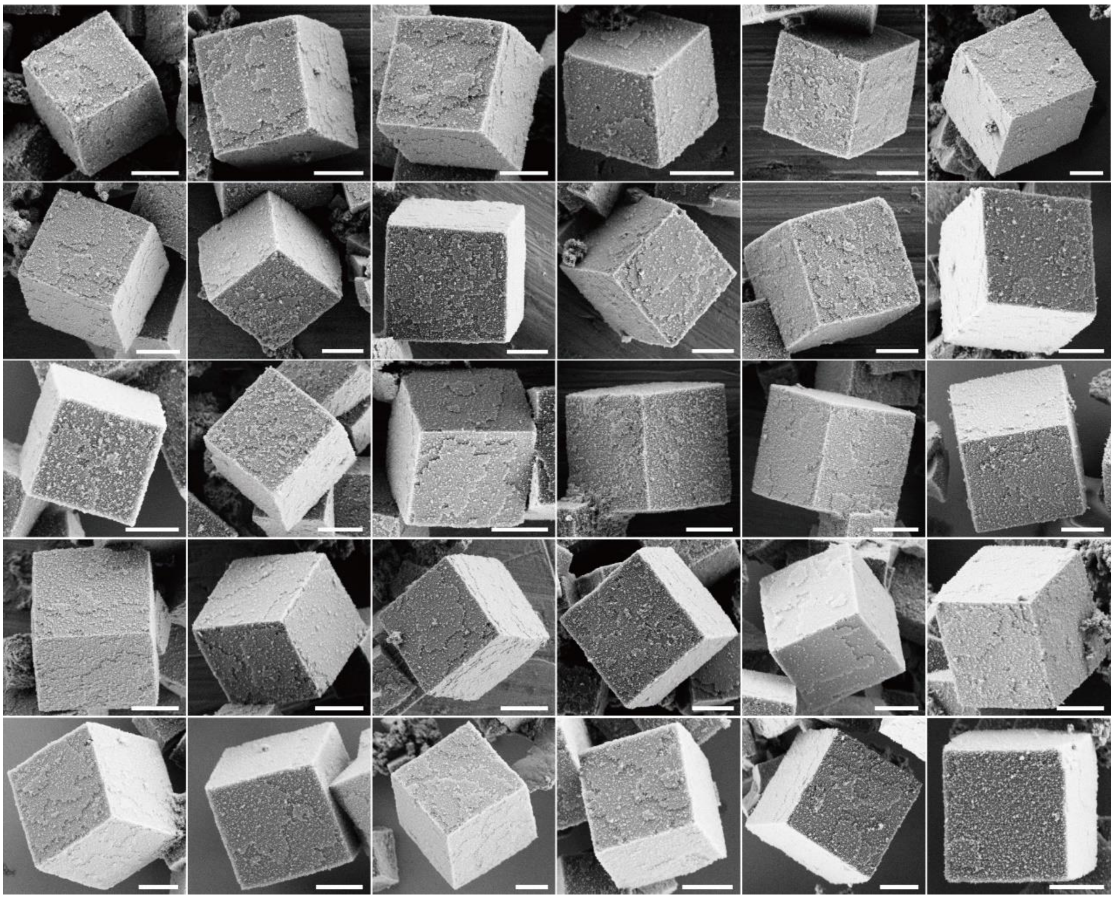

Supplementary Figure 9. SEM images of encapsulated cubic microcrystals. Grains with apparent cubic shapes and varying orientations are distinctly observed through SEM imaging. Scale bars: 2 μm.

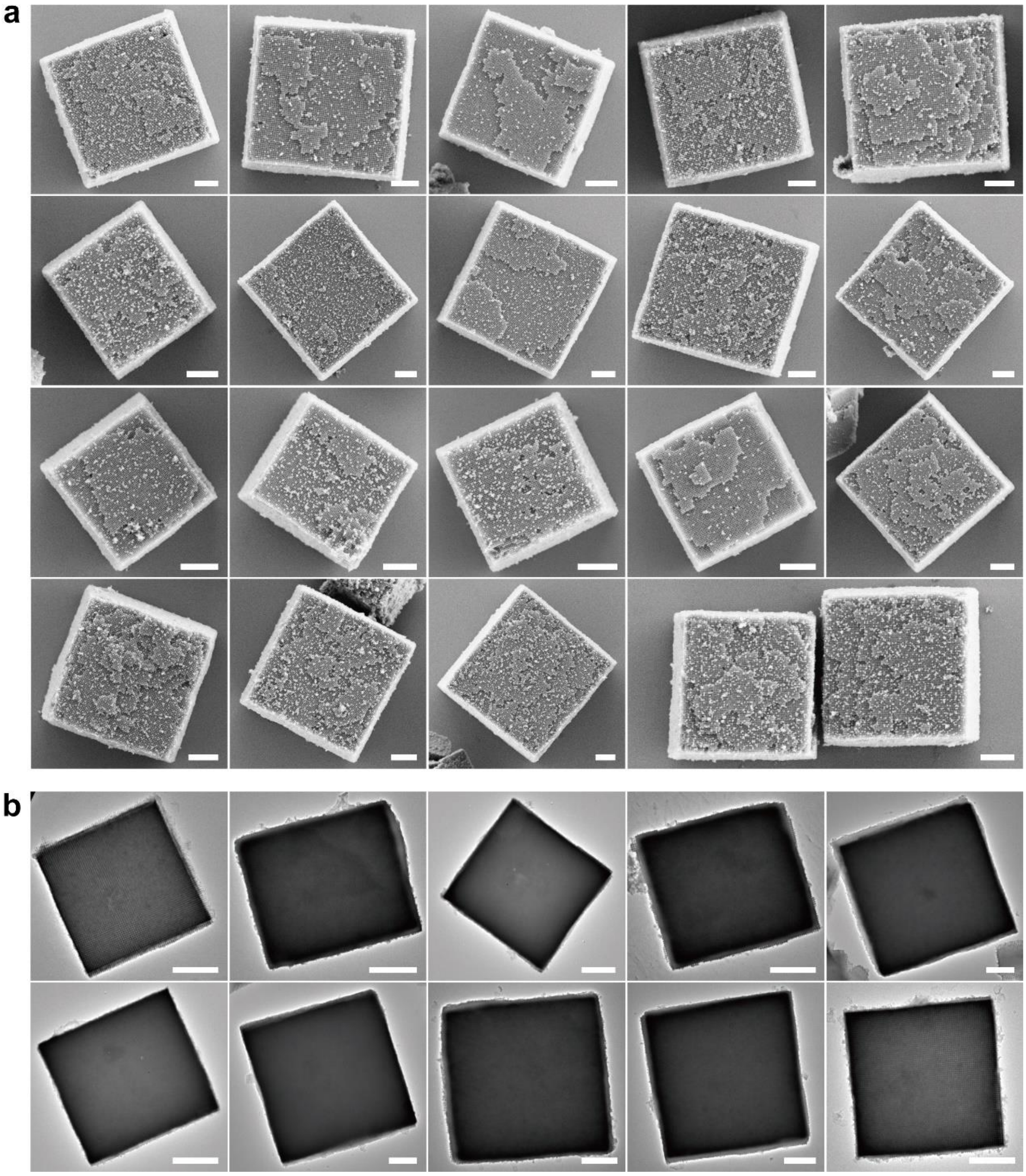

Supplementary Figure 10. EM images of consistently oriented free-standing microcrystals. Manifold cubic crystals with face-up state are observed SEM (**a**) and TEM (**b**) imaging. The uniform orientations can be rationalized by the ‘hard landing’ of reinforced silica-DNA microcrystals when depositing on the substrates (silicon wafers/carbon coated copper grids). Scale bars: 1 μm.

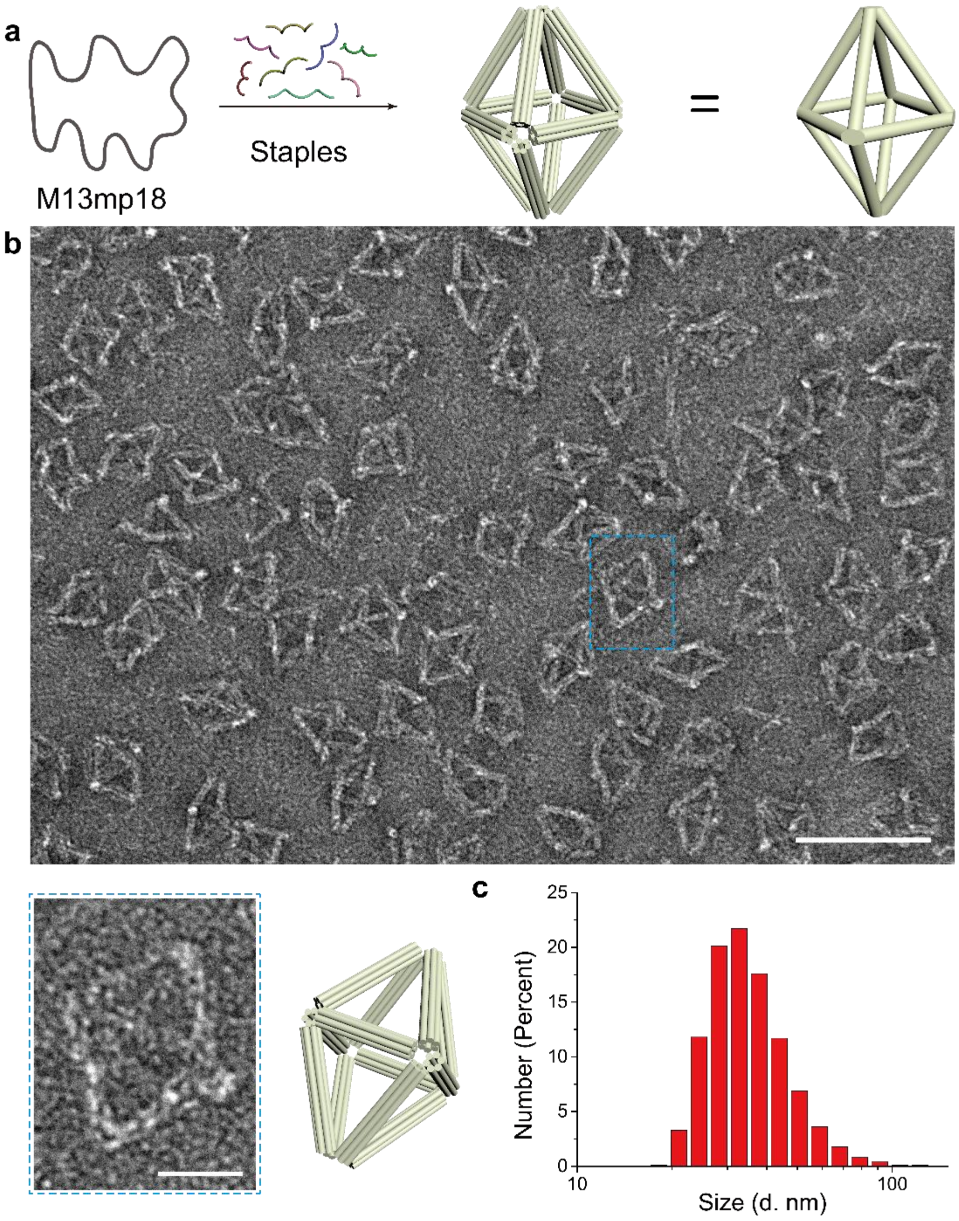

Supplementary Figure 11. Schematic illustration of the assembly process for E-octa DOFs and corresponding characterizations. a, Assembly process of E-octa DOFs, each bundle of a DOF is composed of a 6HB as well. **b,** TEM image of negative stained E-octa DOFs. A representative individual E-octa DOF is enclosed by blue dashed rectangle and enlarged in top right panel. Corresponding model in bottom right panel well matches with the DOF monomer in the image above. **c,** Size distribution of E-octa DOFs obtained from DLS measurement. Source data are provided as a Source Data file.

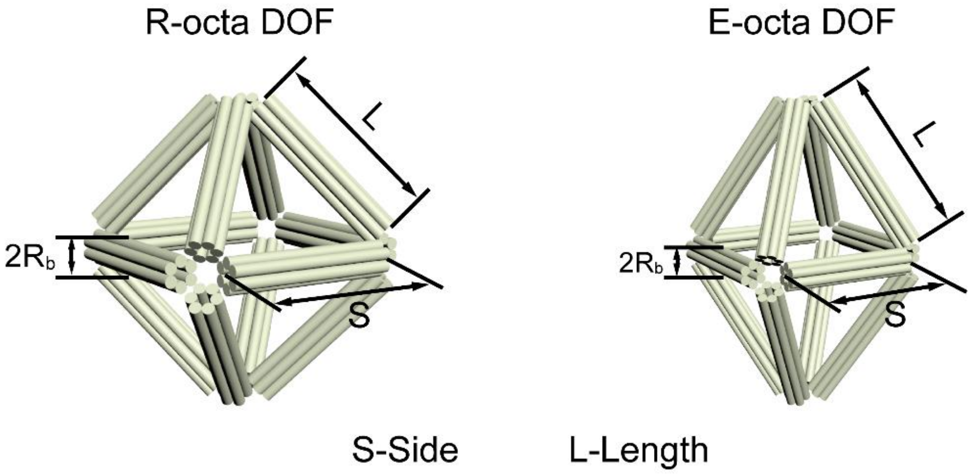

	holistic view	front view	side view	overhead view
Regular octahedron	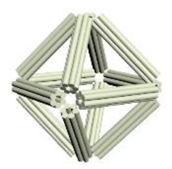	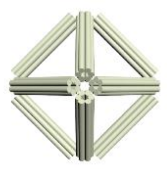	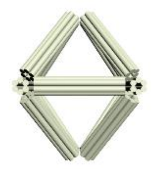	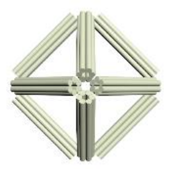
Geometric parameters	S = L = 28.56 nm (84 bp)<br>R_b = 3 nm			
	holistic view	front view	side view	overhead view
Elongated octahedron	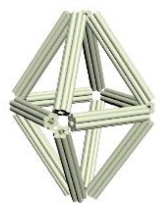	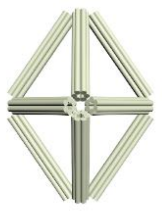	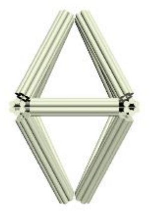	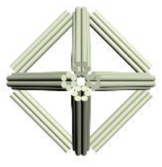
Geometric parameters	S = 28.56 nm (84 bp) L= 35.7 nm (105 bp)<br>R_b = 3 nm			

Supplementary Figure 12. Comparison of geometric parameters of R-octa and E-octa DOFs.

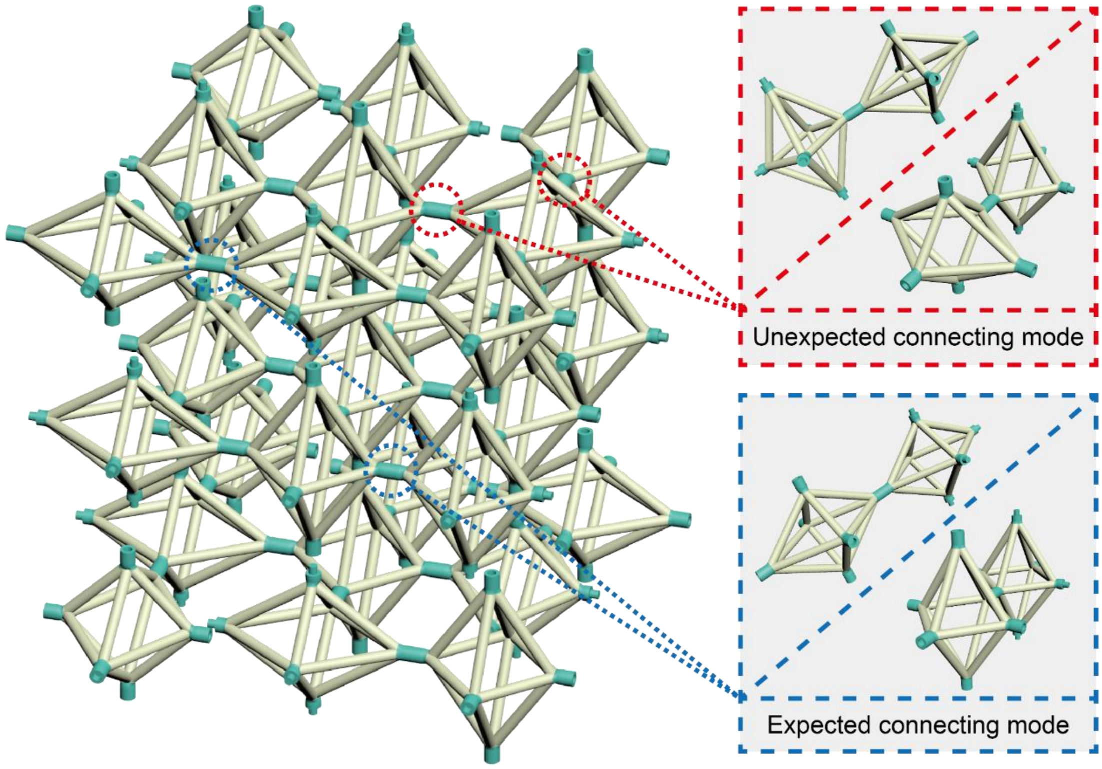

Supplementary Figure 13. Different binding modes of E-octa DOF monomers. According to our design, interconnection of E-octa DOFs can only be triggered within sticky ends extending from four vertices of middle-planes of E-octa DOFs and misconnection within six vertices is prohibited. The schematic illustrates that how unexpected binding mode will be induced if sequences of six sticky ends are designed identically (represented by green concavo-convex cylinders). Expected and unexpected binding mode are labelled with blue and red and are emphatically shown, respectively.

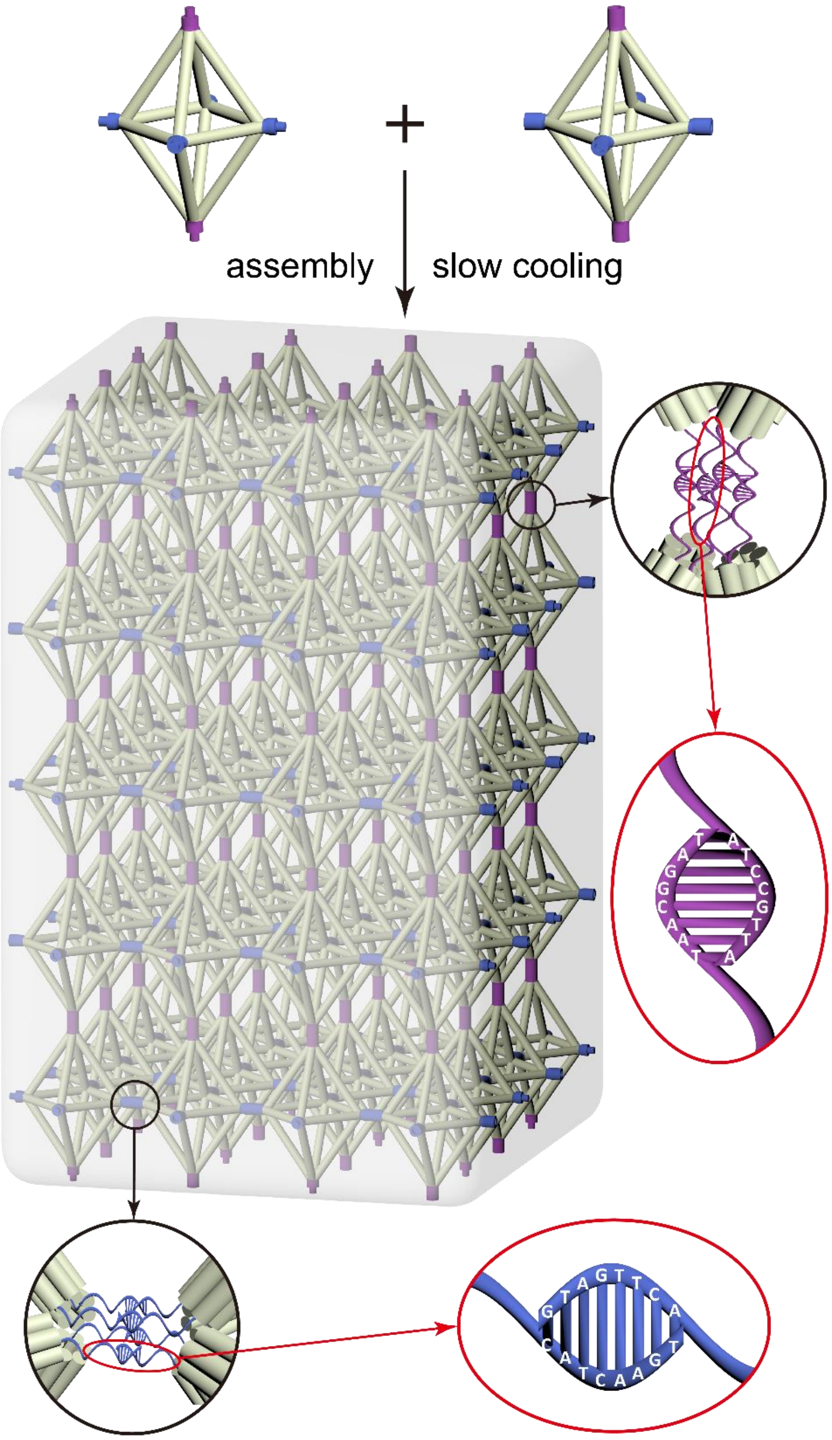

Supplementary Figure 14. Assembly process of cuboid microcrystals. Fabrication principal of cuboid microcrystals is shown with schematic illustration and two kinds of partially complementary domains among E-octa DOF monomers are emphatically shown. Corresponding sequences of complementary DNA duplexes are provided as well.

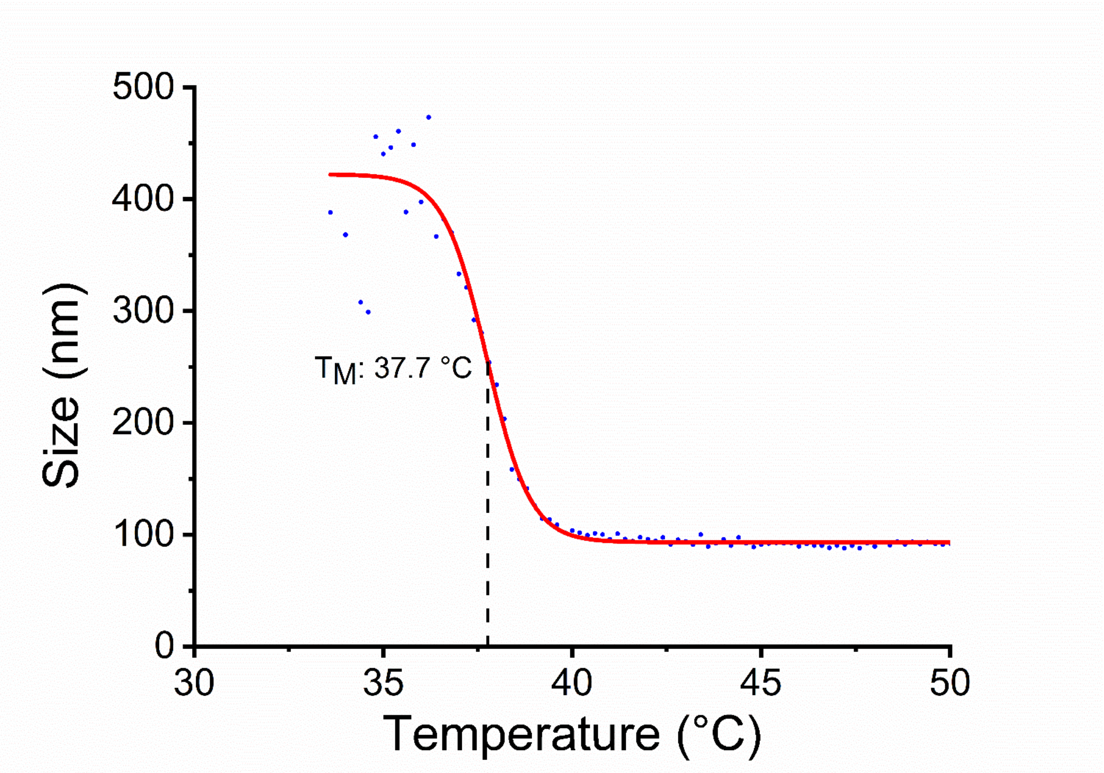

Supplementary Figure 15. Melting temperature (T_M) measurement when fabricating cuboid microcrystals. Like R-octa DOFs, the change of particle sizes is monitored during thermal annealing procedure with the cooling rate of -0.2 °C/2 min as well. Measurement is conducted with DLS instrument and the result is processed by Boltzmann fitting method, which reveals the approximate melting temperature ~37.7 °C. Source data are provided as a Source Data file.

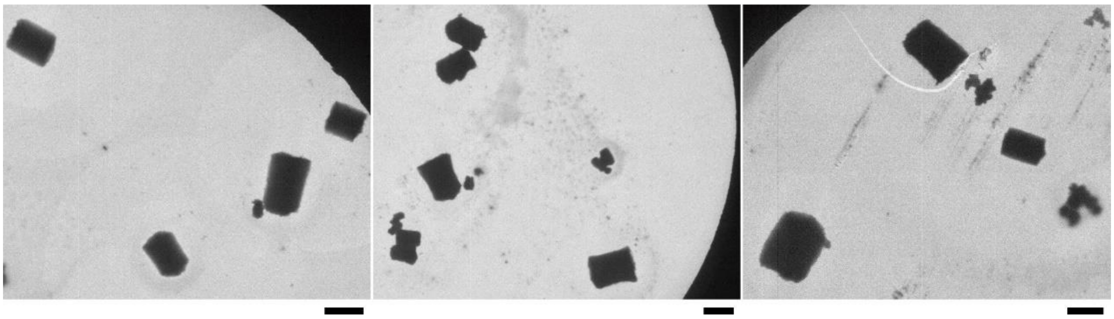

Supplementary Figure 16. Low-magnification TEM images of bare cuboid microcrystals. TEM images of monodispersed microcrystals observed in a very low magnification. Negative staining is carried out and grains visibly present the profile of cuboid polyhedra. Scale bars: 5 μm.

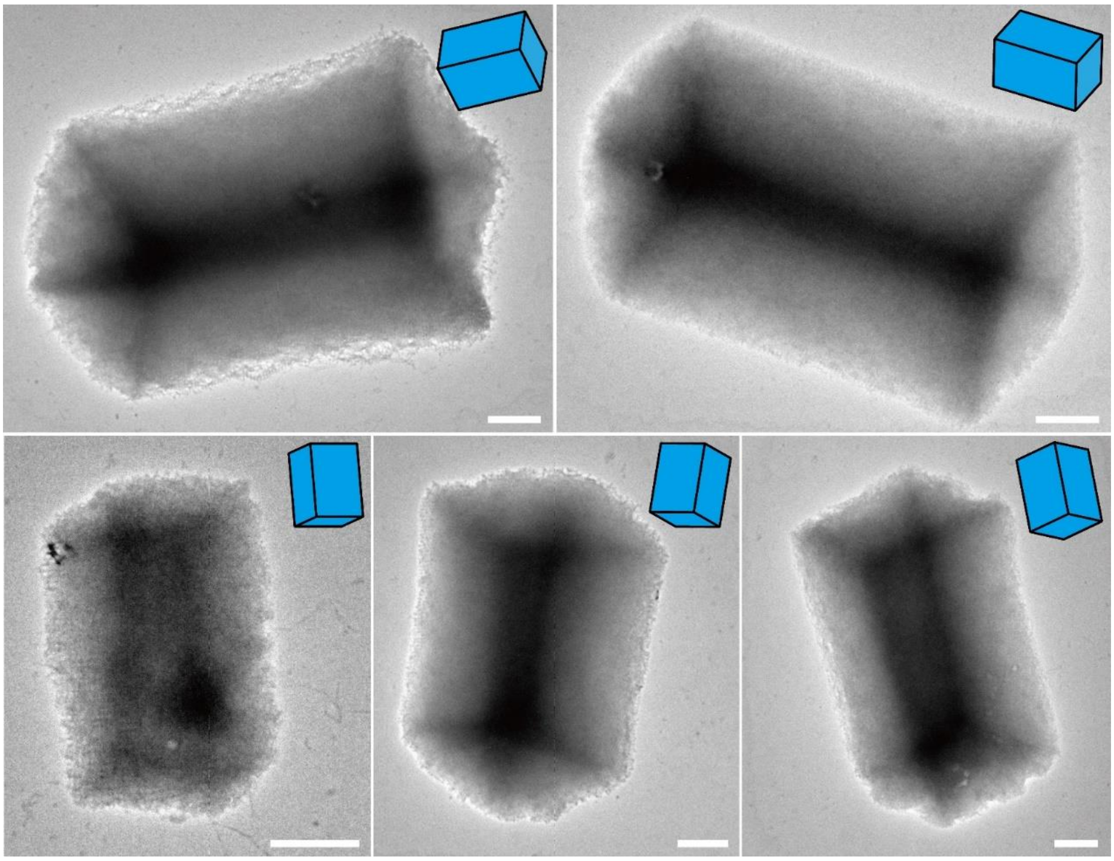

Supplementary Figure 17. TEM images of bare cuboid microcrystals. Close-up views of multiple unencapsulated cuboid microcrystals. Negative staining is carried out and orientations of crystals are well matched with the respective models (top right). Scale bars: 1 μm.

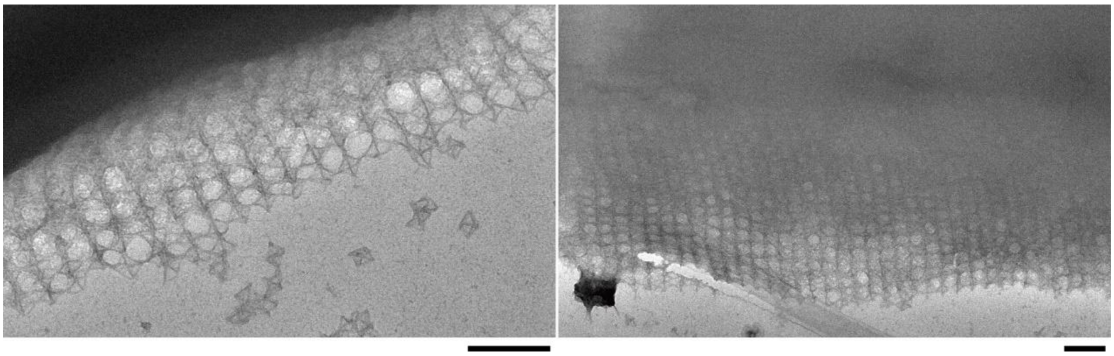

Supplementary Figure 18. TEM images of the edge part of unencapsulated cuboid microcrystals. The arrangement of DOF monomers can be directly observed. Scale bars: 200 nm.

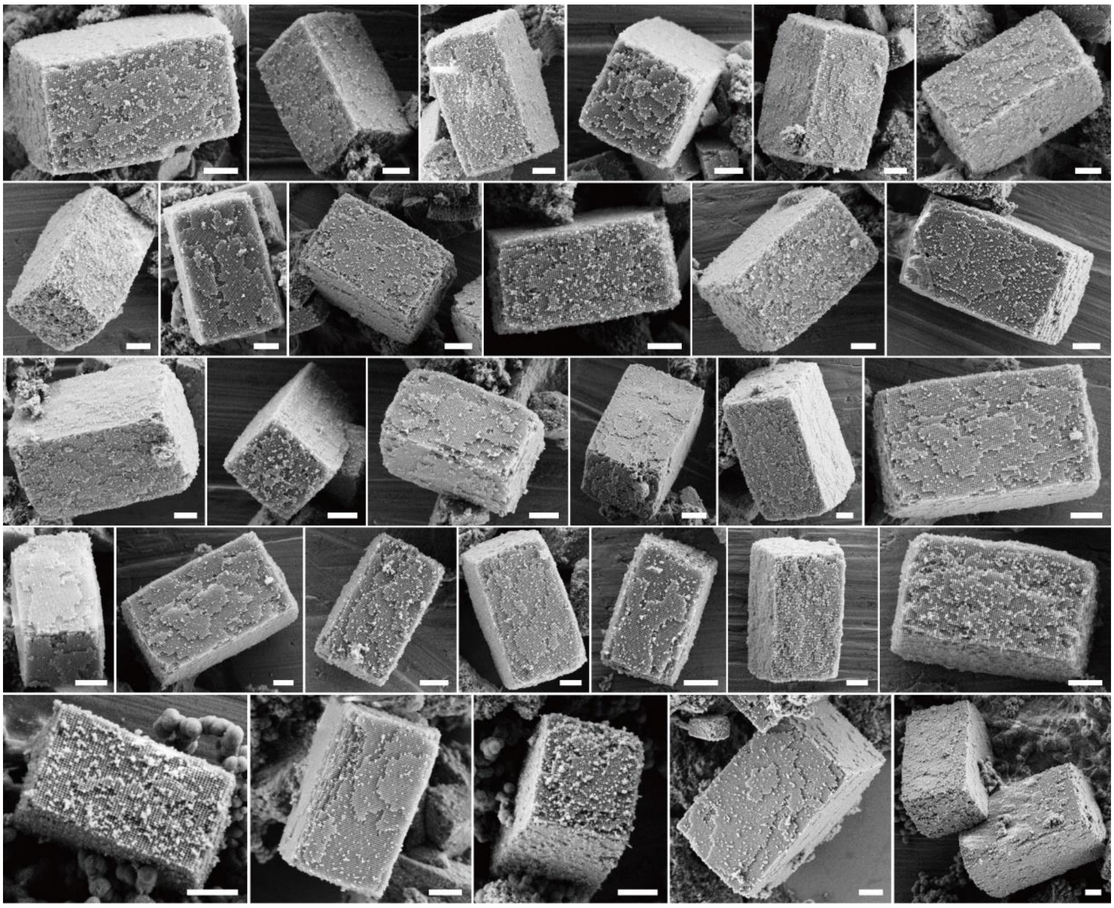

Supplementary Figure 19. SEM images of encapsulated cuboid microcrystals. Holistic view of silicified cuboid grains with varying orientations are obtained using SEM. Scale bars: 1 μm.

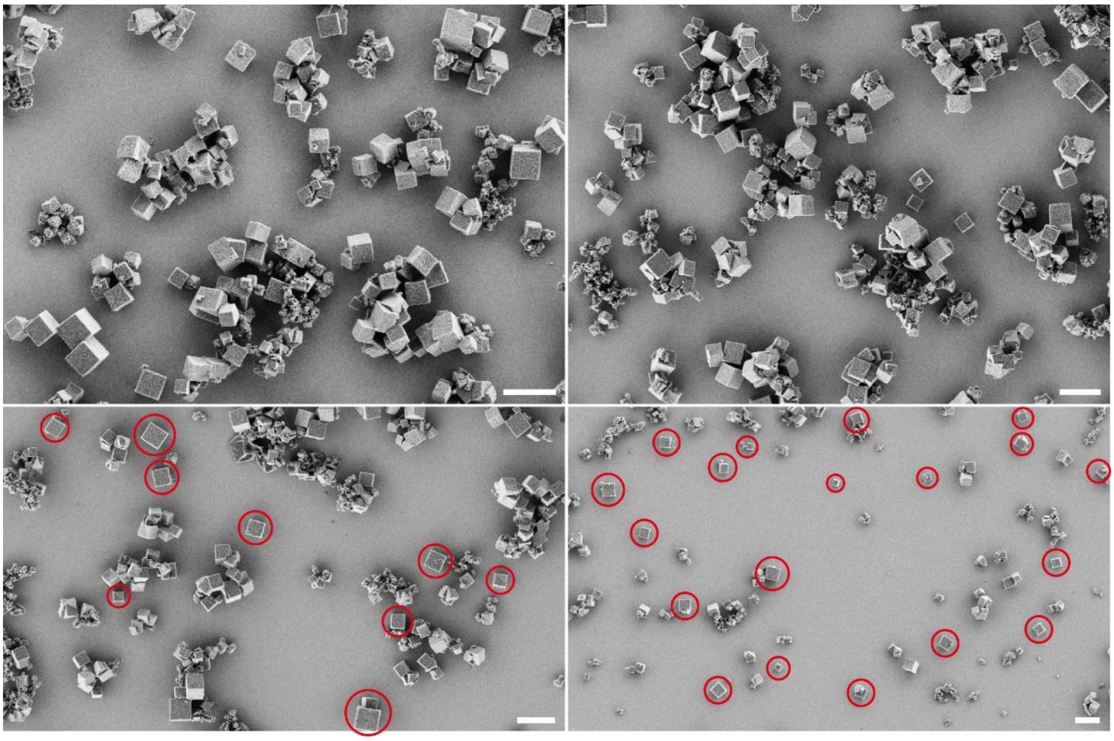

Supplementary Figure 20. Verification of the yield of encapsulated cubic microcrystals. Low magnification SEM images display a large amount of solid-state cubic grains on substrates, which reveal the outstanding productivity of crystallization strategy and the shape fidelity after silica-embedding process. Note that several grains which keeping consistent orientations are labelled with red circles in images, some of which are amplified in Supplementary Fig. 10 for illustrating the remarkable phenomenon. Scale bars: 10 μm.

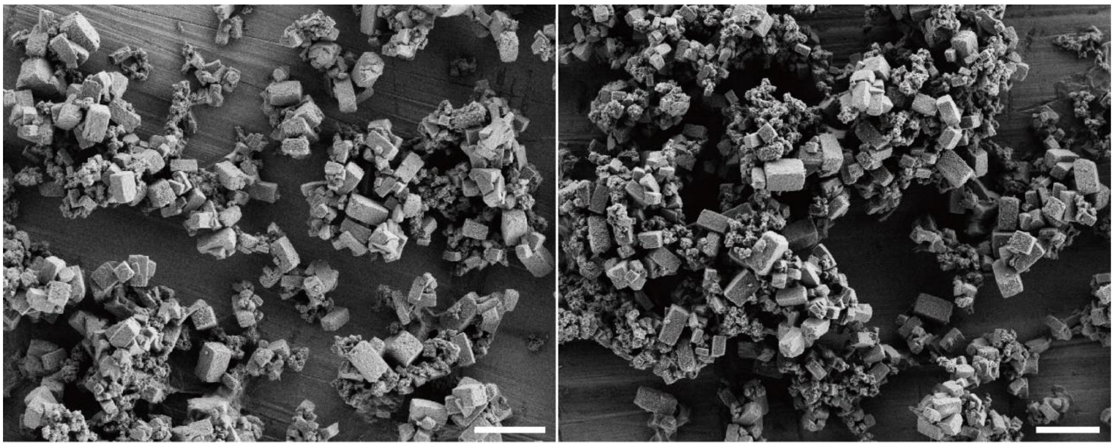

Supplementary Figure 21. Verification of yield of encapsulated cuboid microcrystals. Plentiful solidified cuboid grains are inspected under approximate 80 μm × 70 μm domains. It is difficult to completely remove concomitant silica chunks when collecting the desired encapsulated structures despite the introduction of purification procedure. Scale bars: 10 μm.

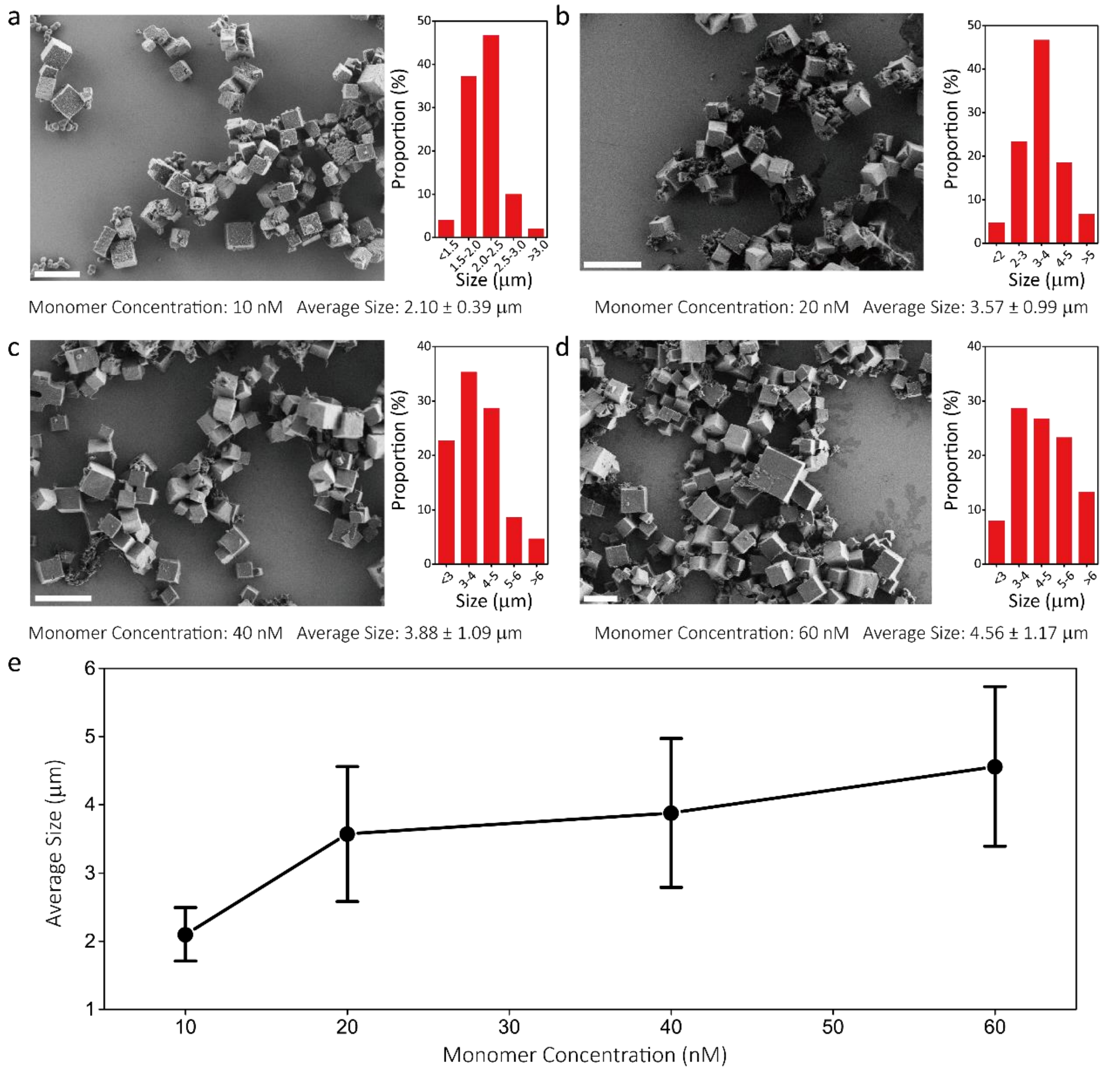

Supplementary Figure 22. SEM images and size distributions of encapsulated cubic microcrystals made of different concentrations. Scale bars, a: 5 μm, b, c, d: 10 μm. e, average size of crystal under different monomer concentrations, error bars represent the SD. Source data are provided as a Source Data file.

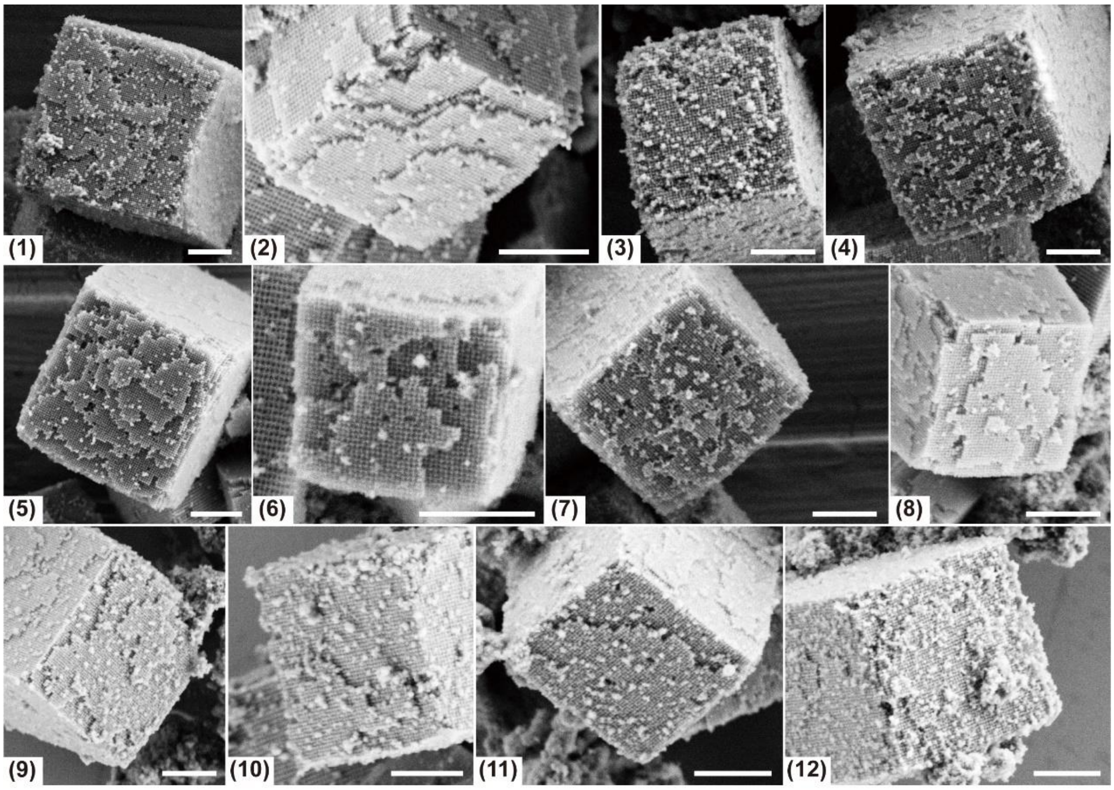

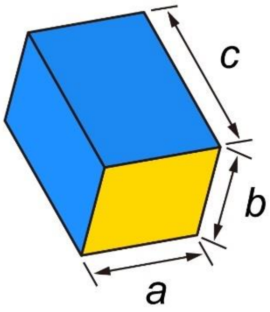

Number	$a/\mu m$	$b/\mu m$	a/b	Number	$a/\mu m$	$b/\mu m$	a/b
(1)	3.706	3.865	0.959	(7)	2.731	2.452	1.114
(2)	2.287	2.253	1.015	(8)	1.922	1.963	0.979
(3)	2.511	2.551	0.984	(9)	2.944	2.430	1.212
(4)	3.097	3.059	1.012	(10)	2.131	2.456	0.868
(5)	3.231	3.237	0.998	(11)	2.217	2.382	0.931
(6)	1.611	1.775	0.908	(12)	2.569	2.695	0.953

Supplementary Figure 23. SEM images of subfaces of encapsulated cuboid microcrystals. A series of SEM images (top panel) which directly observe the exposed subfaces of cuboid grains from favorable orientations are used to calculate the ratios of edge lengths. Corresponding edge lengths (*a* and *b*) of subface are illustrated in schematic (bottom left panel) and measured with *image J* software. According to the table shown beside (bottom right panel), *a* and *b* are approximately equivalent, which is the foundation of the statistical analysis of *a* and *c* in main text. Scale bars: 1 μm.

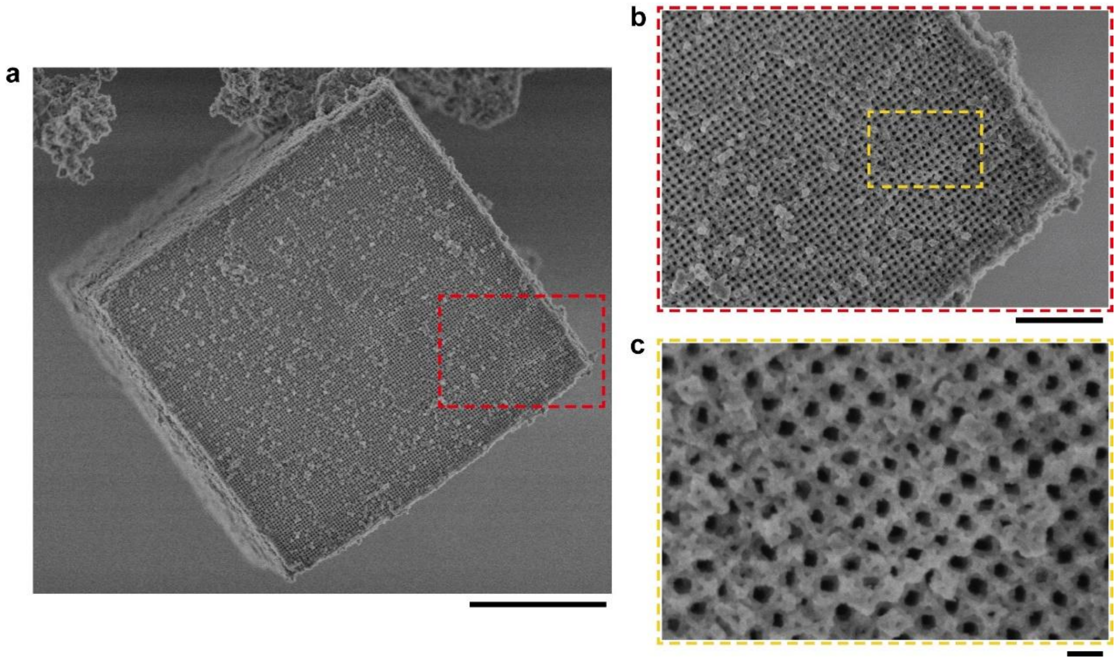

Supplementary Figure 24. Surface structural inspection of encapsulated cubic microcrystal. a, SEM image of a silicified cubic microcrystal. **b,** Close-up view of the partial region arbitrarily selected from the entire grain (enclosed by red dashed rectangle in **a**). **c,** Further observation is carried out as the view focus on a smaller domain (enclosed by yellow dashed rectangle in **b**). Highly ordered building blocks are readily identified to be encapsulated R-octa DOFs from images and the arrangement of which conform to the expected packing mode. Scale bars, a: 2 μm, b: 0.5 μm, c: 50 nm.

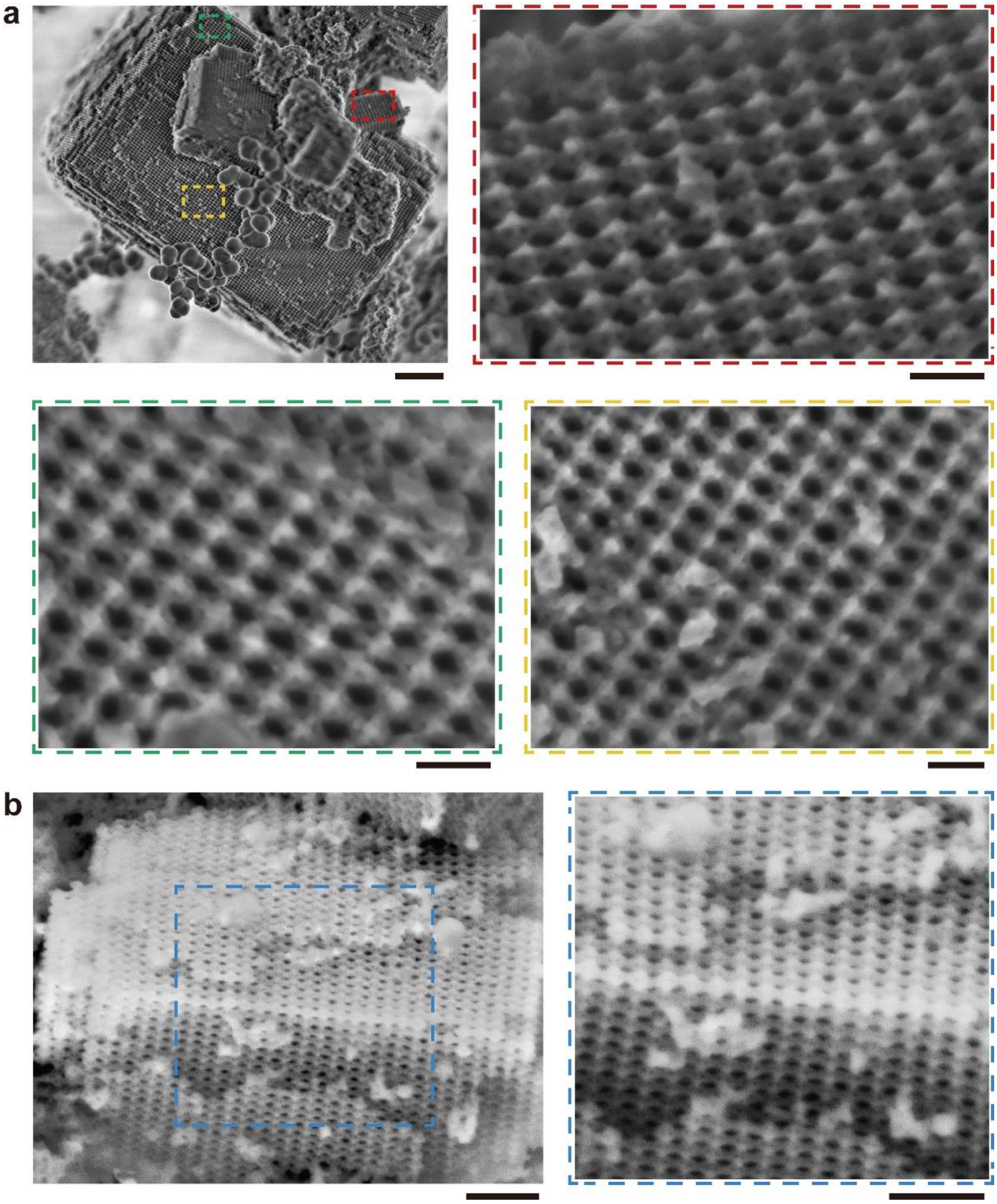

Supplementary Figure 25. Surface structural inspection of encapsulated cuboid microcrystal. SEM image of the entire grain and close-up views of corresponding partial regions (enclosed by red, green, yellow and blue dashed rectangles, respectively) selected from different positions, which indicate the configuration and ordered arrangement of encapsulated E-octa DOF monomers. Images shown in **a** are obtained from JSM-7800F (JEOL) under gentle beam (GB) mode for recognizing the geometric shape of E-octa DOFs. Observation process is operated under the accelerating voltage of 1 kV and such low voltage result in the low image brightness. Scale bars, a, zoom out: 1 μm, zoom in: 100 nm, b: 200 nm.

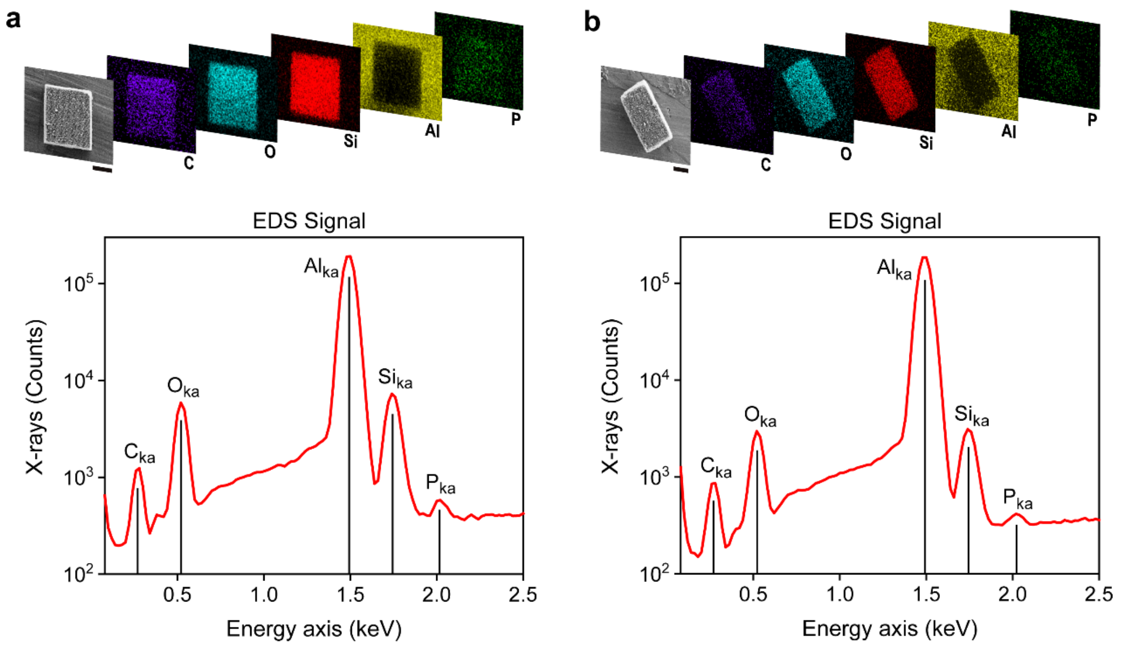

Supplementary Figure 26. EDS analysis of encapsulated microcrystals. EDS mapping of free-standing cubic (**a**, top panel) and cuboid (**b**, top panel) microcrystals depositing on aluminium substrates, which confirm the expected chemical composition of solidified structures. Elemental distribution of C, O, Si, Al, P are marked with purple, cyan, red, yellow and green, respectively. All the elements in mapping images have obvious peaks in corresponding integral spectra (bottom panels of **a** and **b**). Scale bars: 2 μm. Source data are provided as a Source Data file.

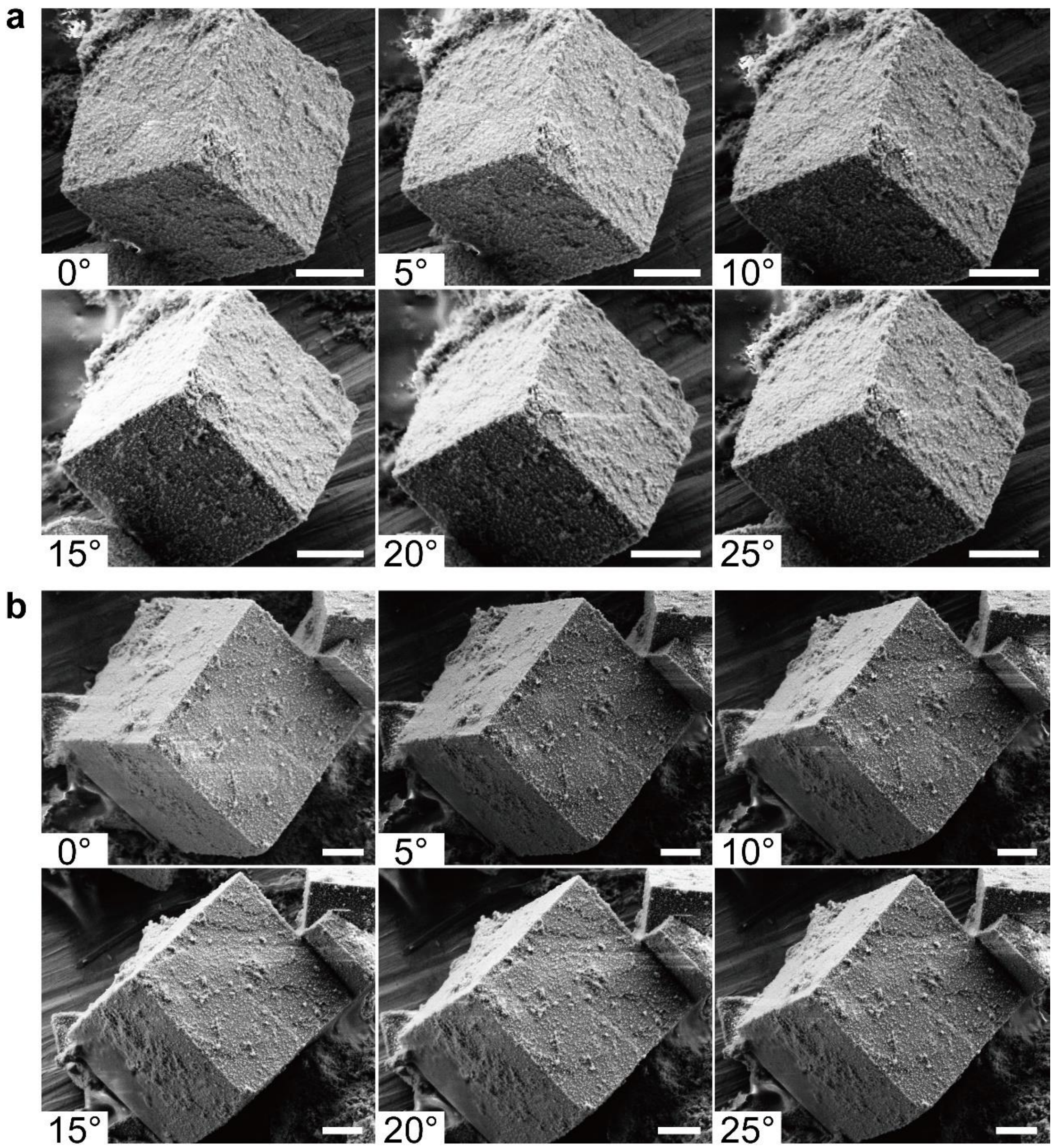

Supplementary Figure 27. SEM tilting experiments. Tilting experiments are carried out on a SEM instrument for intuitively observing the overview of microcrystals. Two cubic grains with different edge lengths (**a**: ~4.6 μm, **b**: ~7.0 μm) are selected for rotation and the tilting procedure is conducted from 0° to 25° with 5° step. Conversion of observed orientations and alterable visibility of facets are monitored during the tilting. Scale bars, 2 μm.

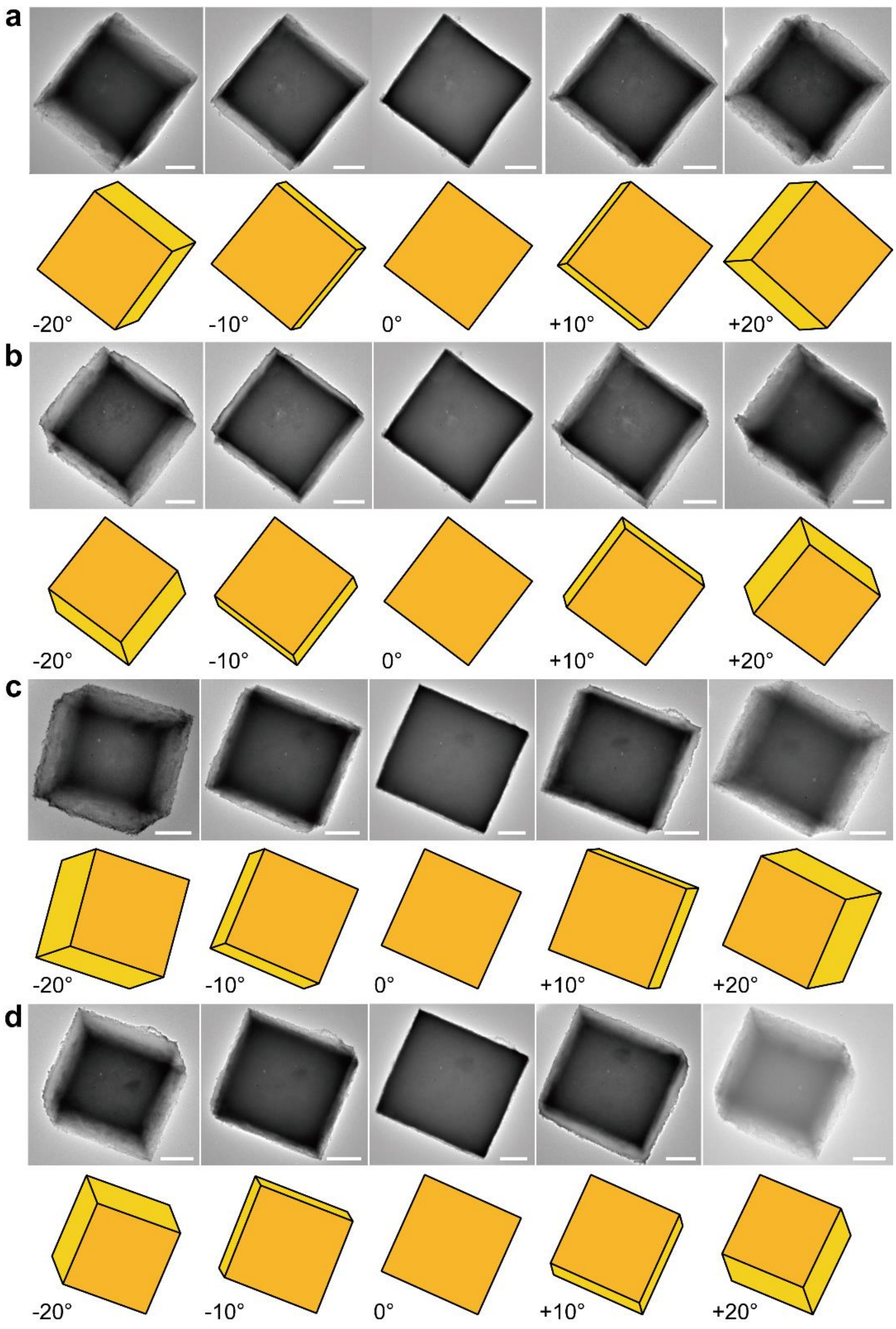

Supplementary Figure 28. TEM tilting experiments. Similar tilting experiments are carried out using TEM and two different cubic grains are driven to rotate along two different axes, respectively. Images of 0° show the original orientations of grains, tilting process along y-axis (**a** and **c**) and x-axis (**b** and **d**) of sample holder are exhibited respectively and the experiments are equally conducted from -20° to 20° with 10° step along two mutually perpendicular axes. Scale bars: 1 μm.

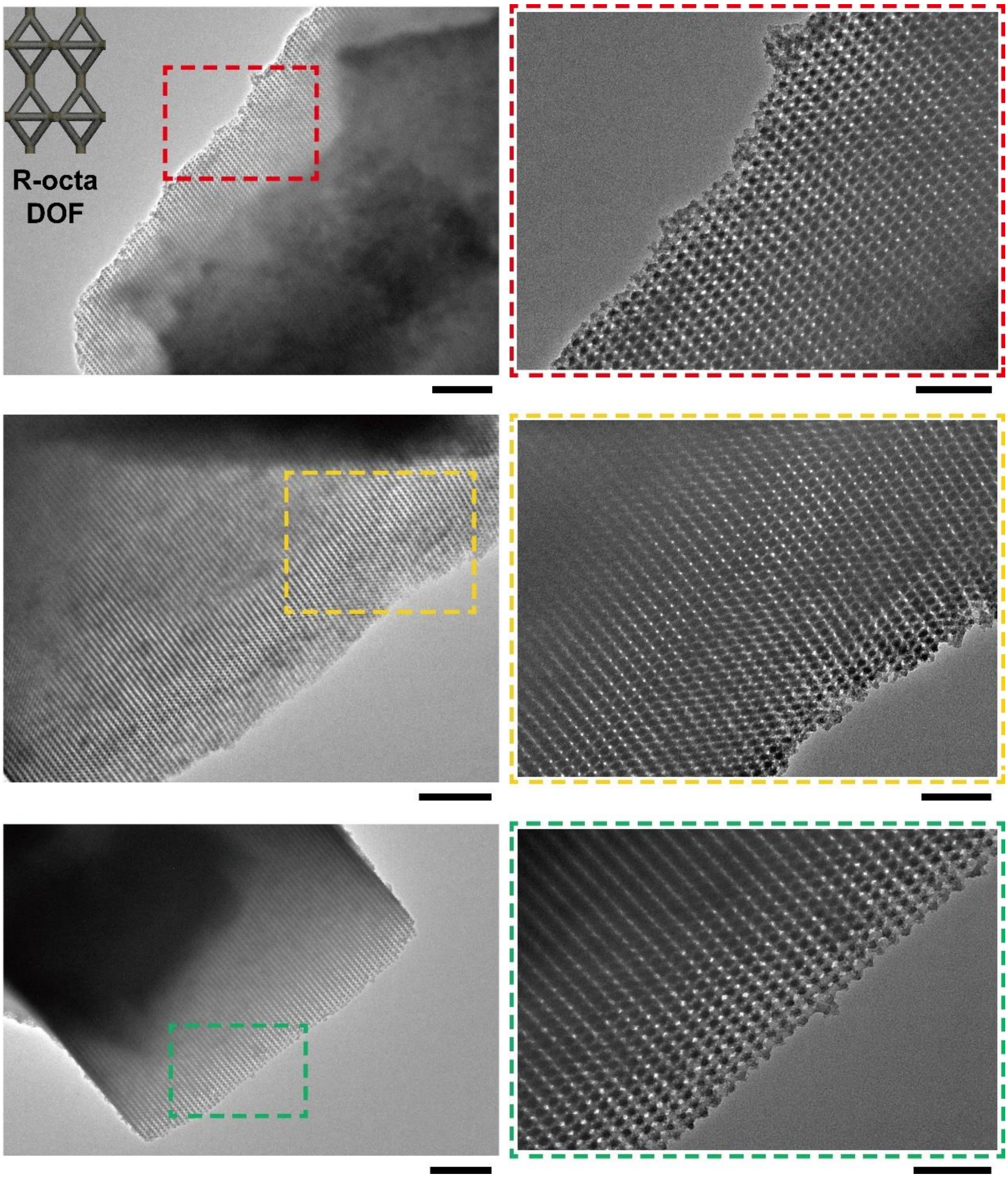

Supplementary Figure 29. TEM structural determination of encapsulated cubic microcrystals. Partial regions are selected from different grains which display appropriate contrast at the edges for precisely observing the arrangement of building blocks. Close-up views of corresponding regions (enclosed by red, yellow and green dashed rectangles, respectively) convincingly validate the long-range ordering of R-octa DOF monomers after encapsulation. Scale bars, zoom out: 1 μm, zoom in: 200 nm.

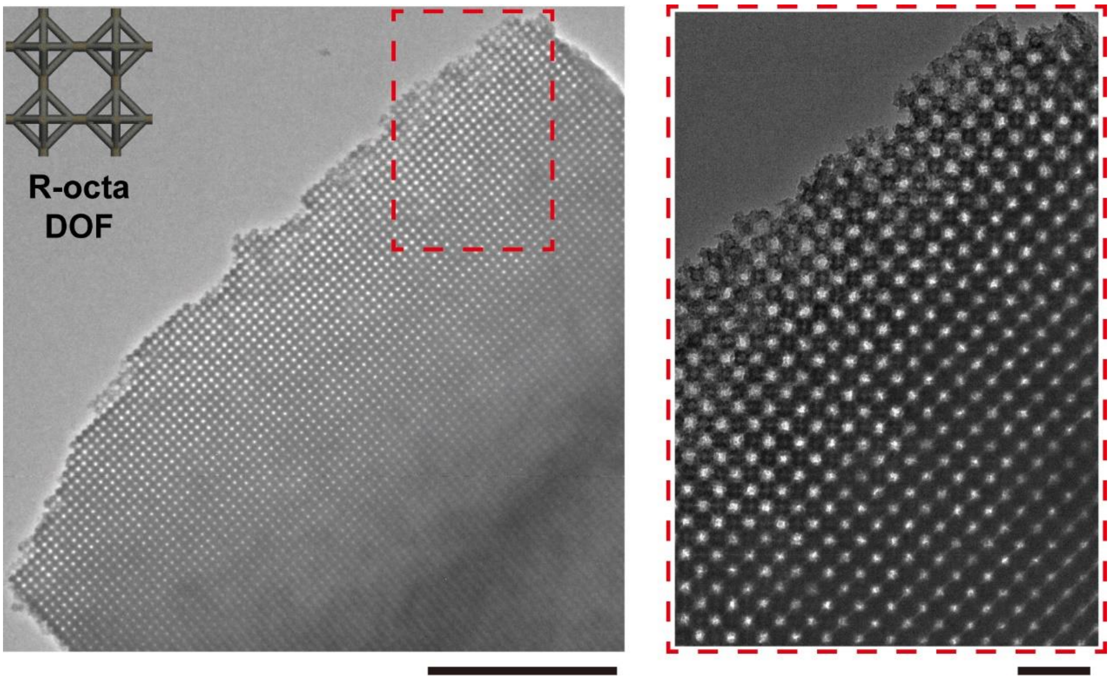

Supplementary Figure 30. TEM observation of surface configuration of an encapsulated cubic grain. A partial fragment is selected from the grain which is favorable for observation along [100] orientation. The close-up view indicates the periodic packing of R-octa DOFs. Scale bars, zoom out: 1 µm, zoom in: 100 nm.

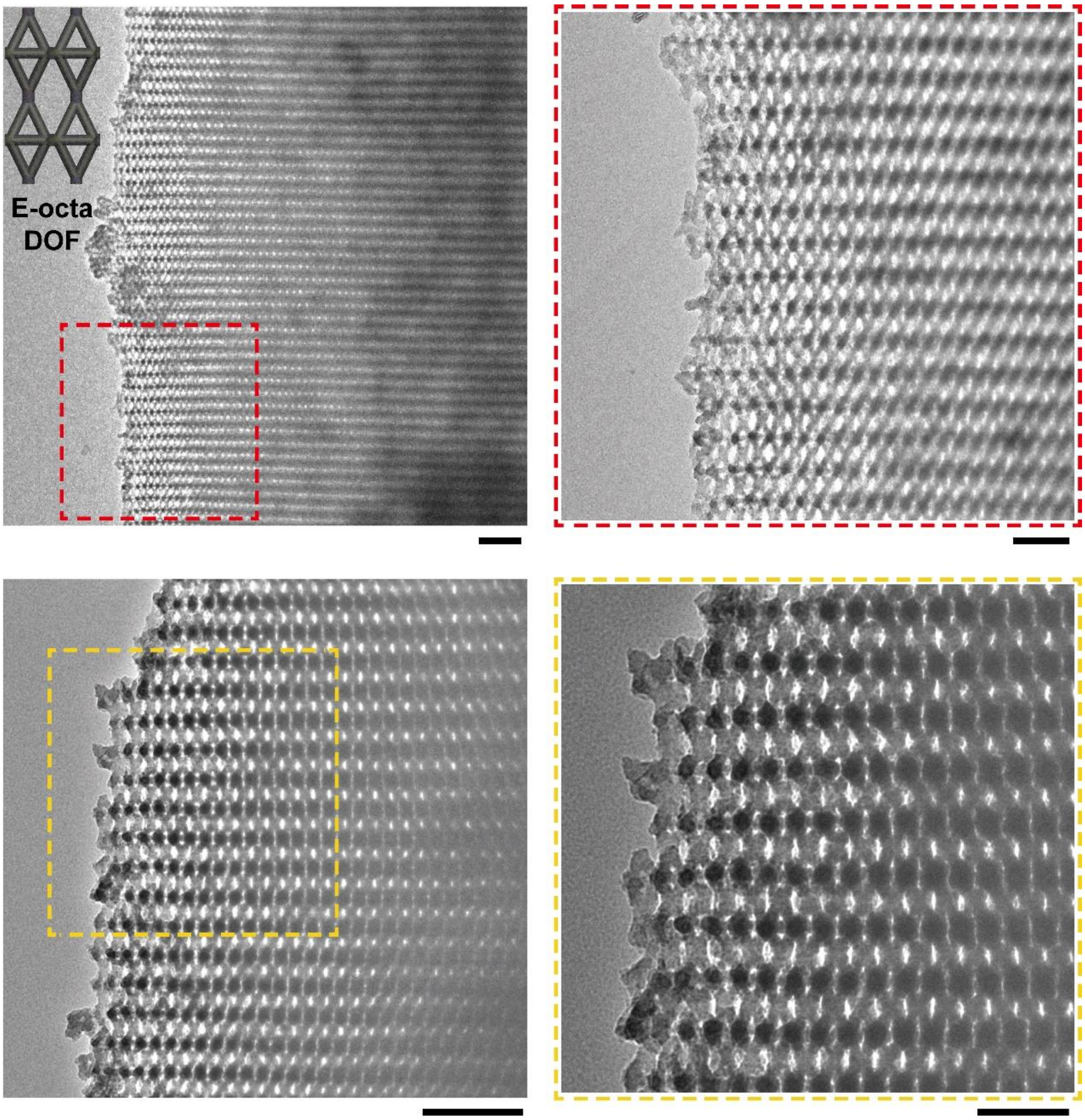

Supplementary Figure 31. TEM structural determination of encapsulated cuboid microcrystals. Partial regions are selected from different grains to validate the highly ordered organization of encapsulated E-octa DOFs which is familiar with R-octa DOFs. Close-up views of corresponding regions (enclosed by red and yellow dashed rectangles, respectively) demonstrate the long-range ordering of E-octa DOF monomers after encapsulation as well. Scale bars, zoom out: 200 nm, zoom in: 100 nm.

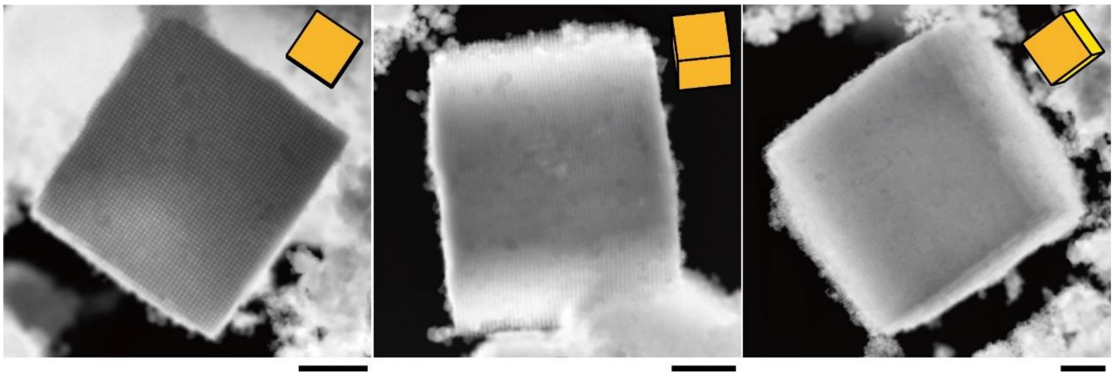

Supplementary Figure 32. STEM images of encapsulated cubic microcrystals. Integral views of microcrystals are obtained using HAADF-STEM. The meshed pattern emerging on the surface of the crystal (far left panel) demonstrates the periodic arrangement of the encapsulated R-octa DOF monomers. Scale bars: 1 μm.

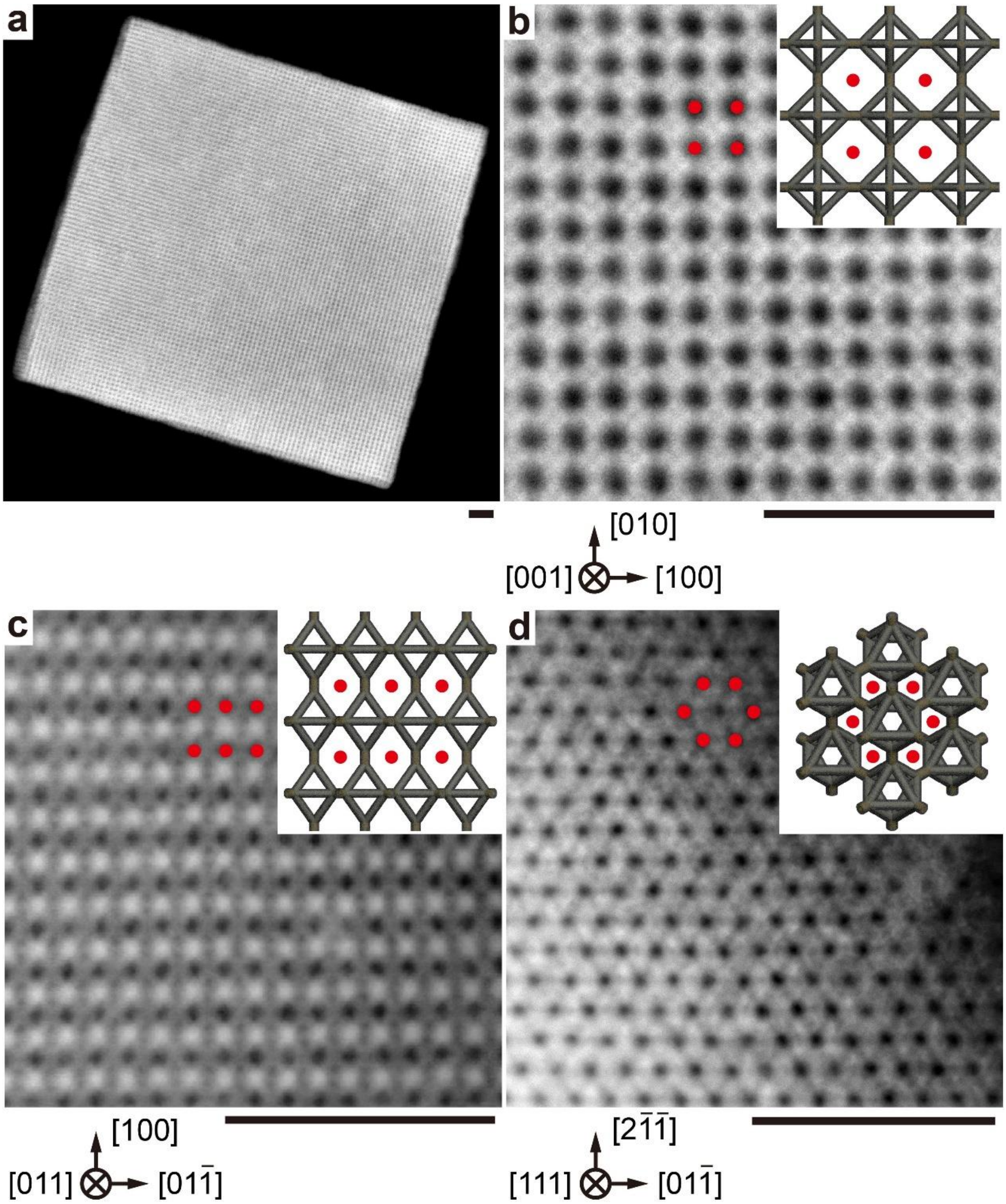

Supplementary Figure 33. Multi-oriented observations of arrangement of R-octa DOFs. The HAADF-STEM images have shown in Fig. 3a in main text and are enlarged for recognition of details in the images. Images are obtained along varying orientations and reveals different orientations of building blocks, which are consistent with the corresponding models (top right panels). Scale bars: 250 nm.

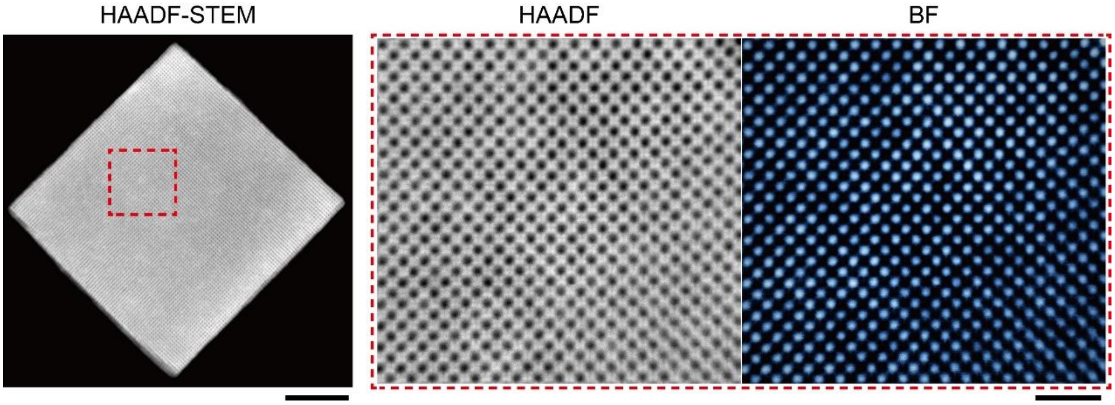

Supplementary Figure 34. STEM characterization of (100) facet of a cubic microcrystal. Partial region is arbitrarily selected (enclosed by red dashed rectangle) from the upper area and amplified under HAADF and bright field (BF) modes, respectively. The cross-shaped organization of R-octa DOF monomers consist with the orientation on (100) facet. Bright spots in the image of BF mode are false coloured for highlighting the contrast. The grain is identical with the one shown in Fig. 3a. Scale bars, zoom out: 1 μm, zoom in: 200 nm.

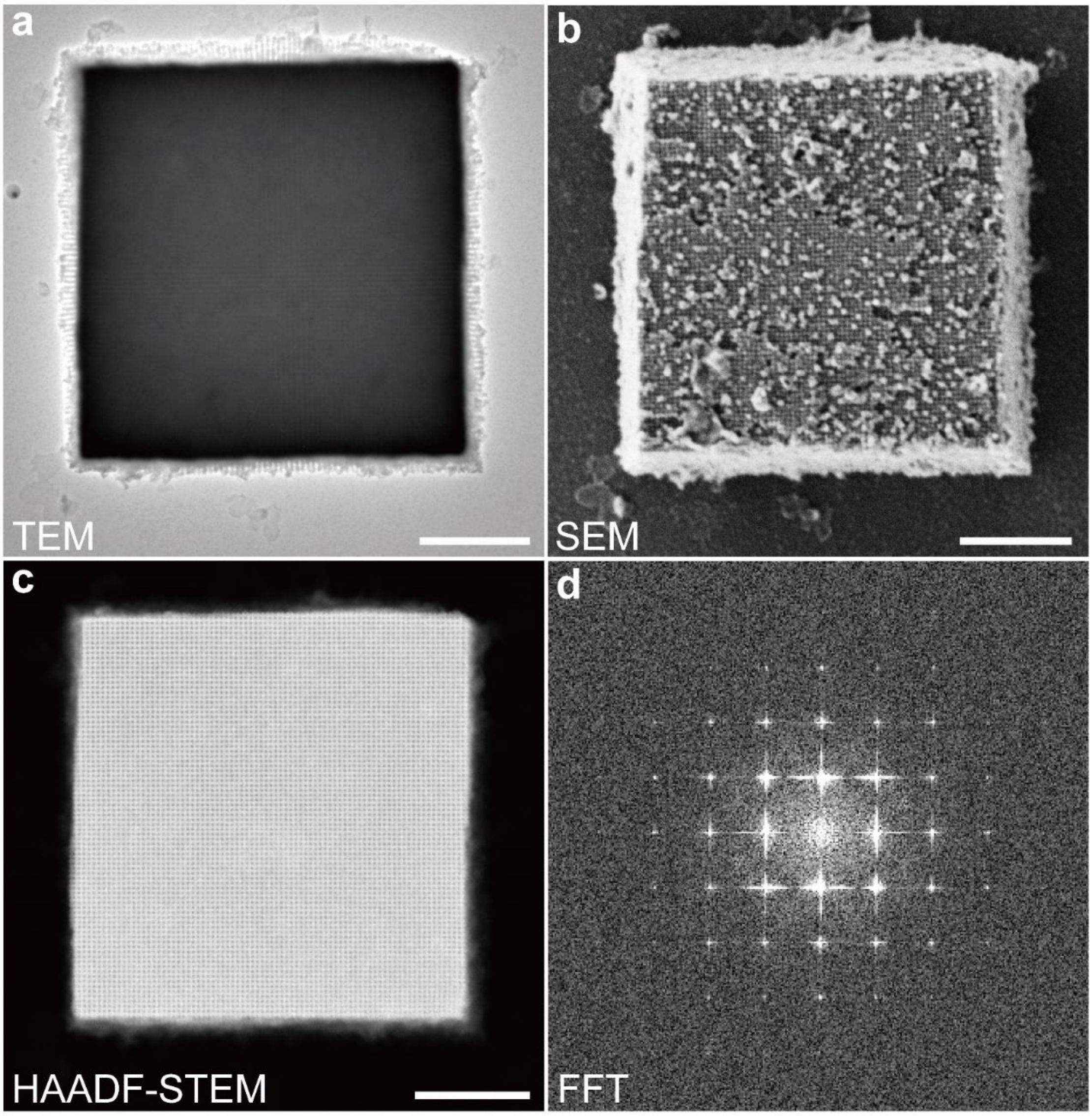

Supplementary Figure 35. Multi-mode characterization of a cubic microcrystal. EM images (**a**: TEM, **b**: SEM, **c**: HAADF-STEM) of a free-standing microcrystal and the corresponding fast Fourier transform (FFT, **d**) calculated from HAADF-STEM image. FFT pattern indicates the periodic structure of the microcrystal. Scale bars, 1 μm.

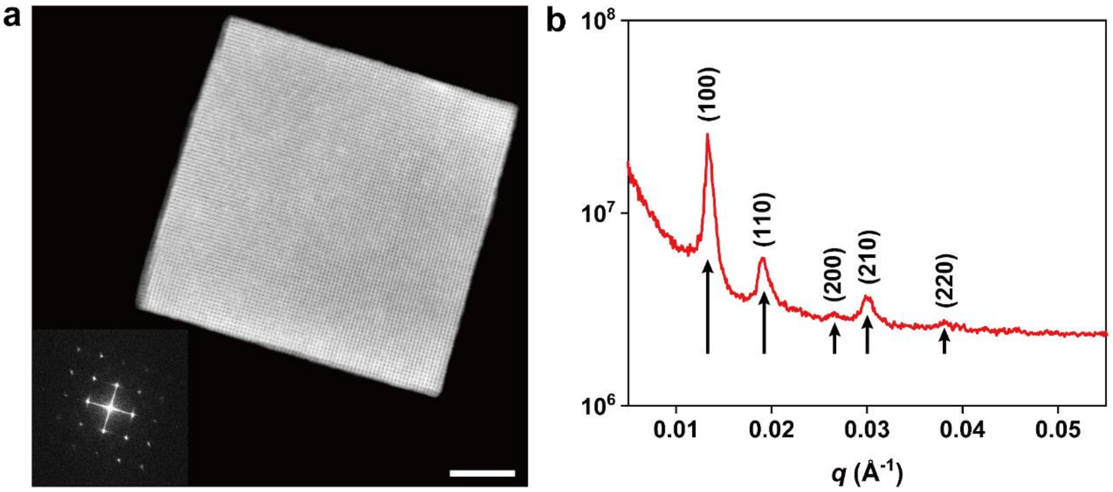

Supplementary Figure 36. FFT analysis of cubic grain and corresponding calibration. a, HAADF-STEM image of a cubic grain and the image is processed by a 2D Fast Fourier transform (FFT, bottom left panel). **b,** Corresponding calibration of FFT. The amplitude after the transform is demonstrated and the radial average of the amplitude is plotted in logarithmic scale. Peaks at the spatial frequency of 0.013, 0.019, 0.027, 0.030 and 0.038 Å^{-1} correspond to (100), (110), (200), (210), (220) lattice facets respectively and are calibrated according to the simple cubic (SC) lattice. The crystal constant is determined as 47 nm. Due to the [100] direction of the incident electron beam, the peak of (111) facet is invisible. The grain is identical with the one shown in Fig. 3a. Scale bar: 1 μm. Source data are provided as a Source Data file.

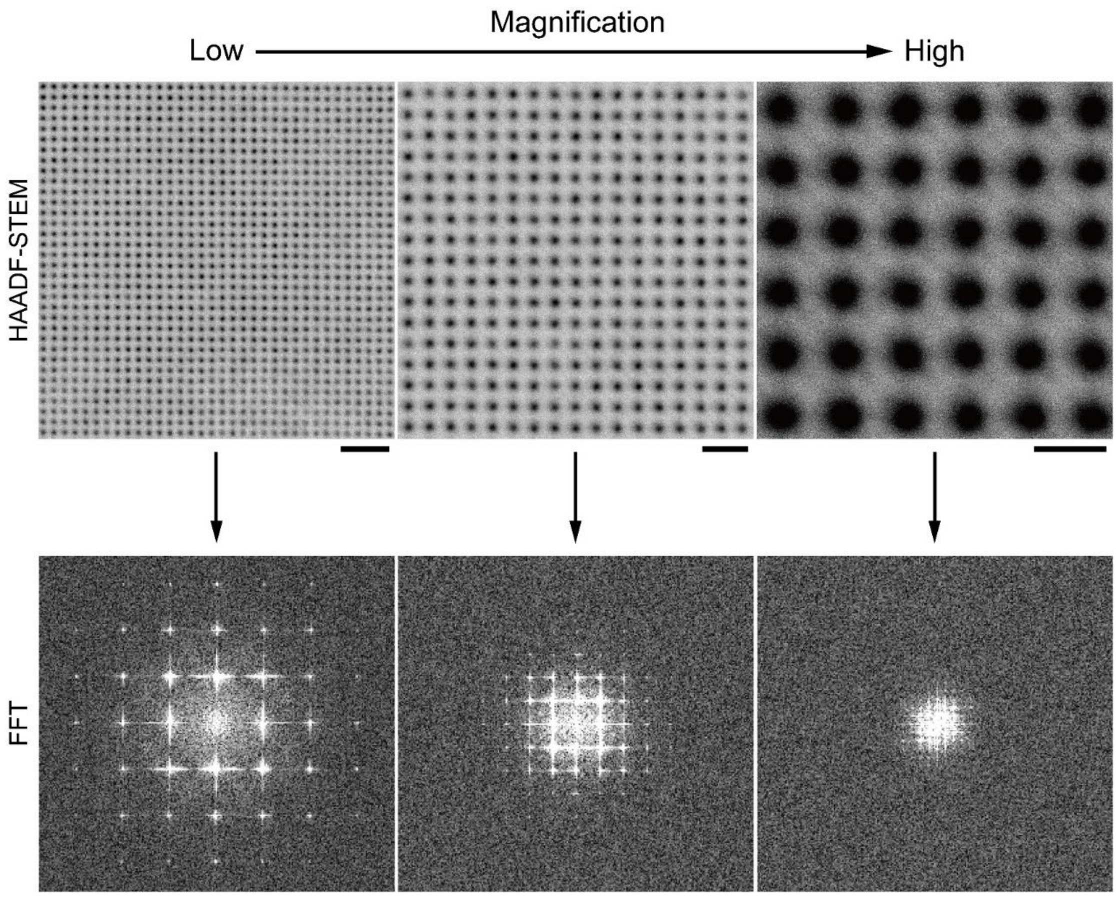

Supplementary Figure 37. Observation of (100) facet of cubic grain in multi-scales. HAADF-STEM images reveal the porous structures of the region which arbitrarily selected from (100) facet of a cubic grain. From left to right, scopes of observation are reduced successively and calculated 2D FFT patterns are shown additionally. Scale bars, from left to right: 200 nm, 100 nm, 50 nm.

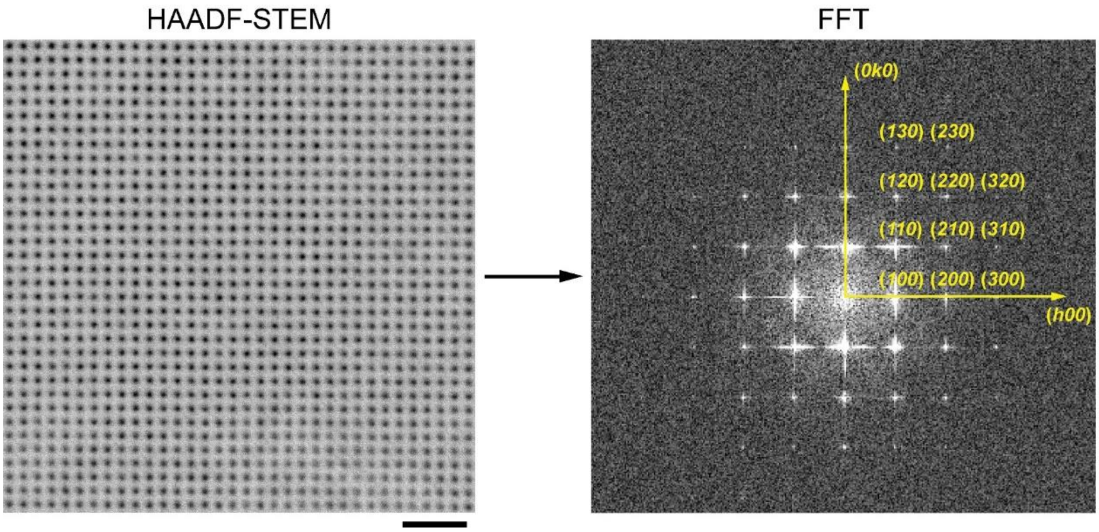

Supplementary Figure 38. Calibration of 2D FFT pattern. STEM image shown in Supplementary Fig. 37 and corresponding calculated FFT pattern (far left panels) are selected to analyze via translating the spots into matched facets. Precise structure of encapsulated R-octa DOF monomers including bundles and corners are hard to identify in such magnification and are recognized to be periodic nodes in FFT calculation. Each independent node is regarded as an encapsulated R-octa DOF and the packing mode is determined to be simple cubic (SC) for guiding the calibration process. Corresponding facets are labeled with Miller index. Scale bar: 200 nm.

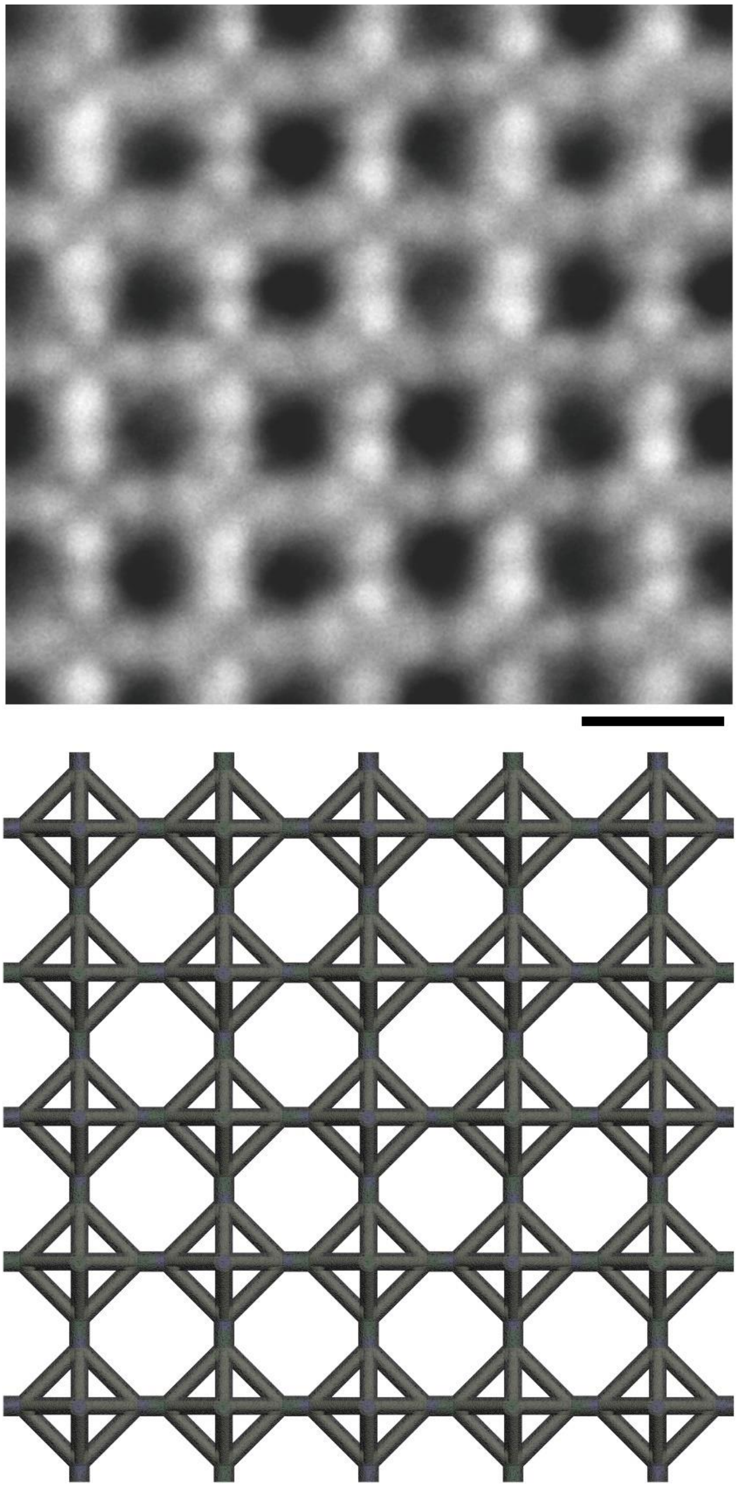

Supplementary Figure 39. Structural determination of (100) facet of an encapsulated cubic grain. HAADF-STEM image is exhibited overlappingly with Fig. 3b for clear showcasing (top panel). The schematic model attached to the image is shown as well for clarifying the geometric configuration and arrangement of R-octa DOFs observed from (100) facet (bottom panel). Scale bar: 50 nm.

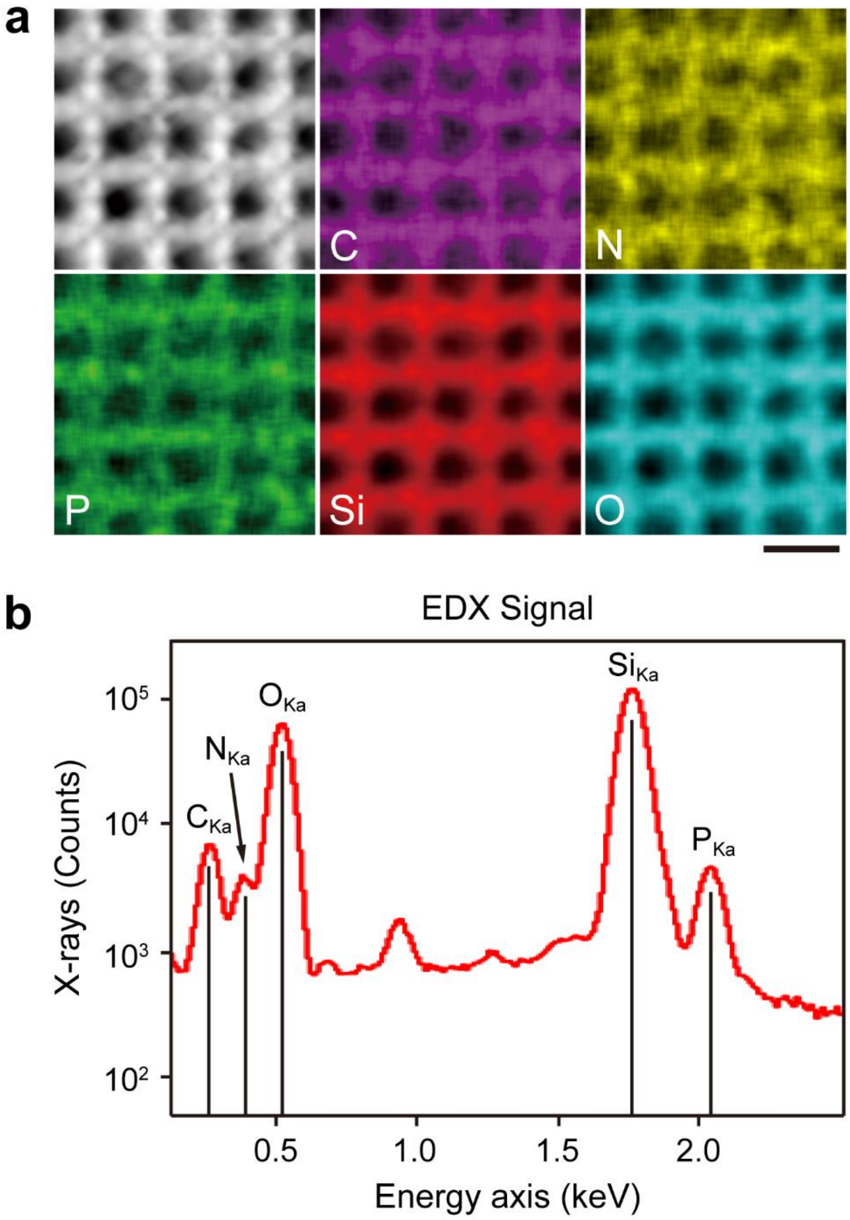

Supplementary Figure 40. High resolution EDS (HR-EDS) analysis of an encapsulated cubic microcrystal. HR-EDS mapping of selected region on (100) facet of solidified cubic grain (**a**) and corresponding integral spectrum (**b**) are shown. Chemical composition marked clockwise from top left is C (purple), N (yellow), O (cyan), Si (red) and P (green), respectively. All the elements shown in mapping images have obvious peaks in corresponding spectrum. EDS mapping is identical with the one in Fig. 3c. Scale bar: 50 nm. Source data are provided as a Source Data file.

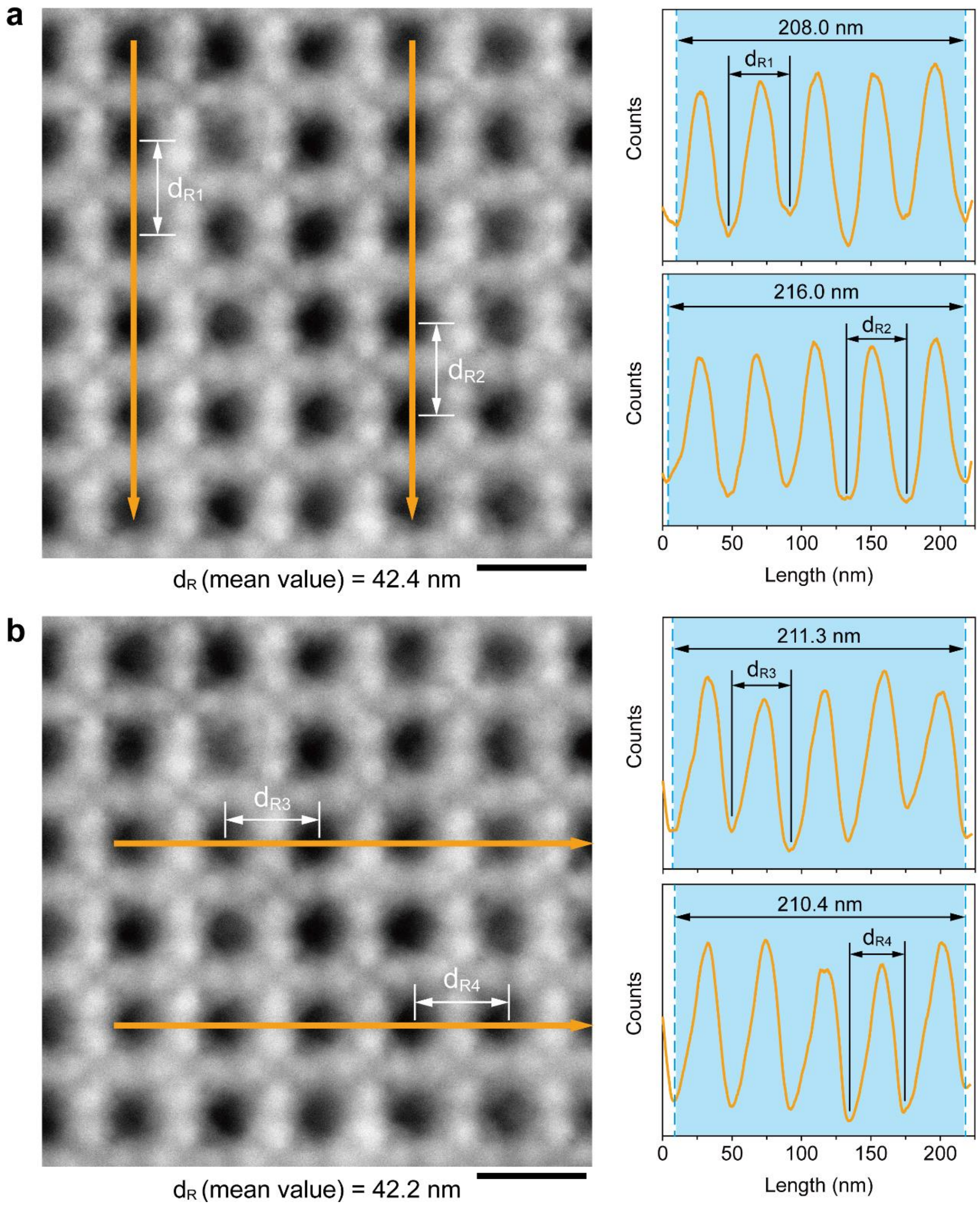

Supplementary Figure 41. Measurement of vertex-to-vertex distances of encapsulated R-octa DOFs. a, HAADF-STEM image of orderly arranged R-octa DOFs observed along [100] orientation. Vertex-to-vertex distances of encapsulated R-octa DOFs (d_R) are measured in two different domains by line profile analysis. Corresponding diagrams are shown beside and approximate mean value is calculated according to the periodic curves. **b,** Two parallel domains which are vertical with the domains in **a** are selected and corresponding parameter is also obtained through the analysis shown beside. Scale bars: 50 nm.

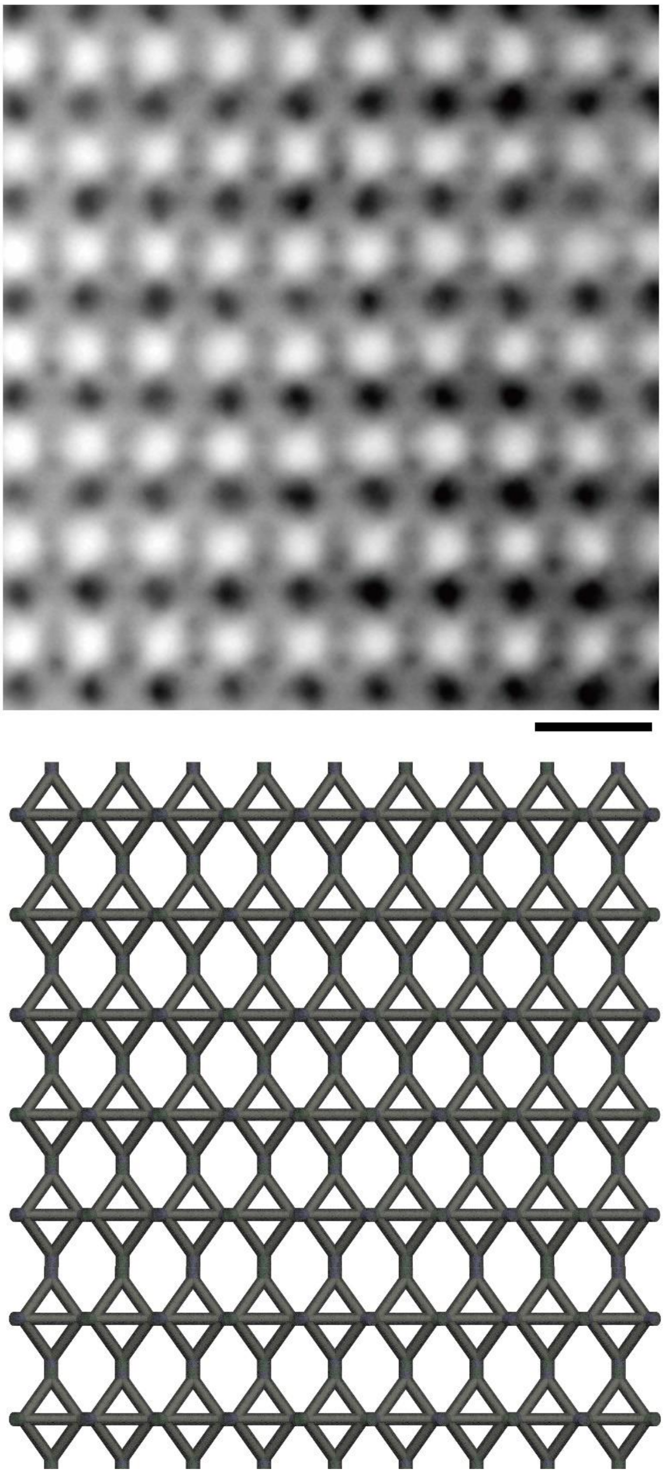

Supplementary Figure 42. Structural determination of (110) facet of an encapsulated cubic grain. Representative HAADF-STEM image reveals the parallel region with the one shown in Fig. 3d for clear showcasing (top panel). The schematic model attached to the image is shown as well for clarifying the geometric configuration and arrangement of R-octa DOFs observed from (110) facet (bottom panel). Scale bar: 50 nm.

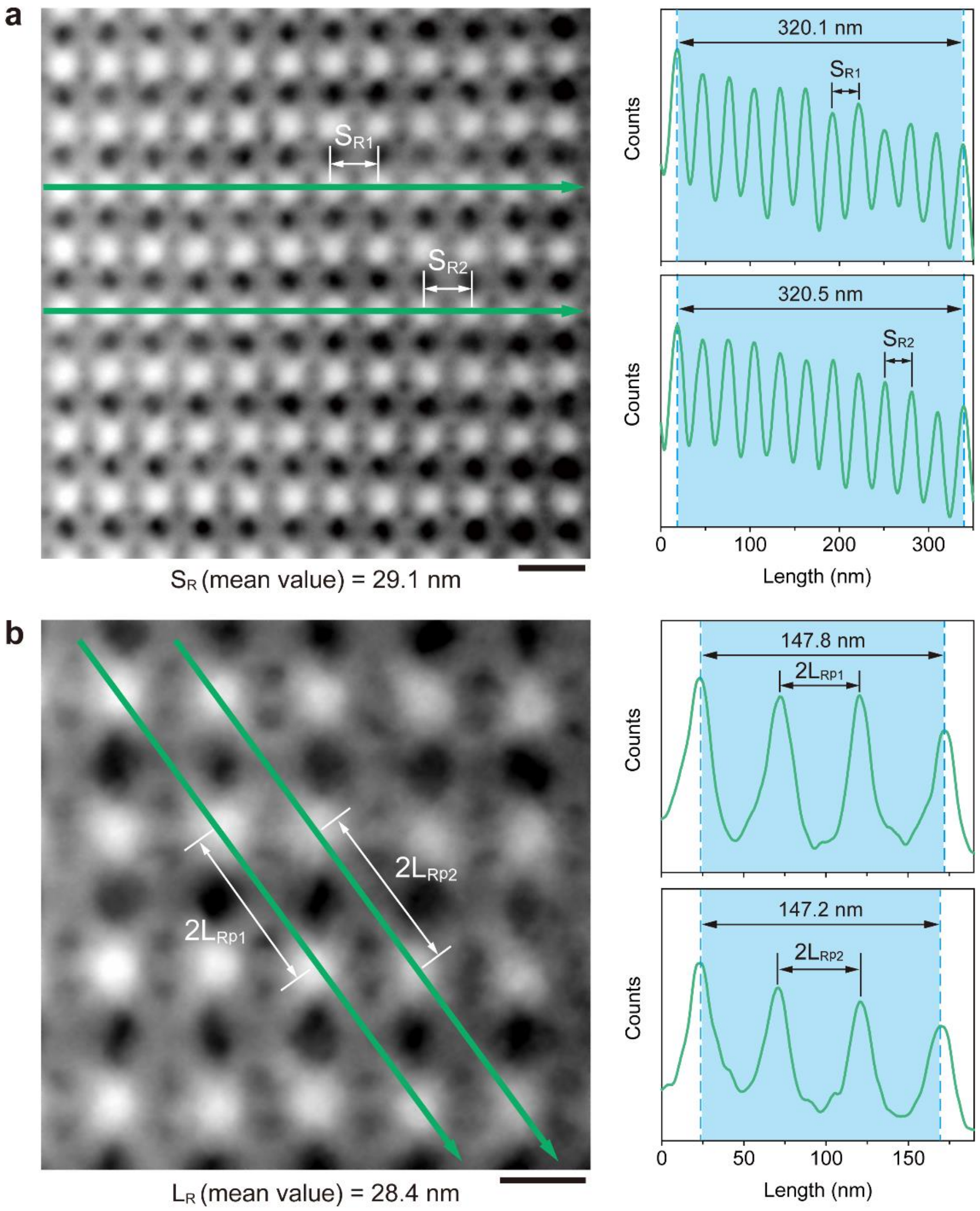

Supplementary Figure 43. Measurement of bundle lengths of encapsulated R-octa DOFs. **a,** HAADF-STEM image of orderly arranged R-octa DOFs observed along [110] orientation. Lengths of encapsulated bundles selected from middle planes of R-octa DOFs (S_R) are measured by line profile analysis. Corresponding diagrams are shown beside and approximate mean value is calculated from the periodic curves. **b,** HAADF-STEM image of orderly arranged R-octa DOFs observed from [110] orientation in a smaller scale. Two parallel domains are selected and corresponding parameter is also obtained through the analysis shown beside. The mean value of L_R is translated from measured lengths of 2D projections (L_{Rp}) of bundles, the meanings of S and L are illustrated in Supplementary Fig. 12. Scale bars, a: 50 nm, b: 25 nm.

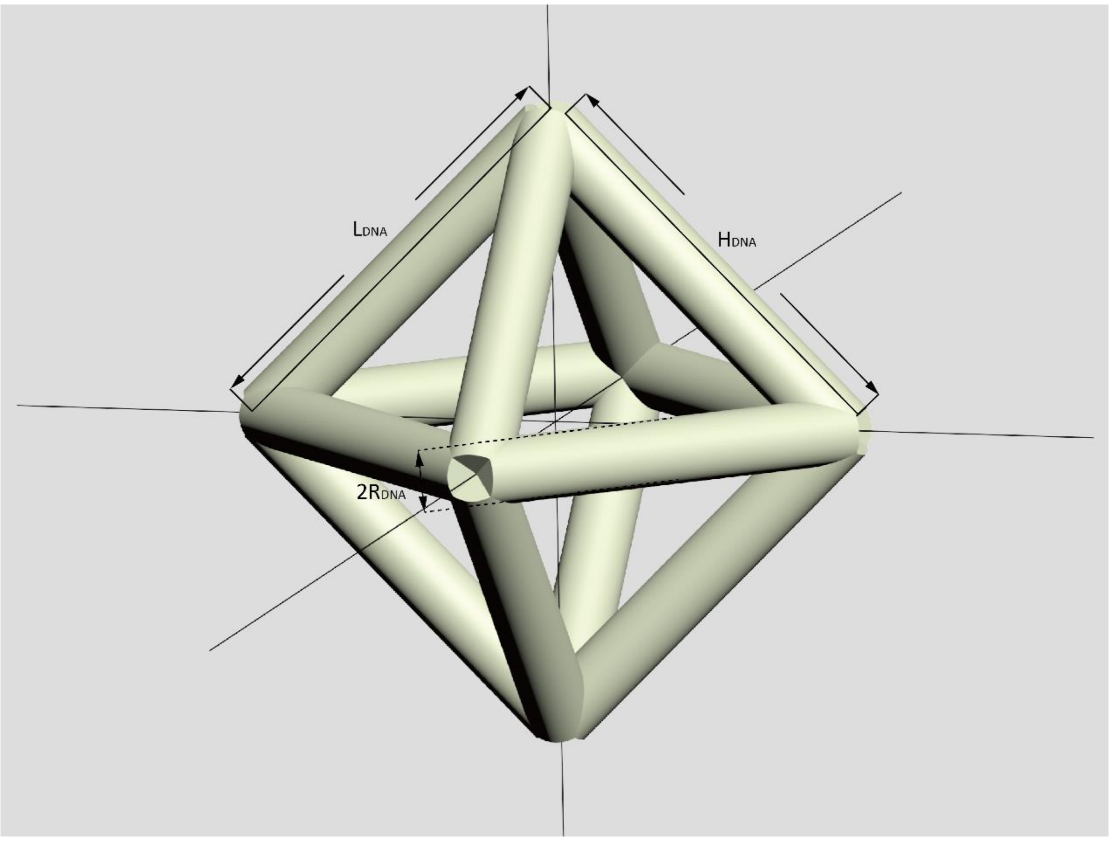

Supplementary Figure 44. Parameter in SAXS modeling. The height (H) and length (L) correspond to height of cylinder and distance of vertex to vertex of octahedra. The R is radius of cylinder model.

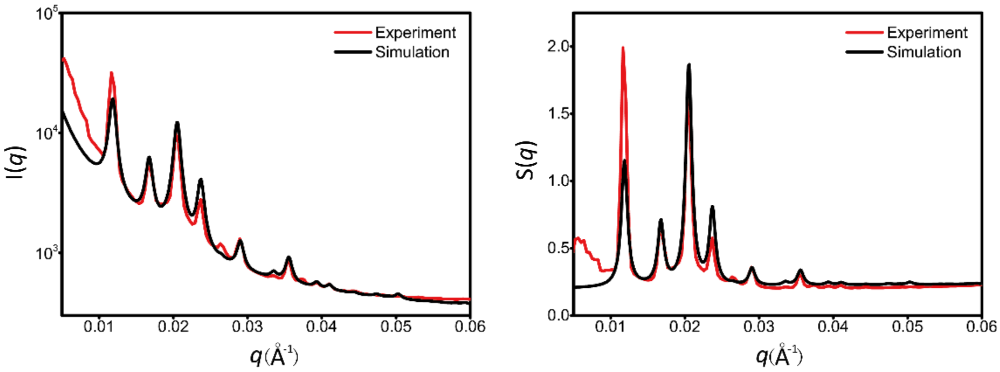

Supplementary Figure 45. The intensity (left) and structure factor (right) of silicified cubic microcrystals. The red curve is experimental data and the black curve is fitting by applying 9 nm as the thickness of the silica encapsulated bundles. Source data are provided as a Source Data file.

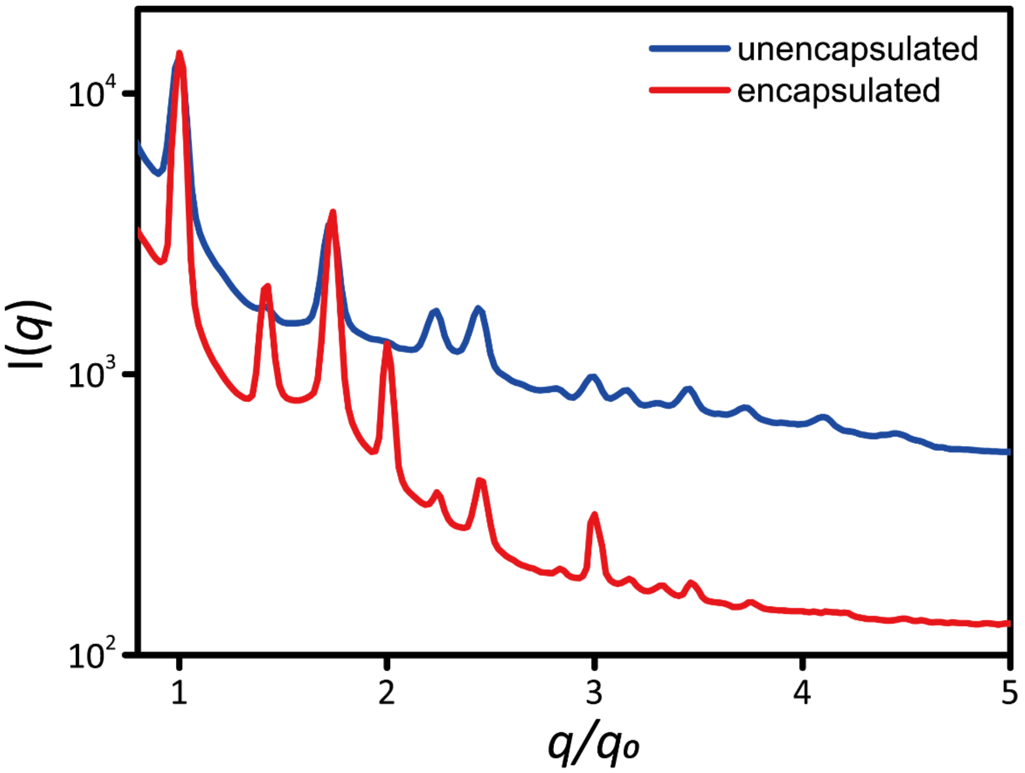

Supplementary Figure 46. SAXS comparison of unencapsulated (blue) and encapsulated (red) DNA origami crystals. q_0 refers to q of the first peak. Source data are provided as a Source Data file.

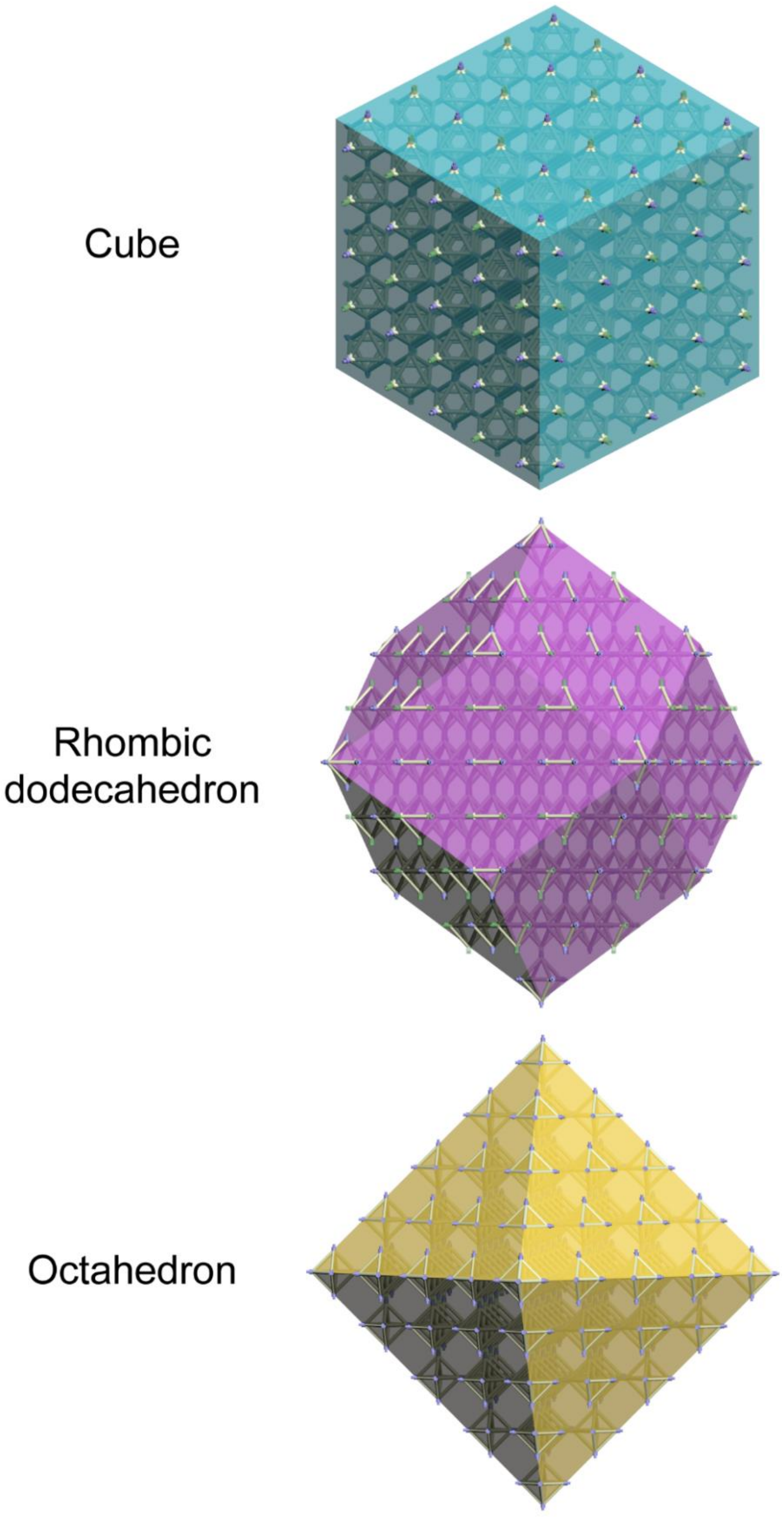

Supplementary Figure 47. Schematic illustration of hypothetical different-shaped microcrystals. Microcrystals with cubic, rhombic dodecahedral and octahedral configurations are assumed to be composed of R-octa DOFs with identical packing modes, which respectively expose {100}, {110} and {111} facets.

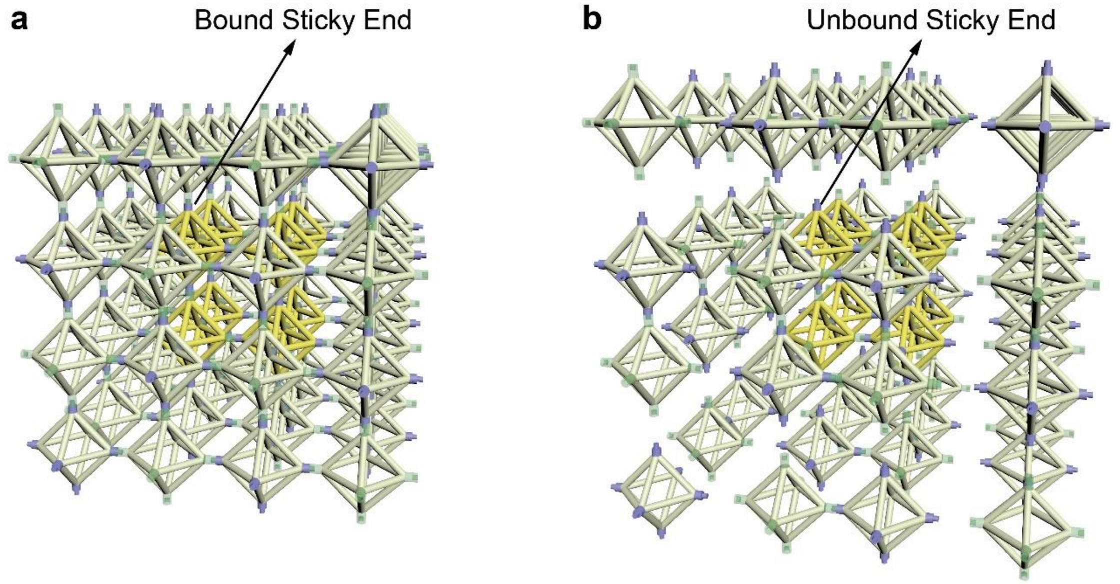

Supplementary Figure 48. Different binding condition for a 2×2×2 segment (marked in yellow). a, 2×2×2 segment as inner portion with bound sticky ends. **b,** 2×2×2 segment as independent portion with unbound sticky ends.

a

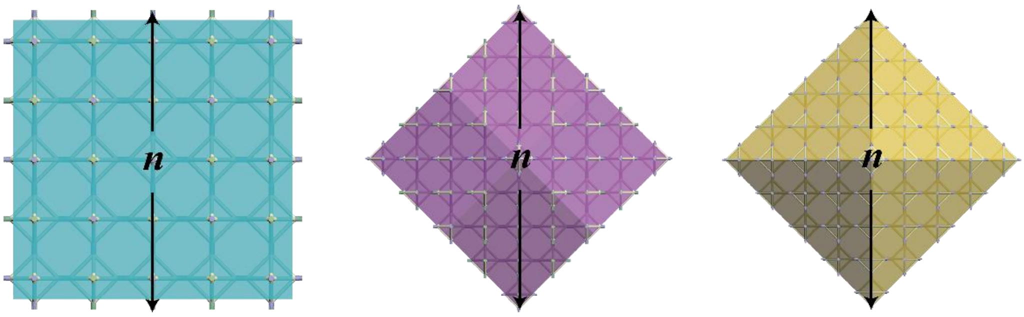

b

Type of crystal habit	Number of DOFs	Number of DOFs with *k* unbound vertexes						Unbound sticky ends
		k=0	*k*=1	*k*=2	*k*=3	*k*=4	*k*=5	
Cube	287,496	262,144	24,576	768	8	0	0	104,544
Rhombic Dodecahedron	289,433	273,207	0	15,612	608	0	6	132,312
Octahedron	280,959	267,033	0	0	13,224	696	6	169,944

Supplementary Figure 49. Meaning of parameter *n* and distribution of DOFs at different position. Parameter of *n* refers to the number of DOFs along the arrow in cube-shaped habit, rhombic-dodecahedron-shaped habit and octahedron-shaped, respectively (**a**) and distribution of DOFs is shown in table (**b**).

a

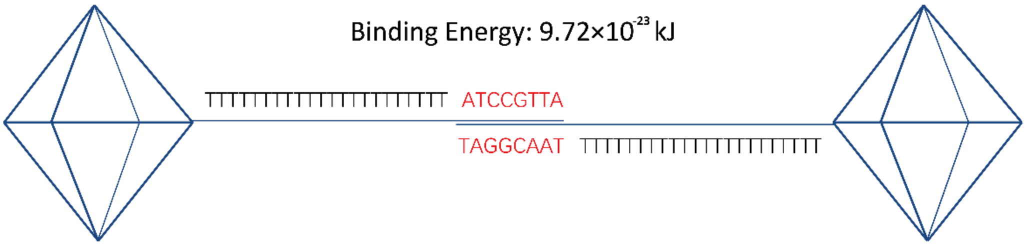

b

Type of crystal habit	Cube	Rhombic Dodecahedron	Octahedron
For a single crystal grain			
Binding energy of the sequence of 8-nt sticky end (kJ)	4.86×10^{-23}		
Number of DOFs in a single crystal grain	287,496	289,433	280,959
Number of unbound sticky ends in a single crystal grain	104,544	132,312	169,944
Surface energy of a single crystal grain (kJ)	5.08×10^{-18}	6.43×10^{-18}	8.26×10^{-18}
In a stated system containing 1 mole DOFs			
Number of crystal grains	2.09×10^{18}	2.07×10^{18}	2.14×10^{18}
Number of free DOFs	1.13×10^{21}	2.87×10^{21}	7.48×10^{20}
Overall energy before crystallization (kJ/mol)	702.17	702.17	702.17
Surface energy after crystallization (kJ/mol)	11.94	16.66	18.58
Reduced energy after crystallization (kJ/mol)	690.23	685.51	683.59

Supplementary Figure 50. Schematic illustration of sticky end cohesion of R-Octa DOFs (a) and surface energy calculation of the three habits (b).

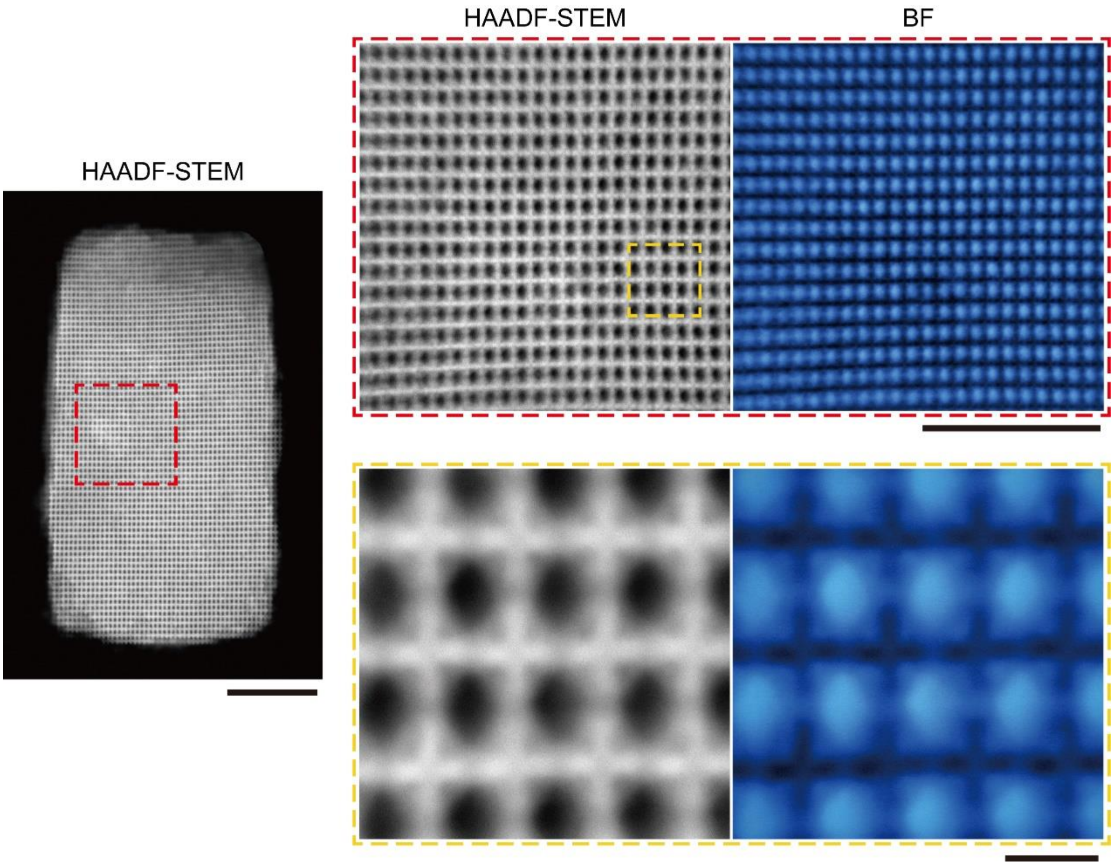

Supplementary Figure 51. STEM characterization of (100) facet of a cuboid microcrystal. Emulating the characterization of cubic microcrystals, familiar observation is carried out upon cuboid crystals. The region enclosed by the red dashed rectangle is arbitrarily selected from (100) facet of the entire microcrystal shown in HAADF-STEM image (left panel) and corresponding BF-STEM image is shown beside and false coloured in blue to highlight the contrast. Additionally, the further inspection of the region enclosed by the yellow dashed rectangle demonstrate the specific structure of encapsulated E-octa DOF monomers. The corresponding BF-STEM image is exhibited beside as well. The entire grain is identical with the one shown in Fig. 4a. Scale bars, image of entire crystal: 1 μm, zoom in: 500 nm (top row) and 100 nm (bottom row).

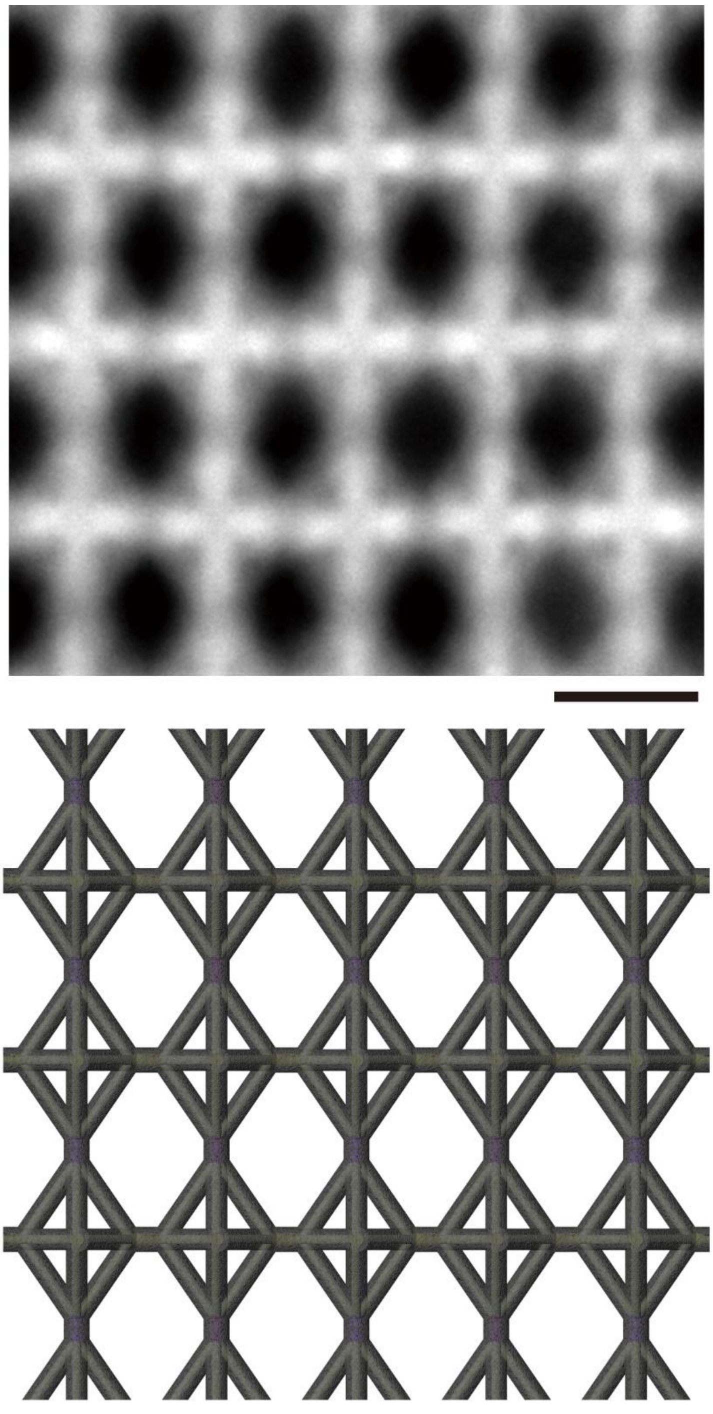

Supplementary Figure 52. Structural determination of (100) facet of an encapsulated cuboid grain. HAADF-STEM image reveals the parallel region with the one shown in Fig. 4b for clear showcasing (top panel). The schematic model attached to the image is shown as well for clarifying the geometric configuration and arrangement of E-octa DOFs observed from (100) facet (bottom panel). Scale bar: 50 nm.

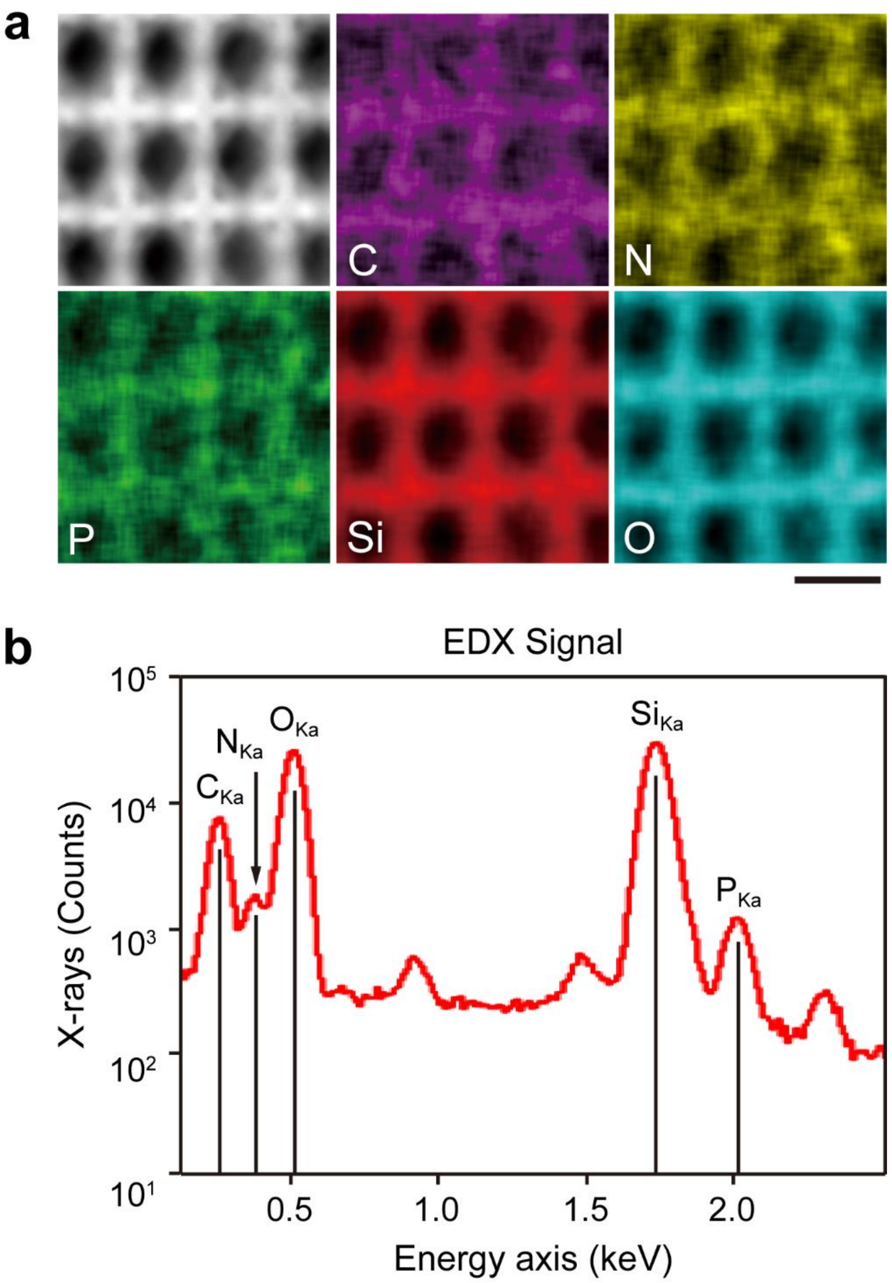

Supplementary Figure 53. High resolution EDS (HR-EDS) analysis of an encapsulated cuboid microcrystal. HR-EDS mapping of selected region on (100) facet of solidified cuboid grain (**a**) and corresponding integral spectrum (**b**) are shown. Chemical composition marked clockwise from top left is C (purple), N (yellow), O (cyan), Si (red) and P (green), respectively. All the elements shown in mapping images have obvious peaks in corresponding spectrum. EDS mapping is identical with the one in Fig. 4c. Scale bar: 50 nm. Source data are provided as a Source Data file.

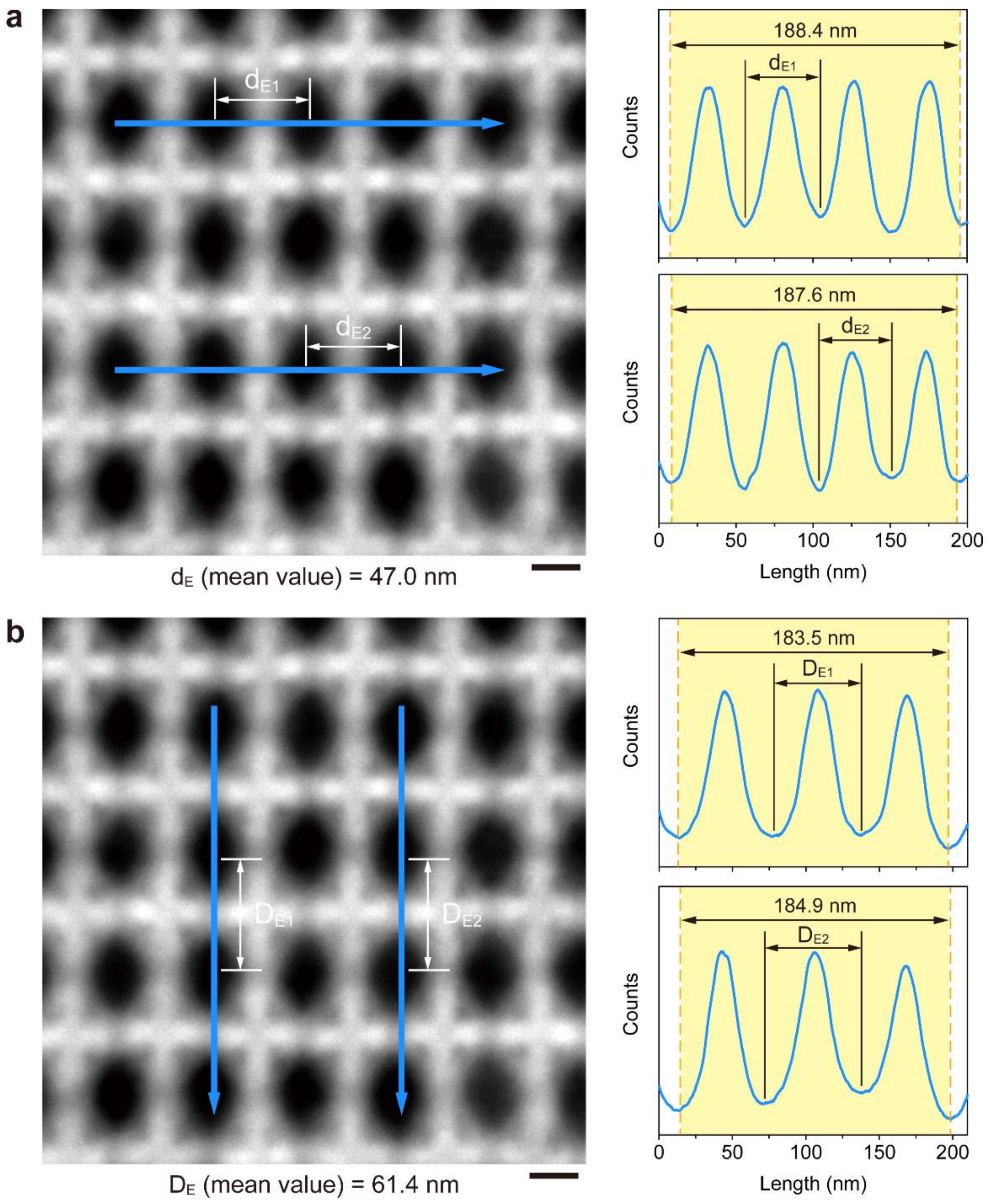

Supplementary Figure 54. Measurement of vertex-to-vertex distances of encapsulated E-octa DOFs. a, HAADF-STEM image of orderly arranged E-octa DOFs observed along [100] orientation. Vertex-to-vertex distances of encapsulated E-octa DOFs along unelongated direction (d_E) are measured in two different domains by line profile analysis. Corresponding diagrams are shown beside and approximate mean value is calculated according to the periodic curves. **b,** Two parallel domains which are vertical with the domains in **a** are selected for measurement of vertex-to-vertex distances of encapsulated E-octa DOFs along elongated direction (D_E), and corresponding parameter is also obtained through the analysis shown beside. The mean value is represented by d_E and D_E which are theoretically unequal ($d_E < D_E$). Scale bars: 25 nm.

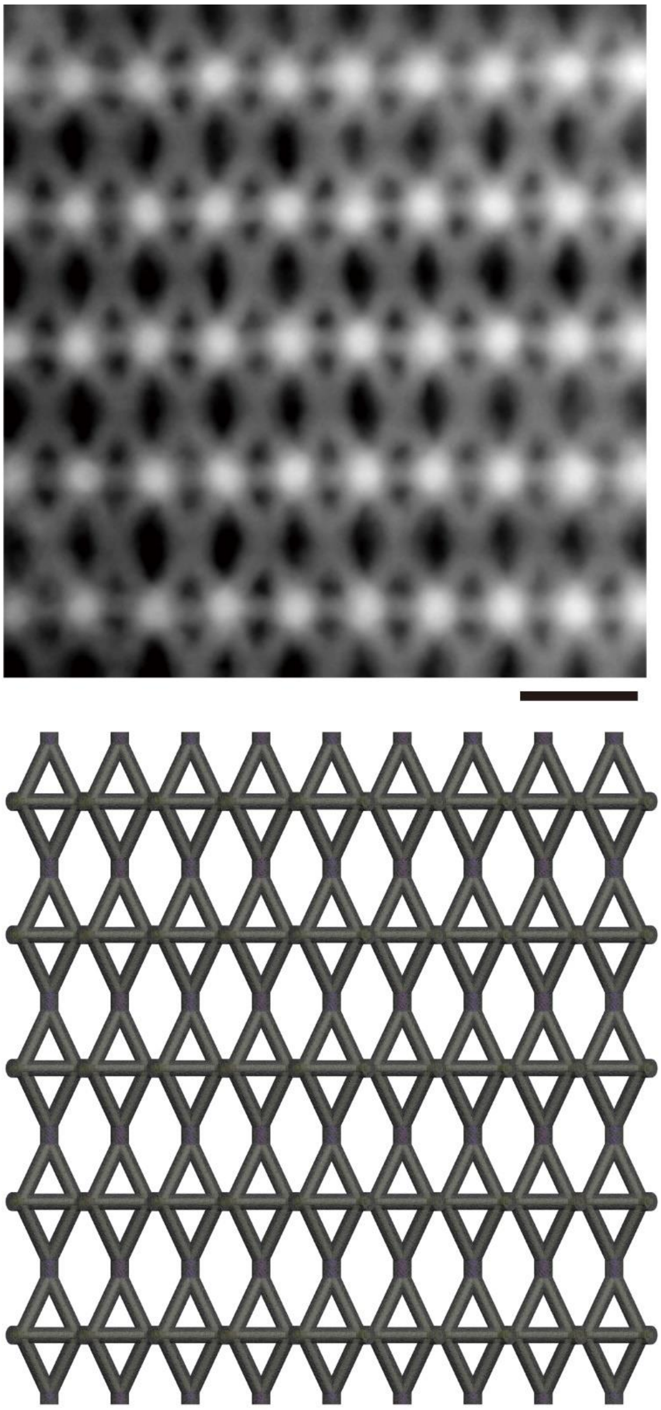

Supplementary Figure 55. Structural determination of an encapsulated cuboid grain along [110] orientation. Representative HAADF-STEM image reveals the parallel region with the one shown in Fig. 4d for clear showcasing (top panel). The schematic model attached to the image is shown as well for clarifying the geometric configuration and arrangement of E-octa DOFs observed along [110] orientation (bottom panel). Scale bar: 50 nm.

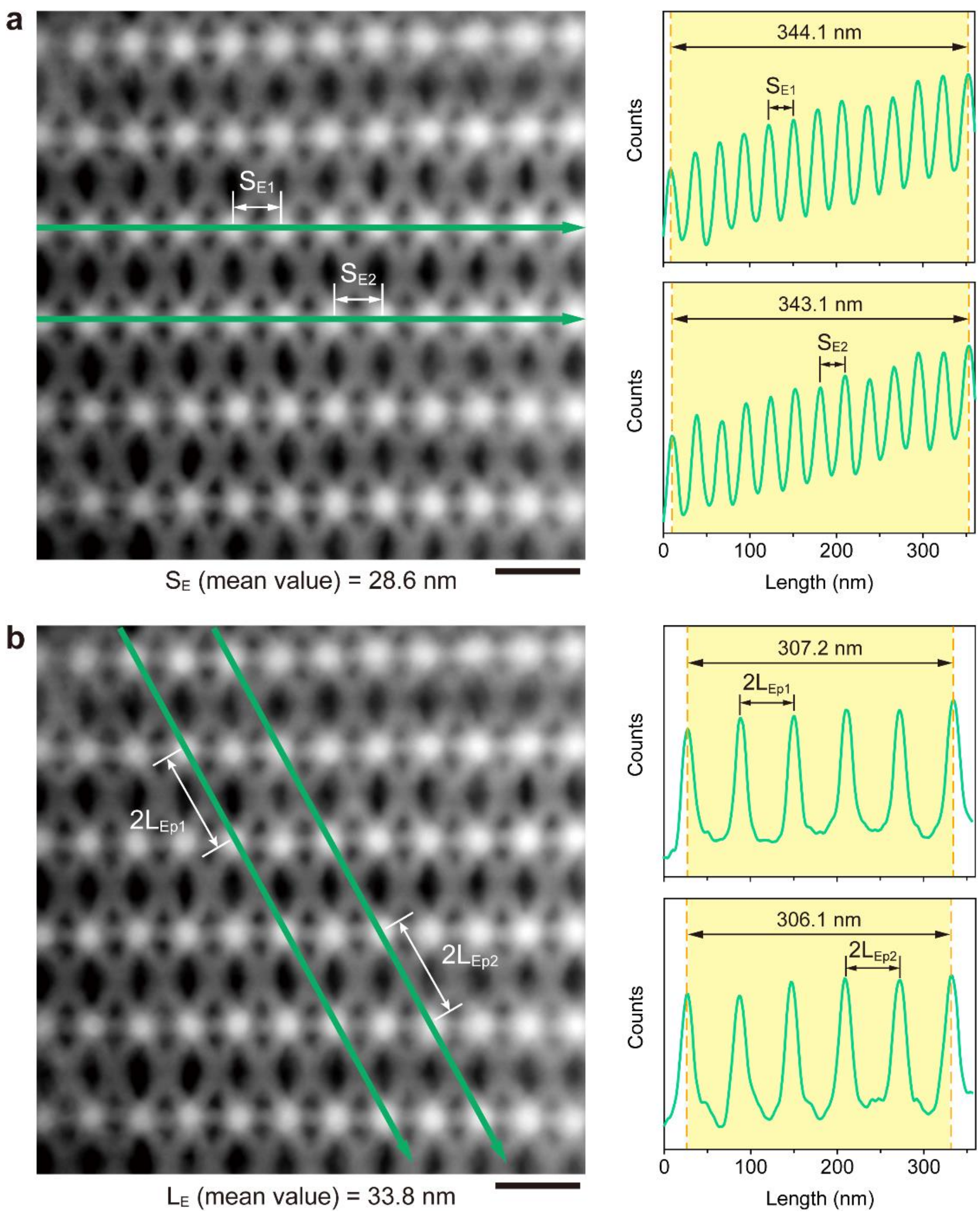

Supplementary Figure 56. Measurement of bundle lengths of encapsulated R-octa DOFs. a, HAADF-STEM image of orderly arranged R-octa DOFs observed along [110] orientation. Lengths of encapsulated bundles selected from middle planes of E-octa DOFs (S_E) are measured by line profile analysis. Corresponding diagrams are shown beside and approximate mean value is calculated from the periodic curves. **b,** Two parallel domains are selected and corresponding parameter is also obtained through the analysis shown beside. The mean value of L_E is translated from measured lengths of 2D projections (L_{Ep}) of bundles, the meanings of S and L are illustrated in Supplementary Fig. 12 as well. Scale bars: 50 nm.

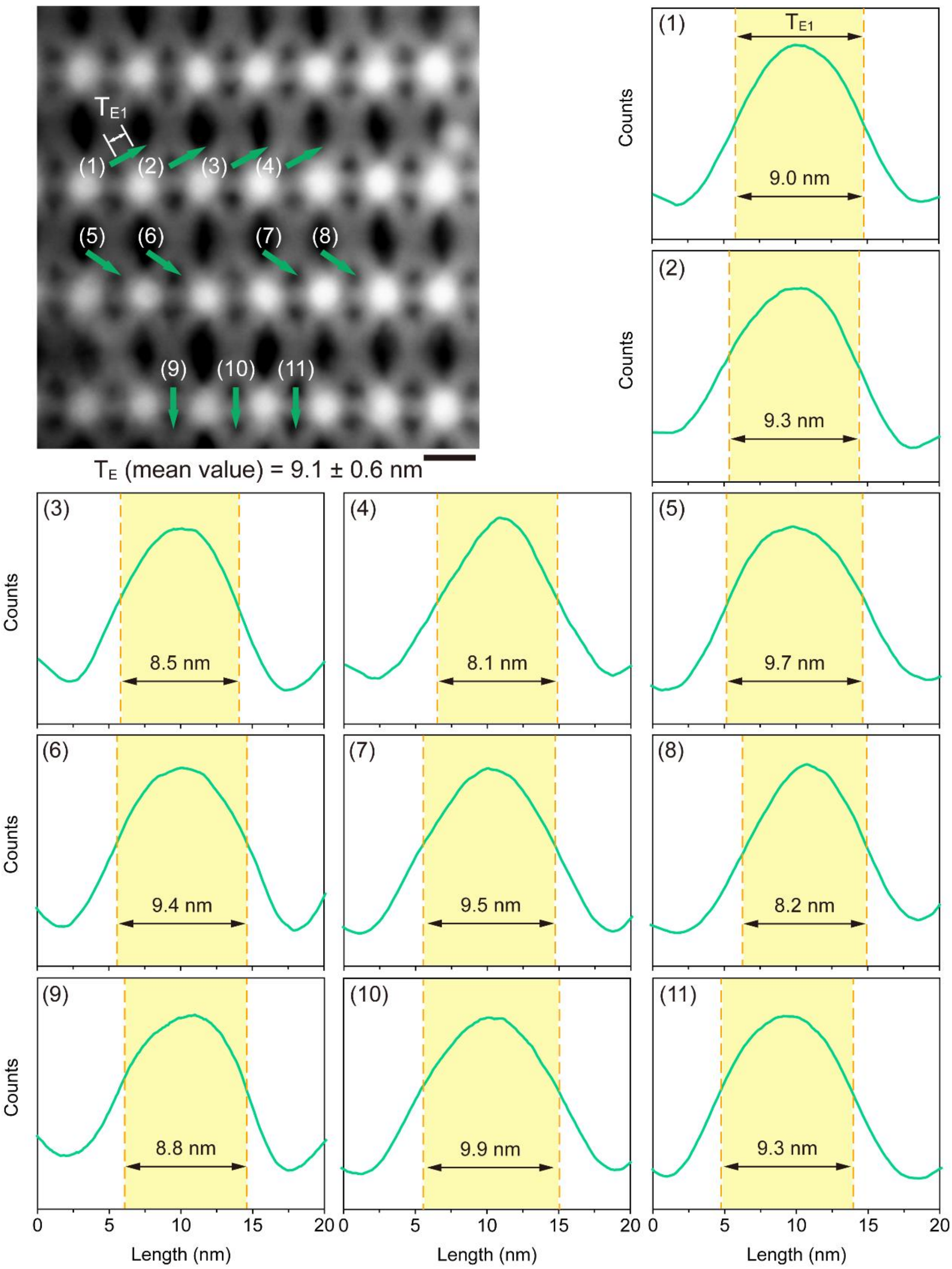

Supplementary Figure 57. Measurement of bundle thickness of encapsulated E-octa DOFs (T_E). Eleven diverse domains (1 to 11) are selected from HAADF-STEM image for line profile analysis. Corresponding diagrams are shown and approximate mean value of T_E is calculated from the peaks. Scale bar: 25 nm.

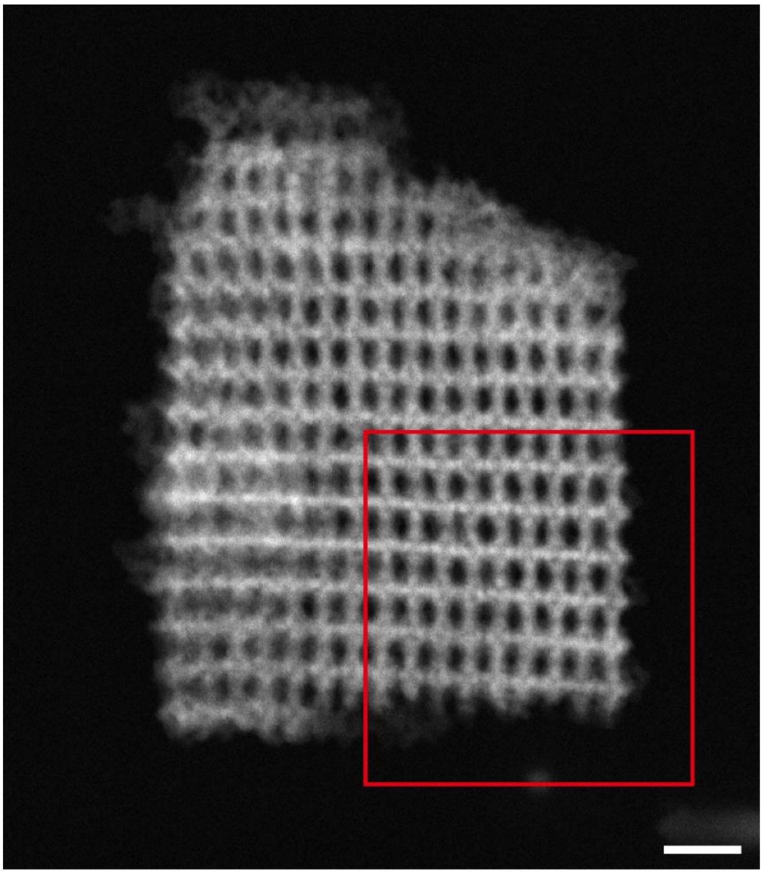

Supplementary Figure 58. HAADF-STEM image of the grain selected for tomographic reconstruction. The original state of a small encapsulated grain composed of E-octa DOFs is recorded for tomography experiment. The fragment is selected from the bottom right hand corner (enclosed by red rectangle) for 3D reconstruction. Scale bar: 100 nm.

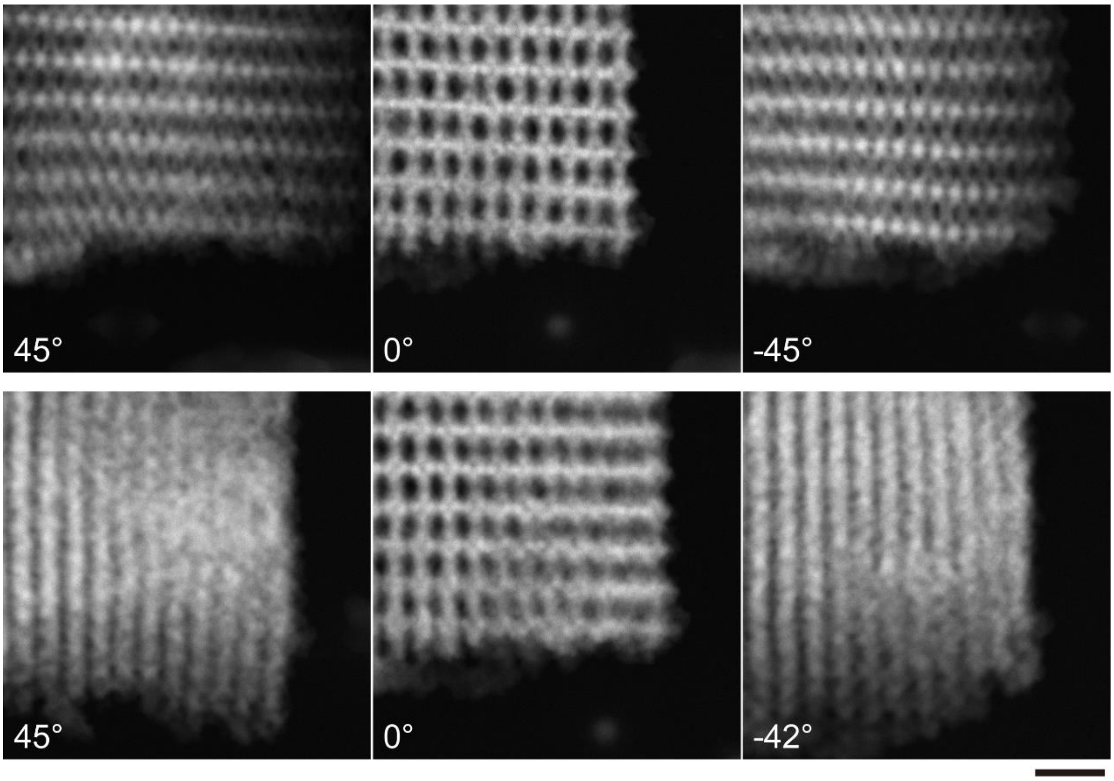

Supplementary Figure 59. Selection of the projections used in tomographic reconstruction. The grain shown in Supplementary Fig. 58 is tilted in 2 perpendicular axes and 86 projections are recorded in total. Three projections of the first axis (-45°, 0°, +45°) are demonstrated in the first row. In the second row, the projections of -42°, 0°, +45° of the second axis are exhibited. Scale bar: 100 nm.

Acceleration voltage: 200 kV Magnification: 57000× (Cubic Microcrystal)			
Serial number	Angle	Serial number	Angle
No.1	-19.1°	No.11	1.18°
No.2	-16.82°	No.12	3.00°
No.3	-15.02°	No.13	5.18°
No.4	-13.08°	No.14	7.01°
No.5	-11.04°	No.15	9.00°
No.6	-9.06°	No.16	11.00°
No.7	-7.03°	No.17	13.00°
No.8	-5.07°	No.18	15.12°
No.9	-3.06°	No.19	17.08°
No.10	-1.05°	No.20	19.11°

Acceleration voltage: 200 kV Magnification: 57000× (Cuboid Microcrystal)			
Serial number	Angle	Serial number	Angle
No.1	-19.01°	No.12	3.02°
No.2	-17.07°	No.13	5.02°
No.3	-15.06°	No.14	7.08°
No.4	-13.06°	No.15	9.11°
No.5	-11.06°	No.16	11.04°
No.6	-9.01°	No.17	13.11°
No.7	-7.06°	No.18	15.02°
No.8	-5.00°	No.19	17.00°
No.9	-2.86°	No.20	19.18°
No.10	-1.07°	No.21	21.05°
No.11	1.02°	No.22	23.39°

Supplementary Figure 60. Specific angles of each image which collected for the composition of Supplementary Video 1 (left panel) and Supplementary Video 2 (right panel). 20 and 22 HAADF-STEM images are obtained for cubic and cuboid microcrystals under the constant tilting. Observation is carried out on a FEI Tecnai F-20 instrument at the accelerating voltage of 200 kV and the specific angle of each image is shown in tables. Obtained images are respectively composited into Supplementary Video 1 (cubic grain) and Supplementary Video 2 (cuboid grain), which reveal the long-range ordering displayed from the edges of grains in dynamic views.

Supplementary References

1. F. Lu, K. G. Yager, Y. Zhang, H. Xin, O. Gang, Superlattices assembled through shape-induced directional binding. *Nat. Commun.* **6**, 6912 (2015).
2. Y. Tian, Y. Zhang, T. Wang, H. L. Xin, H. Li, O. Gang, Lattice engineering through nanoparticle-DNA frameworks. *Nat. Mater.* **15**, 654-661 (2016).
3. Y. Tian, J. R. Lhermitte, L. Bai, T. Vo, H. L. Xin, H. Li, R. Li, M. Fukuto, K. G. Yager, J. S. Kahn, Y. Xiong, B. Minevich, S. K. Kumar, O. Gang, Ordered three-dimensional nanomaterials using DNA-prescribed and valence-controlled material voxels. *Nat. Mater.* **19**, 789-796 (2020).
