# Supplementary material for: DNA origami single crystals with Wulff shapes
Source: Nat Commun. 2021 May 21;12:3011. doi: 10.1038/s41467-021-23332-4 (PMC8140131; doi:10.1038/s41467-021-23332-4)
Supplement: Supplementary file 2 — Description of Additional Supplementary Files [file 41467_2021_23332_MOESM2_ESM.docx]

Description of Additional Supplementary Files

**Supplementary Movie 1. Tilting experiment of the edge part of a discrete encapsulated cubic single crystal visualized via HAADF-STEM.** This animation showcases the entire process of tilting experiment for an encapsulated cubic grain under HAADF-STEM imaging. We focus on the edge of grain during the process and record the change of geometric configurations of R-octa DOFs which display from the image along with the constant rotation. Tilting is carried out from -19.1° to +19.11°, as illustrated in Supplementary Fig. 60.

**Supplementary Movie 2. Tilting experiment of the edge part of a discrete encapsulated cuboid single crystal visualized via HAADF-STEM.** This animation showcases the entire process of tilting experiment for an encapsulated cuboid grain under HAADF-STEM imaging. We focus on the edge of grain during the process and record the change of geometric configurations of E-octa DOFs which display from the image along with the constant rotation. Tilting is carried out from -19.01° to +23.39°, as illustrated in Supplementary Fig. 60.

**Supplementary Movie 3. Tilting process of the segmental crystallite selected for 3D reconstruction along the first rotation axis.** This animation is related to Supplementary Figs. 58, 59. We monitor the continuous segment which is selected from an entire piece composed of solidified E-octa DOFs and subsequently conduct the tilting experiment for further 3D reconstruction of the selected domain. The specimen is driven to successively rotate along one of two mutually perpendicular axes and the entire tilting process is demonstrated in this movie.

**Supplementary Movie 4. Tilting process of the segmental crystallite selected for 3D reconstruction along the second rotation axis.** This animation is related to Supplementary Figs. 58, 59 and Supplementary Movie 3. The monitored specimen is driven to successively rotate along the other rotation axis and the entire tilting process is demonstrated in this movie.

**Supplementary Movie 5. Rotation process of 3D rendered density map of reconstructed segment along the first axis.** 3D reconstruction is accomplished with the help of a suite of 2D projections which showcased in Supplementary Fig. 59. In this animation, we exhibit the computed density map (as shown in Fig. 4f and 4g in main text) by constant rotation along one of three rotation axes for intensive illustration of internal periodic structure.

**Supplementary Movie 6. Rotation process of 3D rendered density map of reconstructed segment along the second axis.** This animation is related to Supplementary Movie 5. In this animation, the computed density map is exhibited by constant rotation along the second of three rotation axes for intensive illustration of internal periodic structure.

**Supplementary Movie 7. Rotation process of 3D rendered density map of reconstructed segment along the third axis.** This animation is related to Supplementary Movie 5 and 6. In this animation, the computed density map is exhibited by constant rotation along the third of three rotation axes for intensive illustration of internal periodic structure.

**Supplementary Movie 8. Tomography of the segmental crystallite selected for 3D reconstruction along the first orientation.** This animation is related to Fig. 4f in main text. Rendered 3D density map is intercepted by a red-coloured plane. We reciprocate the plane and obtain manifold corresponding cross-sectional images, then these images are composited into this animation.

**Supplementary Movie 9. Tomography of the segmental crystallite selected for 3D reconstruction along the second orientation.** This animation is related to Fig. 4g in main text. Rendered 3D density map is intercepted by a green-coloured plane which is vertical to the red plane. We reciprocate the plane and obtain manifold corresponding cross-sectional images, then these images are composited into this animation.
